# Genome-Wide Survey and Expression Analysis of the Putative Non-Specific Lipid Transfer Proteins in *Brassica rapa* L

**DOI:** 10.1371/journal.pone.0084556

**Published:** 2014-01-31

**Authors:** Jun Li, Guizhen Gao, Kun Xu, Biyun Chen, Guixin Yan, Feng Li, Jiangwei Qiao, Tianyao Zhang, Xiaoming Wu

**Affiliations:** Oil Crops Research Institute of the Chinese Academy of Agricultural Sciences, Key Laboratory of Biology and Genetic Improvement of Oil Crops, Ministry of Agriculture, Wuhan, Hubei, People's Republic of China; Nanjing Agricultural University, China

## Abstract

**Background:**

Plant non-specific lipid transfer proteins (nsLtps) are small, basic proteins encoded by multigene families and have reported functions in many physiological processes such as mediating phospholipid transfer, defense reactions against phytopathogens, the adaptation of plants to various environmental conditions, and sexual reproduction. To date, no genome-wide overview of the *Brassica rapa nsLtp* (*BrnsLtp*) gene family has been performed. Therefore, as the first step and as a helpful strategy to elucidate the functions of BrnsLtps, a genome-wide study for this gene family is necessary.

**Methodology/Principal Finding:**

In this study, a total of 63 putative *BrnsLtp* genes were identified through a comprehensive *in silico* analysis of the whole genome of *B. rapa*. Based on the sequence similarities, these BrnsLtps was grouped into nine types (I, II, III, IV, V, VI, VIII, IX, and XI). There is no type VII nsLtps in *B. rapa*, and a new type, XI nsLtps, was identified in *B. rapa*. Furthermore, nine type II *AtLtps* have no homologous genes in *B. rapa*. Gene duplication analysis demonstrated that the conserved collinear block of each *BrnsLtp* is highly identical to those in Arabidopsis and that both segmental duplications and tandem duplications seem to play equal roles in the diversification of this gene family. Expression analysis indicated that 29 out of the 63 *BrnsLtps* showed specific expression patterns. After careful comparison and analysis, we hypothesize that some of the type I BrnsLtps may function like Arabidopsis pathogenesis-related-14 (PR-14) proteins to protect the plant from phytopathogen attack. Eleven *BrnsLtps* with inflorescence-specific expression may play important roles in sexual reproduction. Additionally, *BrnsLtpI.3* may have functions similar to Arabidopsis *LTP1*.

**Conclusions/Significance:**

The genome-wide identification, bioinformatic analysis and expression analysis of *BrnsLtp* genes should facilitate research of this gene family and polyploidy evolution and provide new insight towards elucidating their biological functions in plants.

## Introduction

Lipids are important chemicals involved in many aspects of development and growth in plants. The most common examples are the surface layers, cutin and suberin. These structures are made up of hydrophobic polyesters of fatty acid derivatives and are known to be associated with biotic and abiotic stress. Additionally, the lipids deposited in storage organs, such as seeds and fruits, are a crucial energy source for the growing populations of the world. Lipids and their derivatives also play roles in many important cell-signaling pathways [1]. More than 1000 chemically distinct lipid species are known to exist in eukaryotic cells [2], and these lipids can be classified into three major classes: glycerophospholipids, sphingolipids and sterols [3]. As the basic constituents of transport vesicles, most lipids are thought to be transported between organelles by vesicular transport. However, lipid transportation can also be detected when vesicular transport is impaired by ATP depletion and upon reduction in temperature or treatment with specific pharmacological drugs (e.g., colchicine and brefeldin A) [4], [5]. Additionally, lipid transport between organelles that does not occur via vesicular transportation has also been observed [6], [7].

Plant non-specific lipid transfer protein (nsLtp) was first isolated by gel filtration from potato tuber homogenates, and it was found that this protein stimulates the exchange of phospholipids between microsomal fractions and mitochondria [8]. The term plant “non-specific lipid transfer proteins” indicates that these Ltps can bind with various phospholipids with broad specificity [9]. Plant nsLtps are able to transfer phosphatidylcholine, phosphatidylinositol and phosphatidylglycerol from liposomes to mitochondria [10], and they also have the ability to transfer galactolipids. Additionally, nsLtps from some species, such as oilseed rape, spinach, and sunflower, are able to bind acyl-CoA [9], [11], [12]. The ability of plant nsLtps to bind fatty acids or acyl-CoA esters was determined by temperature-dependent ligand affinity or by separation of the acyl-Ltp complex by gel filtration [12]. Several isoforms of nsLtp in oilseed rape revealed the ability to both transfer phosphatidylcholine and bind oleoyl-CoA [9]. In the castor bean, the saturating binding capacities for oleic acid and oleoyl-CoA per mole of Ltp were 1:1 [12]. Taken together, this suggests that nsLtps have dual abilities to bind acyl chains and to transfer lipids.

It is generally accepted that amino acid sequences determine the spatial structures of proteins as well as their properties or functions. All known plant nsLtps are synthesized as precursors with an N-terminal signal peptide, and their mature proteins are small and basic characterized by an eight cysteine motif (ECM) as follows: C-Xn-C-Xn-CC-Xn-CXC-Xn-C-Xn-C [13]. The cysteine (Cys) residues are arranged in four disulfide bonds to stabilize the tertiary structure of the hydrophobic cavity, the size plasticity of which allows for the binding of different lipid and hydrophobic compounds *in vitro* [14]. Plant nsLtps can be classified according to their molecular masses (MM) into two main types, nsLtp1 (9 kDa) and nsLtp2 (7 kDa) [14]. Computational and biochemical analyses have indicated that type 1 nsLtps are capable of accommodating lipids such as palmitic acid (C16:0) and the phospholipidacyl chains of 1, 2-dimyristoylphosphatidylglycerol [15], [16]. Nevertheless, less is currently known about the lipid binding abilities of type 2 nsLtps. Plant nsLtps from Arabidopsis, rice and Solanaceae can also be divided into several types (I, II, III, IV, V, VI, VII, VIII, IX, X and nsLTPY) based on their sequence similarity [17], [18].

Plant nsLtps were first proposed to be involved in membrane biogenesis as they were shown to transfer lipids between membranes *in vitro* [10]. However, an increasing number of studies have shown that almost all the nsLtps are extracellularly located and are secreted, and a possible role for these proteins in intracellular lipid transfer seems unlikely [19]. All known precursors of the nsLtps are tagged with N-terminal signal peptides, generally 21 to 27 amino acids in length for the type 1 family and 27 to 35 amino acids in length for the type 2 family [20], indicating they are secreted proteins. Thus, nsLtps may be involved in a range of other biological processes. Notwithstanding the amount of data available, the exact functions of nsLtps remain unclear. Multiple physiological functions of nsLtps have been suggested, including cutin synthesis [21]–[23], somatic embryogenesis [24]–[26], pollen development [27], [28], stigma and pollen adhesion [29]–[31], pollen tube growth and guidance [32], [33], cell wall extension [34], biotic stresses [23], [35]–[41], abiotic stresses [42]–[44], plant signaling [1], [45], [46], and seed maturation [47].

Plant nsLtp family is one of the most well-known protein families and is widely distributed in the plant kingdom. Boutrot et al. identified and classified 267 nsLtps sequences [17]. Recently, Wang et al. made a great contribution to the knowledge of plant nsLtps by constructing a systematic plant non-specific lipid transfer protein database (nsLTPDB), and they identified 595 nsLtps from 121 species [48]. However, the number of identified nsLtps in some species is still very low, such as in *Brassica rapa*, where only five members have been identified in nsLTPDB. Therefore, it is necessary to continue to identify nsLtps in these species. A subspecies of Chinese cabbage, *Brassica rapa* subsp. *Pekinensis*, originates from China and is one of the most economically significant vegetable crops in Asia. Additionally, its ‘A’ genome is an important resource for studying the evolutionary history from *Arabidopsis thaliana* to *B. rapa*, *Brassica napus*, or *Brassica juncea*, as well as underpinning the genetic improvement of *Brassica*-related crops. The recent release of the genome of accession Chiifu401–42 of *B. rapa* has enabled us to comprehensively identify and bioinformatically analyse the putative nsLtps in *B. rapa*. In the present study, we took advantage of the available *B. rapa* genome sequence to perform a genome-wide analysis of the putative nsLtps in this species. In total, we identified 63 genes encoding putative nsLtps in the *B. rapa* genome that could be classified into nine types based on the diversity of ECMs. We also conducted phylogenetic and gene duplication analyses of BrnsLtps. Furthermore, we employed publicly available UniGene data and quantitative RT-PCR data to analyse the expression patterns of these genes. The results of this study may guide research involving all members of the nsLtps family and facilitate our understanding of the influence of polyploidy on the evolution of plants.

## Results

### Identification of putative *nsLTP* genes in the *B. rapa* genome

**Figure 1:**
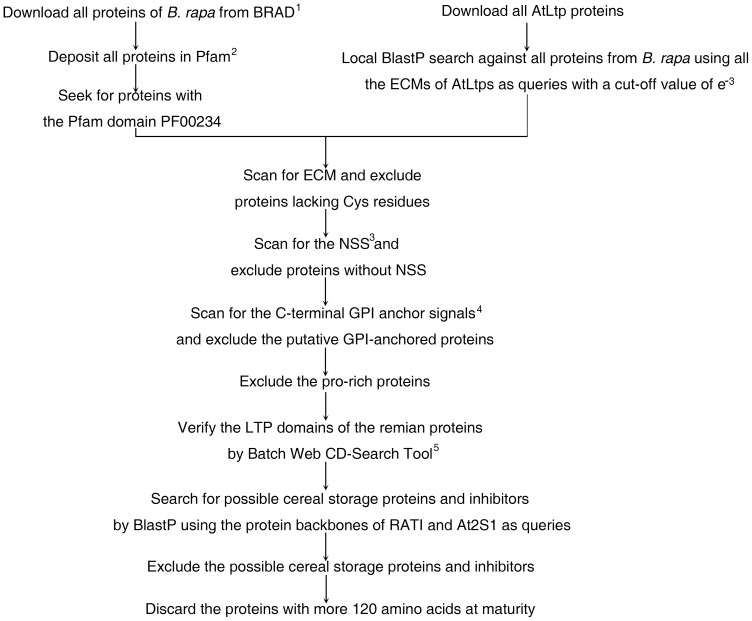
The workflow of BrnsLtp identification and data mining. 1, BRAD, *Brassica* Database, http://brassicadb.org/brad/; 2, http://pfam.sanger.uk/; 3, NSS (N-terminal signal sequence) prediction, http://www.cbs.dtu.dk/services/SignalP; 4, GPI (glycosylphosphatidylinositol) anchor signal prediction, http://mendel.imp.ac.at/gpi/plant_server.html and http://psort.hgc.jp/form.html; 5, LTP domain prediction, http://www.ncbi.nlm.nih.gov/Structure/bwrpsb/bwrpsb.cgi.

Previously, five *nsLtp* genes of *B. rapa* (GI numbers: 122939101, 48093506, 21591782, 3062791, and 1209260) were identified in nsLTPDB. Given that the whole genome of *B. rapa* is now available, we attempted to identify the entire collection of putative *nsLtp* genes in the *B. rapa* genome. Initially, a total of 152 protein sequence (PF00234: plant lipid transfer/seed storage/trypsin-alpha amylase inhibitor) were retrieved after all protein sequences from *B. rapa* (.pep file) were submitted to the Pfam database (Fig. 1). Then, 14 proteins lacking the Cys residues were omitted from the remaining set after manually scanning for the presence of the eight essential Cys residues (Fig. 1). After that, nine proteins lacking N-terminal signal sequences (NSS) and 22 proteins possessing C-terminal glycosylphosphatidylinositol (GPI) anchors were also excluded (Fig. 1). Subsequently, 32 proline-rich or hybrid proline-rich proteins, which are characterized by a high proportion of proline, histidine and glycine residues in the sequence located between the NSS and the ECM [49], [50], were also removed (Fig. 1). Next, the remaining 75 proteins were submitted to the Batch Web CD-Search Tool for verification of their LTP domains, and all but two proteins (Bra001685 and Bra035574) were found to possess LTP domains (Fig. 1). Subsequently, eight proteins (Bra006444, Bra010409, Bra019064, Bra019067, Bra026373, Bra026374, Bra026375, and Bra038483) similar to cereal storage proteins or trypsin-alpha amylase inhibitors were also discarded (Fig. 1). As the mature proteins of nsLtps have low molecular weight, six predicted mature proteins (Bra018484, Bra027016, Bra030873, Bra033087, Bra034559, and Bra037988) with more than 120 amino acids were not taken into consideration (Fig. 1). Finally, a search for misannotated putative *nsLtp* genes was conducted by local BlastP searches on the all proteins of *B. rapa* using the previously used 49 Arabidopsis nsLtps as query sequences [17] (Fig. 1). With this approach, four additional putative *nsLtp* genes (Bra008112, Bra015966, Bra021299, and Bra040156) were picked up. Ultimately, we identified a total of 63 BrnsLtps in the whole genome of *B. rapa* (Table 1, Table S1, Table S2). Furthermore, the MM and theoretical pI (isoelectric point) of each BrnsLtp were calculated and summarized in Table 1. The three-dimensional structures of all putative BrnsLtps were also predicted and showed in Figure S1.

**Table 1:** Putative *nsLtp* genes identified in the genome of *B. rapa*.

Name	BRAD-locus	Chromosome Location	Strand	Intron (bp)	ECM^a^	SP^b^(AA^c^)	MP^d^(AA)	MP(MM^e^)	MP(pI^f^)
Type I									
*BrnsLtpI.1*	Bra000068	ChrA03: 9142369, 9142755	+	none	C-X_9_-C-X_13_-CC-X***_19_***-CXC-X_21_-C-X_13_-C	25	103	10723.34	10.26
*BrnsLtpI.2*	Bra001345	ChrA03: 15944546, 15944887	+	none	C-X_9_-C-X_14_-CC-X***_19_***-CXC-X_24_-C-X_13_-C	20	93	9651.88	10.28
BrnsLtpI.3*	Bra005098	ChrA05: 3409556, 3410188	−	228	C-X_9_-C-X_13_-CC-X***_19_***-CXC-X_23_-C-X_13_-C	18	116	11898.93	11.65
BrnsLtpI.4*	Bra005099	ChrA05: 3413125, 3413749	−	268	C-X_9_-C-X_14_-CC-X***_19_***-CXC-X_22_-C-X_13_-C	25	93	9414.92	11.08
*BrnsLtpI.5*	Bra006721	ChrA03: 4792207, 4792704	−	150	C-X_9_-C-X_13_-CC-X***_19_***-CXC-X_22_-C-X_13_-C	23	92	9175.57	9.53
*BrnsLtpI.6*	Bra006736	ChrA03: 4845130, 4845623	+	116	C-X_9_-C-X_13_-CC-X***_19_***-CXC-X_22_-C-X_13_-C	22	103	11229.85	7.31
*BrnsLtpI.7*	Bra012848	ChrA03: 21927444, 21928521	−	718	C-X_9_-C-X_13_-CC-X***_19_***-CXC-X_22_-C-X_13_-C	24	95	9922.37	8.05
BrnsLtpI.8*	Bra017112	ChrA04: 16558054, 16558647	+	237	C-X_9_-C-X_13_-CC-X***_19_***-CXC-X_23_-C-X_13_-C	25	93	9253.68	11.59
BrnsLtpI.9*	Bra017113	ChrA04: 16555397, 16556028	+	278	C-X_9_-C-X_13_-CC-X***_19_***-CXC-X_22_-C-X_13_-C	25	92	9430.85	11.73
BrnsLtpI.10*	Bra020322	ChrA02: 6218834, 6219334	−	162	C-X_9_-C-X_13_-CC-X***_19_***-CXC-X_19_-C-X_13_-C	23	89	8936.34	9.78
BrnsLtpI.11*	Bra020323	ChrA02: 6223820, 6224155	+	none	C-X_9_-C-X_14_-CC-X***_19_***-CXC-X_19_-C-X_13_-C	23	88	8849.35	10.00
*BrnsLtpI.12*	Bra024983	ChrA06: 24518522, 24518875	+	none	C-X_8_-C-X_16_-CC-X***_19_***-CXC-X_24_-C-X_9_-C	22	95	10052.28	10.07
*BrnsLtpI.13*	Bra029172	ChrA03: 6690210, 6690542	+	none	C-X_9_-C-X_13_-CC-X***_19_***-CXC-X_19_-C-X_13_-C	23	87	8740.24	10.30
*BrnsLtpI.14*	Bra029719	ChrA05: 22525095, 22525430	+	none	C-X_9_-C-X_13_-CC-X***_19_***-CXC-X_24_-C-X_13_-C	19	92	9535.75	9.82
*BrnsLtpI.15*	Bra036788	ChrA09: 25812372, 25812722	−	none	C-X_9_-C-X_13_-CC-X***_19_***-CXC-X_23_-C-X_13_-C	25	91	9682.25	12.44
*BrnsLtpI.16*	Bra036789	ChrA09: 25802085, 25802778	−	328	C-X_9_-C-X_14_-CC-X***_19_***-CXC-X_23_-C-X_13_-C	24	97	10473.18	8.28
*BrnsLtpI.17*	Bra037222	ChrA09: 4927969, 4928711	+	392	C-X_9_-C-X_14_-CC-X***_19_***-CXC-X_21_-C-X_13_-C	22	94	9413.56	4.52
BrnsLtpI.18*	Bra038907	ChrA01: 12636983, 12637614	+	272	C-X_9_-C-X_13_-CC-X***_19_***-CXC-X_22_-C-X_13_-C	24	95	9913.47	8.34
BrnsLtpI.19*	Bra038908	ChrA01: 12629898, 12630245	+	none	C-X_9_-C-X_13_-CC-X***_19_***-CXC-X_23_-C-X_13_-C	24	91	9693.23	12.40
**Type II**									
*BrnsLtpII.1*	Bra008112	ChrA02: 13195464, 13195760	+	none	C-X*_7_*-C-X_13_-CC-X_8_-CXC-X_23_-C-X_6_-C	30	68	7645.89	9.26
*BrnsLtpII.2*	Bra008375	ChrA02: 15167775, 15168068	+	none	C-X*_7_*-C-X_13_-CC-X_8_-CXC-X_23_-C-X_6_-C	18	79	8467.68	4.89
*BrnsLtpII.3*	Bra014154	ChrA08: 2994554, 2994847	−	none	C-X*_7_*-C-X_13_-CC-X_8_-CXC-X_23_-C-X_6_-C	29	68	7103.21	10.12
*BrnsLtpII.4*	Bra015966	ChrA07: 20026923, 20027216	+	none	C-X*_7_*-C-X_13_-CC-X_8_-CXC-X_23_-C-X_5_-C	30	67	7626.94	9.58
*BrnsLtpII.5*	Bra018687	ChrA06: 2603650, 2603949	−	none	C-X*_7_*-C-X_13_-CC-X_8_-CXC-X_23_-C-X_6_-C	24	75	8036.32	8.22
*BrnsLtpII.6*	Bra021299	ChrA01: 22700481, 22700774	+	none	C-X*_7_*-C-X_13_-CC-X_8_-CXC-X_23_-C-X_6_-C	29	68	7464.86	12.14
*BrnsLtpII.7*	Bra022308	ChrA05: 18509672, 18509962	−	none	C-X*_7_*-C-X_13_-CC-X_8_-CXC-X_23_-C-X_6_-C	28	68	7411.84	12.02
*BrnsLtpII.8*	Bra025378	ChrA06: 21876530, 21876814	+	none	C-X*_7_*-C-X_14_-CC-X_8_-CXC-X_25_-C-X_6_-C	23	71	7607.87	5.43
BrnsLtpII.9*	Bra027111	ChrA09: 8544965, 8545258	−	none	C-X*_7_*-C-X_13_-CC-X_8_-CXC-X_21_-C-X_6_-C	24	73	7856.14	5.06
BrnsLtpII.10*	Bra027114	ChrA09: 8562465, 8562758	−	none	C-X_7_-C-X_13_-CC-X_8_-CXC-X_21_-C-X_6_-C	24	73	7972.22	4.86
*BrnsLtpII.11*	Bra028162	ChrA04: 6295941, 6296249	+	none	C-X*_7_*-C-X_13_-CC-X_8_-CXC-X_23_-C-X_6_-C	24	78	8399.71	4.81
*BrnsLtpII.12*	Bra030699	ChrA08: 20754782, 20755075	+	none	C-X*_7_*-C-X_13_-CC-X_8_-CXC-X_23_-C-X_6_-C	24	73	7913.12	4.64
*BrnsLtpII.13*	Bra032265	ChrA05: 12176382, 12176675	−	none	C-X*_7_*-C-X_13_-CC-X_8_-CXC-X_23_-C-X_6_-C	29	68	7233.42	10.38
*BrnsLtpII.14*	Bra033084	ChrA02: 21491213, 21491515	+	none	C-X*_7_*-C-X_13_-CC-X_8_-CXC-X_23_-C-X_6_-C	26	74	7845.08	6.75
*BrnsLtpII.15*	Bra040627	ChrA02: 9924592, 9924879	+	none	C-X*_7_*-C-X_13_-CC-X_8_-CXC-X_23_-C-X_6_-C	21	74	7663.76	8.34
**Type III**									
*BrnsLtpIII.1*	Bra009282	ChrA10: 15963803, 15964270	−	177	C-X_9_-C-X_16_-CC-X_9_-CXC-X*_12_*-C-X_6_-C	29	67	6969.07	4.43
*BrnsLtpIII.2*	Bra028294	ChrA01: 18866657, 18866944	−	none	C-X_9_-C-X_16_-CC-X_9_-CXC-X*_12_*-C-X_6_-C	31	64	6730.60	4.90
*BrnsLtpIII.3*	Bra029135	ChrA03: 6509041, 6509295	−	none	C-X_9_-C-X_16_-CC-X_9_-CXC-X*_12_*-C-X_6_-C	20	64	6729.72	6.78
**Type IV**									
BrnsLtpIV.1*	Bra002906	ChrA10: 6849704, 6850036	+	none	C-X_9_-C-X_15_-CC-X_9_-CXC-X_22_-C-X_8_-C	23	87	9362.02	9.46
BrnsLtpIV.2*	Bra002907	ChrA10: 6843872, 6844180	+	none	C-X_9_-C-X_15_-CC-X_9_-CXC-X_19_-C-X_7_-C	28	74	7850.28	10.71
*BrnsLtpIV.3*	Bra002914	ChrA10: 6777869, 6778177	−	none	C-X_9_-C-X_15_-CC-X_9_-CXC-X_19_-C-X_7_-C	28	74	7774.18	10.10
*BrnsLtpIV.4*	Bra020696	ChrA02: 23700424, 23700738	−	none	C-X_9_-C-X_15_-CC-X_9_-CXC-X_24_-C-X_7_-C	28	76	7992.20	4.70
*BrnsLtpIV.5*	Bra020839	ChrA08: 11767442, 11767795	+	none	C-X_10_-C-X_11_-CC-X_12_-CXC-X_23_-C-X_7_-C	25	92	9845.14	4.52
*BrnsLtpIV.6*	Bra022364	ChrA05: 18159874, 18160179	+	none	C-X_9_-C-X_19_-CC-X_9_-CXC-X_24_-C-X_6_-C	23	78	8313.64	9.02
*BrnsLtpIV.7*	Bra028980	ChrA03: 5697175, 5697474	−	none	C-X_9_-C-X_15_-CC-X_9_-CXC-X_19_-C-X_6_-C	26	73	7472.52	4.46
*BrnsLtpIV.8*	Bra035573	ChrA02: 7465564, 7465998	−	72	C-X_9_-C-X_15_-CC-X_9_-CXC-X_22_-C-X_8_-C	30	90	9604.48	9.78
**Type V**									
*BrnsLtpV.1*	Bra005153	ChrA05: 3743608, 3744053	−	98	C-X*_14_*-C-X_14_-CC-X_12_-CXC-X_24_-C-X_10_-C	23	92	9521.52	12.31
*BrnsLtpV.2*	Bra014853	ChrA04: 3607346, 3607959	+	224	C-X*_14_*-C-X_14_-CC-X_11_-CXC-X_24_-C-X_10_-C	23	106	11085.73	9.16
**Type VI**									
*BrnsLtpVI.1*	Bra011229	ChrA01: 3189969, 3190301	−	none	C-X_10_-C-X_16_-CC-X_9_-CXC-X_22_-C-X_9_-C	19	91	9647.42	10.20
BrnsLtpVI.2*	Bra034567	ChrA08: 12794464, 12794909	−	101	C-X_10_-C-X_17_-CC-X_9_-CXC-X_22_-C-X_9_-C	28	86	9277.77	9.89
BrnsLtpVI.3*	Bra034568	ChrA08: 12792882, 12793311	−	85	C-X_10_-C-X_17_-CC-X_9_-CXC-X_22_-C-X_9_-C	28	86	9285.70	9.82
BrnsLtpVI.4*	Bra034570	ChrA08: 12788663, 12789092	−	85	C-X_10_-C-X_17_-CC-X_9_-CXC-X_22_-C-X_9_-C	28	86	9285.70	9.82
**Type VIII**									
*BrnsLtpVIII.1*	Bra015984	ChrA07: 19946287, 19946673	−	none	C-X_6_-C-X_14_-CC-X_12_-CXC-X*_25_*-C-X_8_-C	22	106	11657.72	8.11
**Type IX**									
*BrnsLtpIX.1*	Bra001252	ChrA03: 15530671, 15530955	−	none	C-X*_13_*-C-X_15_-CC-X_9_-CXC-X_22_-C-X_6_-C	17	77	7975.40	6.93
*BrnsLtpIX.2*	Bra006901	ChrA09: 26056900, 26057265	−	none	C-X*_13_*-C-X_15_-CC-X_9_-CXC-X_22_-C-X_6_-C	24	97	10290.02	4.72
*BrnsLtpIX.3*	Bra012819	ChrA03: 22081654, 22082025	+	none	C-X*_13_*-C-X_15_-CC-X_9_-CXC-X_22_-C-X_6_-C	26	97	10396.21	4.72
**Type XI**									
*BrnsLtpXI.1*	Bra000287	ChrA03: 10350000, 10350350	+	none	C-X_9_-C-X_18_-CC-X***_13_***-CXC-X_24_-C-X_9_-C	29	87	9167.88	6.86
*BrnsLtpXI.2*	Bra018483	ChrA05: 8507982, 8508317	+	none	C-X_9_-C-X_20_-CC-X***_13_***-CXC-X_24_-C-X_9_-C	23	88	9021.55	4.35
*BrnsLtpXI.3*	Bra018544	ChrA05: 9107060, 9107431	−	none	C-X_9_-C-X_18_-CC-X***_13_***-CXC-X_25_-C-X_9_-C	26	97	10165.23	9.81
BrnsLtpXI.4*	Bra024980	ChrA06: 24509174, 24509509	+	none	C-X_8_-C-X_16_-CC-X***_13_***-CXC-X_24_-C-X_9_-C	22	89	9465.57	10.35
BrnsLtpXI.5*	Bra024981	ChrA06: 24511564, 24511899	+	none	C-X_8_-C-X_16_-CC-X***_13_***-CXC-X_24_-C-X_9_-C	22	89	9465.57	10.35
BrnsLtpXI.6*	Bra024982	ChrA06: 24516109, 24516444	+	none	C-X_8_-C-X_16_-CC-X***_13_***-CXC-X_24_-C-X_9_-C	22	89	9465.57	10.35
**nsLTPY**									
*BrnsLtpY.1*	Bra024207	ChrA03: 26879106, 26879879	+	242, 106	C-X_9_-C-X_14_-CC-X_30_-CXC-X_23_-C-X_13_-C	21	120	13391.43	5.33
*BrnsLtpY.2*	Bra040156	ChrA01: 4374192, 4374566	+	none	C-X_10_-C-X_14_-CC-X_16_-CXC-X_21_-C-X_13_-C	20	104	11873.64	7.04

a **ECM, eight-cysteine motif;** **^b^SP, signal peptide; ^c^AA, number of amino acids; ^d^MP, mature protein; ^e^MM, molecular mass in Dalton; ^f^pI, isoelectric point (cysteine residues were not taken into account in the pI caculation). A cluster of tandem duplication repeats was indicated by an asterisk after the gene names. The values in ECM allowing direct identification of the nsLtp type are indicated in bold italic.**

### Sequence analysis and classification of putative BrnsLtps

**Figure 2:**
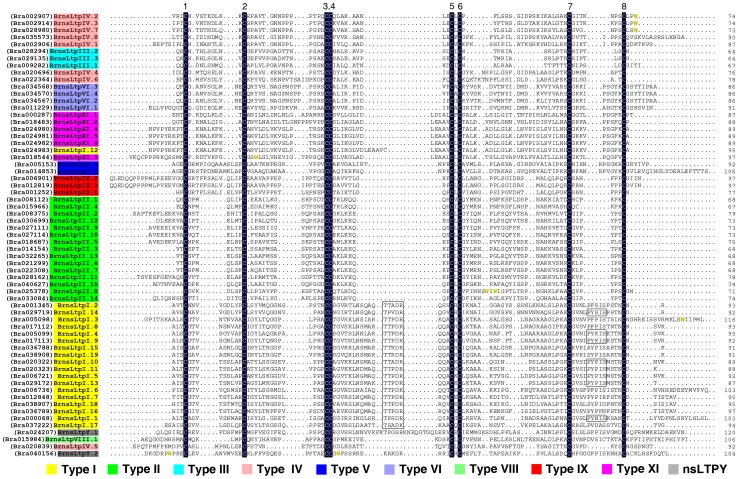
Multiple sequence alignment of the putative mature BrnsLtp proteins. The conserved cysteine residues are marked against a dark blue background. The names of different types of BrnsLtps are indicated with different color backgrounds. And the accession number of each gene was showed in the parentheses. Consensus residues Thr/Ser-X1-X2-Asp-Arg/Lys and Pro-Tyr-X-Ile-Ser are marked by rectangles. Tryptophan residues (W) are indicated with yellow circles.

Boutrot et al. [17] pointed out that the relationship between MM and nsLtp type was more complicated than previously thought and, as a consequence, was no longer considered to be a good criterion for classifying nsLtps. They also employed an alternative method to cluster 49 out of the 52 rice nsLtps and 45 out of the 49 Arabidopsis nsLtps into nine types based on sequence similarity [17]. Liu et al. [18] also used this method to classify 135 Solanaceae nsLtps into five types. Recently, Edstam et al. [51] even found that sequence similarity was not sufficient tool for a classification system if the sequences include genes from non-flowering plants. In this study, as for *B. rapa* is a flowering plant, we still applied the sequence similarity method to sort the BrnsLtps, and our results indicated that 61 out of the 63 BrnsLtps also could be divided into nine types (I, II, III, IV, V, VI, VIII, IX, and XI) mainly based on the identity matrix (data not shown) calculated from the multiple sequence alignments (Table 1, Fig. 2). Similarly, the majority (34 out of 63) of BrnsLtps belong to type I or type II nsLtps (Table 1, Fig. 2).

To clearly understand the sequence characteristics of BrnsLtps, we conducted a multiple sequence alignment using the deduced mature proteins from the 63 BrnsLtps (Fig. 2). The results showed that all the predicted mature BrnsLtps had a total number of amino acids that varied from 64 to 120 (Table 1 and Fig. 2). It was found that the eight Cys residues were highly conserved in all of the 63 BrnsLtps and that these residues may form four disulfide bonds to stabilize the tertiary structure of the hydrophobic cavity (Fig. 2; Figure S1). The proteins nsLtp1 (9 kDa) and nsLtp2 (7 kDa), however, have different disulfide bond patterns. The disulfide bond linkage of nsLtp1 at Cys_1_-Cys_6_ and Cys_5_-Cys_8_ differs from that of nsLtp2 at Cys_1_-Cys_5_ and Cys_6_-Cys_8_ [52]. Additionally, X is a hydrophilic residue in the CXC motif of nsLtp1; however, a hydrophobic residue was found at the X position in nsLtp2 [52]. These conserved hydrophobic or hydrophilic residues may play significant roles in the biological functions of nsLtps [53]. Our results showed that there were 11 different residues (Tyr, Leu, Phe, Val, Ile, Ala, Gly, Arg, Lys, Glu, and Gln) at the X position of the CXC motif in the 63 BrnsLtps (Fig. 2). Among of them, seven (Tyr, Leu, Phe, Val, Ile, Ala, and Gly) and four (Arg, Lys, Glu, and Gln) amino acids belong to the hydrophobic and hydrophilic residue groups, respectively. Leu is the most frequent residue that appears in the CXC motif of BrnsLtps, while Tyr and Gln appeared only once (Fig. 2). It is worth mentioning that the Leu residue is also the most frequent residue in the CXC of nsLTPs in Arabidopsis and rice, identified previously [17]. Douliez et al. [14] found that all plant nsLtp1 proteins had two highly conserved residues located in Thr/Ser-X1-X2-Asp-Arg/Lys and Pro-Tyr-X-Ile-Ser. Here, we also found that all type I nsLtps, save BrnsLtpI.12, possessed a Thr/Ser-X1-X2-Asp-Arg/Lys; however, only eight type I nsLtps had Pro-Tyr-X-Ile-Ser (Fig. 2 and Table S2). In other words, the Thr/Ser-X1-X2-Asp-Arg/Lys is more conserved and Pro-Tyr-X-Ile-Ser is more variable (Fig. 2). It was reported that no tryptophan residues were found in the sequence of nsLTP1 [14] and that the hydrophobic tunnel of *Ace*-AMP1 was probably interrupted by bulky, aromatic tryptophan and phenylalanine residues such that it could not bind and transfer lipids [54]. Our results indicated that seven BrnsLtps (BrnsLtpI.3, BrnsLtpII.8, BrnsLtpIV.2, BrnsLtpIV.3, BrnsLtpIV.7, BrnsLtpXI.3, and BrnsLtpY.2) possessed tryptophan residues (Fig. 2 and Table S2). Additionally, multiple alignments revealed a variable number of inter-cysteine amino acid residues (summarized in Table 2). Therefore, these BrnsLtp types can be identified according to the typical spacings for this motif. Our results showed that in the *B. rapa* genome, there are also no type VII nsLtps sequences, which contain 27 residues between the conserved Cys_6_ and Cys_7_, just as in Arabidopsis (Table 2). Additionally, we found a new type of BrnsLtps that contains 13 residues between Cys_4_ and Cys_5_, which we designated type “XI” to follow the Greek numeral “X”, which had already been used to identify another new type nsLtps found in Solanaceae [18]. Edstam et al. pointed out that novel nsLtps types may also have evolved during land plant evolution [51]. Although BrnsLtpI.12 was more similar to type XI than to type I BrnsLtps based on the sequence identity (Fig. 2), we consider this protein as type I nsLtps as for it contains 19 residues between Cys_4_ and Cys_5_. Furthermore, the mature proteins of BrnsLtpIV.5 and BrnsLtpIV.6 also shared low identities (less than 30%) with other type IV BrnsLtps (data not shown), we classified these two proteins into type IV nsLtps according to the typical spacings for ECM motifs and the following results of Bayesian estimation (Figure S2).

**Table 2:** Diversity of eight cysteine motifs in different types of BrnsLtps.

nsLTP type	ECM and number of flanking amino acid residues						
		1	2	3, 4	5 6	7	8
Type I	X_3,5,9,10_	C-X_9_	C-X_13,14,16_	C-C-X_19_	C-X-C-X_19,21-24_	C-X_9,13_	C-X_1,2,4,7,15,16,20_
Type II	X_0,2,7-9,12,13_	C-X_7_	C-X_13,14_	C-C-X_8_	C-X-C-X_21,23,25_	C-X_5,6_	C-X_0,2_
Type III	X_2_	C-X_9_	C-X_16_	C-C-X_9_	C-X-C-X_12_	C-X_6_	C-X_1,4_
Type IV	X_2,3,8,13_	C-X_9,10_	C-X_11,15,19_	C-C-X_9,12_	C-X-C-X_19,22-24_	C-X_6-8_	C-X_0,3,7,15_
Type V	X_3_	C-X_14_	C-X_14_	C-C-X_11,12_	C-X-C-X_24_	C-X_10_	C-X_6,21_
Type VI	X_2,9_	C-X_10_	C-X_16,17_	C-C-X_9_	C-X-C-X_22_	C-X_9_	C-X_7,8_
Type VIII	X_11_	C-X_6_	C-X_14_	C-C-X_12_	C-X-C-X_25_	C-X_8_	C-X_21_
Type IX	X_2,19_	C-X_13_	C-X_15_	C-C-X_9_	C-X-C-X_22_	C-X_6_	C-X_1,4_
Type XI	X_3,9,14_	C-X_8,9_	C-X_16,18,20_	C-C-X_13_	C-X-C-X_24,25_	C-X_9_	C-X_0–2_

The consensus motif of each nsLtp type was deduced from the analysis of the mature sequences of 63 putative BrnsLtps. The values allowing direct identification of the nsLtp type are underlined. Cysteine residues are indicated in bold italic. Character “X” represents any amino acid, and the Arabic numeral following “X” stands for the numbers of amino acid residues.

**Figure 3:**
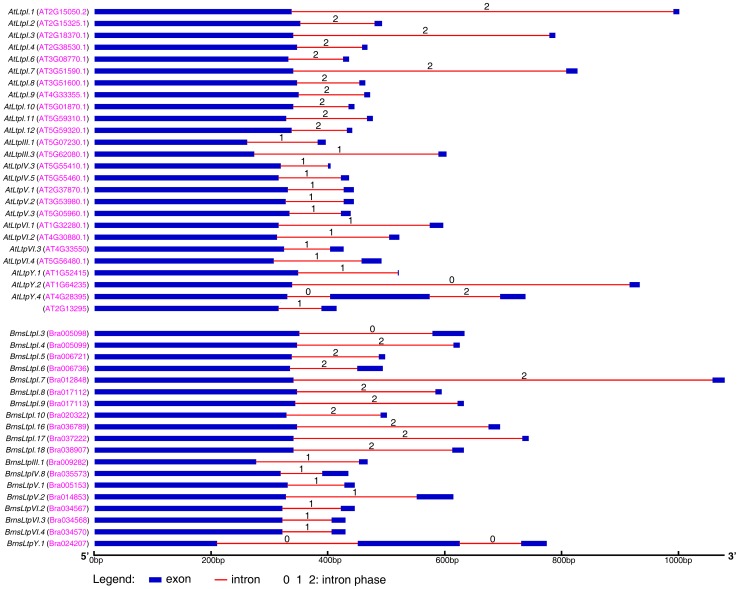
Gene structure of the *BrnsLtps* and *AtLtps*. Only those genes with introns (26 *AtLtps* and 19 *BrnsLtps*) are showed. The accession number of each gene is displayed in red font inside the parentheses. Intron phases are analysed based on the exon information. Phase 0 is designated introns between exons, phase 1 is designated introns between the first and the second nucleotide in a codon, and phase 2 is designated introns between the second and the third nucleotide in a codon [55].

Additionally, we examined the exon-intron organization of *BrnsLtp* family members. Our result indicated that only 19 *BrnsLtps* (six types) had introns, including 11 type I, one type III, one type IV, two type V, three type VI, and one nsLTPY *nsLtps* (Table 1 and Fig. 3). And all these 19 *BrnsLtps* save *BrnsLtpY.1* were predicted to be interrupted by a single intron positioned 7 to 59bp upstream of the stop codon (Table 1 and Fig. 3). Except for *BrnsLtpIV.8*, no introns were identified in the coding regions of type II, IV, VIII, IX, XI, and nsLTPY *BrnsLtp* genes (Table 1). A previous study showed that 25 Arabidopsis *Ltps* (11 type I, two type III, two type IV, three type V, four type VI, and three nsLTPY) possess introns [17]. Actually, Wang et al. [52] discovered several additional putative Ltp coding genes in Arabidopsis. Therefore, we also analysed the gene structure of these additional *AtLtps*, and our results demonstrated that only the protein encoded by AT2G13295 was predicted to have a single intron (Fig. 3). Exon shuffling may play an important role in the origin of both ancient and modern genes [55]. So the statistical analysis of intron phases (the position of introns within or between codons) is a good choice to evaluate the evolution between orthologous genes and paralogous genes. After comparison, we found that except for *BrnsLtpI.3* and nsLTPY *nsLtps*, the same type *nsLtps* had identical intron phase distribution (Fig. 3).

### Phylogenetic analysis of the putative AtnsLtps and BrnsLtps

**Figure 4:**
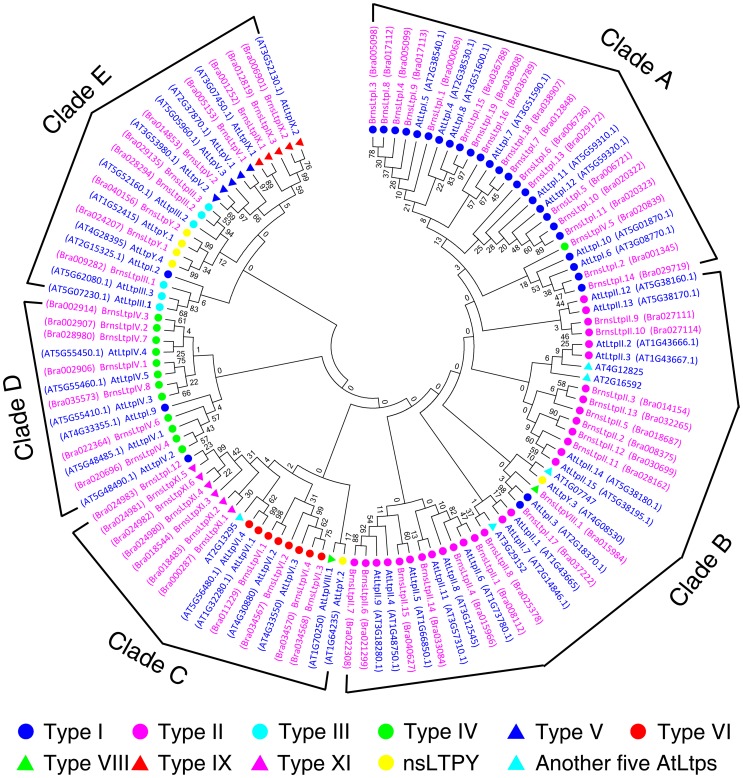
Phylogenetic tree of BrnsLtps. The amino acids of the ECMs were used for Neighbor-Joining phylogenetic tree construction using the MEGA 5.05 software. The ten types of nsLtps and another five AtnsLtps are indicated with circles or triangles of different colors. And the accession number of each gene is showed in the parentheses nearby the corresponding gene name.

In order to analyse the phylogenetic organization of the nsLtp families in Arabidopsis and *B. rapa*, we constructed a phylogenetic tree using Neighbor-Joining from the alignment of the amino acids of the ECMs. Boutrot et al. [17] previously pointed out that AtLtpI.1 and AtLtpII.10 were lacking Cys residues, and therefore, we discarded these two proteins from the 49 Arabidopsis nsLtps for the phylogenetic tree construction. Recently, Wang et al. [52] found additional *AtLtp* genes such as AT1G07747, AT1G52415, AT2G16592, AT2G13295, AT3G29152, and AT4G12825. Only four other AtLtps were discovered by Wang et al. [52] on account of the fact that AT1G52415 is the same as *AtLtpY.1*, which was identified previously by Boutrot et al. [17]. Thus, a total of 52 AtnsLtps and 63 BrnsLtps were used to conduct a protein-based phylogenetic analysis (Fig. 4). Our results showed that these 115 nsLtps can be divided into five clades (Fig. 4). There were 18, 17, 11, seven, and ten BrnsLtps in each clade from A–E, respectively (Fig. 4). Almost all type I BrnsLtps were grouped in clade A, all type II were grouped in clade B, and all type IV BrnsLtps were grouped in clade D. The C and E clades were each composed of proteins from two or four main types, respectively (Fig. 4). The phylogeny of BrnsLtps and AtLtps was also determined with Bayesian estimation using the MrBayes program (Figure S2). Similar to the Neighbor-Joining phylogenetic results, the Bayesian estimation also indicated that almost all the sequences belonging to the same type are grouped and constitute monophyletic groups, except for the type II nsLtps (Figure S2). Type II nsLtps are close in the tree but are not grouped in a single clade. This may be because several AtLtps (AtLtpII.1, AtLtpII.2, AtLtpII.3, AtLtpII.7, AtLtpII.11, AtLtpII.12, AtLtpII.13, AT1G07747, and AT4G12825) were more distantly related to other type II nsLtps (Figure S2). Furthermore, all type II BrnsLtps except for BrnsLtpII.5 appeared to be monophyletic (Figure S2). Previously, Boutrot et al. [17] pointed out that the general organization of the tree is consistent with the classification of nsLtps types except for type II nsLtps via phylogenetic analysis from the alignments of 45, 49, and 122 sequences of Arabidopsis, rice, and wheat nsLtps using maximum-likelihood inference. Amazingly, we also found that the nine type II *AtLtps* not grouped in a clade had no homologous genes in the three subgenomes of *B. rapa* (Table 3). It is an interesting question as to why these genes were missed during the triplication evolution process from *A. thaliana* to *B. rapa*. It was also worth noting that when the tree is built with only rice and wheat sequences, type II nsLtps seems to be monophyletic [17]. Does this mean that the monocotyledon plants discarded these genes during the evolutionary divergence between monocots and dicots?

**Table 3:** Identification of homologous *nsLtp* genes between *A. thaliana* and three subgenomes in *B. rapa*.

*A. thaliana*	CCB^a^	LF^b^	MF1^c^	MF2^d^	*A. thaliana*	CCB	LF	MF1	MF2
*AtLtpI.1*	/	/	/	/	*AtLtpV.1*	J	*BrnsLtpV.1*	/	/
*AtLtpI.2*	/	/	/	/	*AtLtpV.2*	N	/	BrnsLtpV.*2*	/
*AtLtpI.3*	H	/	/	*BrnsLtpI.17*	*AtLtpV.3*	R	*Bra009154*	/	/
*AtLtpI.4*	J – Tandem	*BrnsLtpI.3 BrnsLtpI.4 –* Tandem	*BrnsLtpI.8 BrnsLtpI.9 –* Tandem	*BrnsLtpI.1*	*AtLtpVI.1*	B	/	/	/
*AtLtpI.5*	J – Tandem	*BrnsLtpI.3 BrnsLtpI.4 –*Tandem	*BrnsLtpI.8 BrnsLtpI.9 –* Tandem	*BrnsLtpI.1*	*AtLtpVI.2*	U	*BrnsLtpVI.1*	/	Bra010269
*AtLtpI.6*	F	*BrnsLtpI.14*	/	*BrnsLtpI.2*	*AtLtpVI.3*	U	/	/	*BrnsLtpVI.2 BrnsLtpVI.3 BrnsLtpVI.4* *–* Tandem
*AtLtpI.7*	N – Tandem	*BrnsLtpI.15*	*BrnsLtpI.7*	*BrnsLtpI.18 BrnsLtpI.19* *–* Tandem	*AtLtpVI.4*	W	Bra002813	/	/
*AtLtpI.8*	N – Tandem	*BrnsLtpI.15*	*BrnsLtpI.7*	*BrnsLtpI.18 BrnsLtpI.19* *–* Tandem	*AtLtpVIII.1*	E	*/*	Bra007907 Bra007908 Bra007909 – Tandem	/
*AtLtpI.9*	U	/	/	/	*AtLtpIX.1*	F	Bra029649	/	*BrnsLtpIX.1*
*AtLtpI.10*	R	/	/	/	*AtLtpIX.2*	N	BrnsLtpIX.2	*BrnsLtpIX.3*	/
*AtLtpI.11*	W – Tandem	/	*BrnsLtpI.10 BrnsLtpI.11 *– Tandem	*BrnsLtpI.6*	*AtLtpY.1*	C	/	/	/
*AtLtpI.12*	W – Tandem	/	*BrnsLtpI.10 BrnsLtpI.11 –* Tandem	*BrnsLtpI.6*	*AtLtpY.2*	/	/	/	/
*AtLtpII.1*	C	/	/	/	*AtLtpY.3*	/	/	/	/
*AtLtpII.2*	C	/	//	/	*AtLtpY.4*	U	/	*BrnsLtpY.1*	/
*AtLtpII.3*	C	/	/	/	AT1G07747	A	/	/	/
*AtLtpII.4*	C	/	*BrnsLtpII.3*	*BrnsLtpII.13*	AT2G16592	H – Tandem	/	/	/
*AtLtpII.5*	E	/	*BrnsLtpII.15*	/	AT2G13295	/	/	/	/
*AtLtpII.6*	E	*BrnsLtpII.4*	*BrnsLtpII.1*	/	AT3G29152	L	*BrnsLtpII.8*	/	/
*AtLtpII.7*	/	/	/	/	AT4G12825	T	/	/	/
*AtLtpII.8*	F	/	/	/	/	/	/	/	*BrnsLtpI.5*
*AtLtpII.9*	F	*BrnsLtpII.7*	*BrnsLtpII.6*	*Bra001701*	AT5G46890	V – Tandem	*BrnsLtpXI.4 BrnsLtpXI.5* *BrnsLtpXI.6* *BrnsLtpI.12 –* Tandem	/	Bra017517 Bra017518 – Tandem
*AtLtpII.10*	/	/	/	/	/	/	/	/	*BrnsLtpI.13*
*AtLtpII.11*	N	/	/	/	/	/	*BrnsLtpI.16*	/	/
*AtLtpII.12*	S – Tandem	/	/	/	/	/	/	*BrnsLtpII.2*	/
*AtLtpII.13*	S – Tandem	/	/	/	/	/	*BrnsLtpII.5*	/	/
*AtLtpII.14*	S	/	/	/	/	/	BrnsLtpII.9 BrnsLtpII.10 – Tandem	/	/
*AtLtpII.15*	S – Tandem	*BrnsLtpII.11*	/	/	/	/	/	/	*BrnsLtpII.12*
*AtLtpIII.1*	R	*BrnsLtpIII.1*	/	/	/	/	/	*BrnsLtpII.14*	/
*AtLtpIII.2*	W	*BrnsLtpIII.2*	/	*BrnsLtpIII.3*	AT4G22666	U	/	/	*BrnsL tpIV.5* Bra020840 – Tandem
*AtLtpIII.3*	X	/	/	/	/	/	*BrnsLtpIV.6*	/	/
*AtLtpIV.1*	V – Tandem	/	*BrnsLtpIV.4*	/	/	/	*BrnsLtpVIII.1*	/	/
*AtLtpIV.2*	V – Tandem	/	*BrnsLtpIV.4*	/	/	/	/	/	*BrnsLtpXI.1*
*AtLtpIV.3*	W	*BrnsLtpIV.3*	/	/	AT4G12470	Tandem	Bra029456 Bra029458 – Tandem	Bra000775 Bra000777 Bra000778 – Tandem	Bra018481 *BrnsLtpXI.2* Bra018484– Tandem
*AtLtpIV.4*	W	*BrnsLtpIV.2*	*BrnsLtpIV.8 Bra035574 –* Tandem	*BrnsLtpIV.7* *Bra028981 –* Tandem	/	/	/	/	
*AtLtpIV.5*	W	*BrnsLtpIV.1*	/	/	/	/	*BrnsLtpY.2*	/	/

a **CCB, conserved collinear block; ^b^LF, the least fractionated blocks of B.** ***rapa*** **; ^c^MF1, the medium fractionated blocks of B.** ***rapa*** **; ^d^MF2, the most fractionated blocks of B.** ***rapa*** **.**

### Chromosomal localization and gene duplication

**Figure 5:**
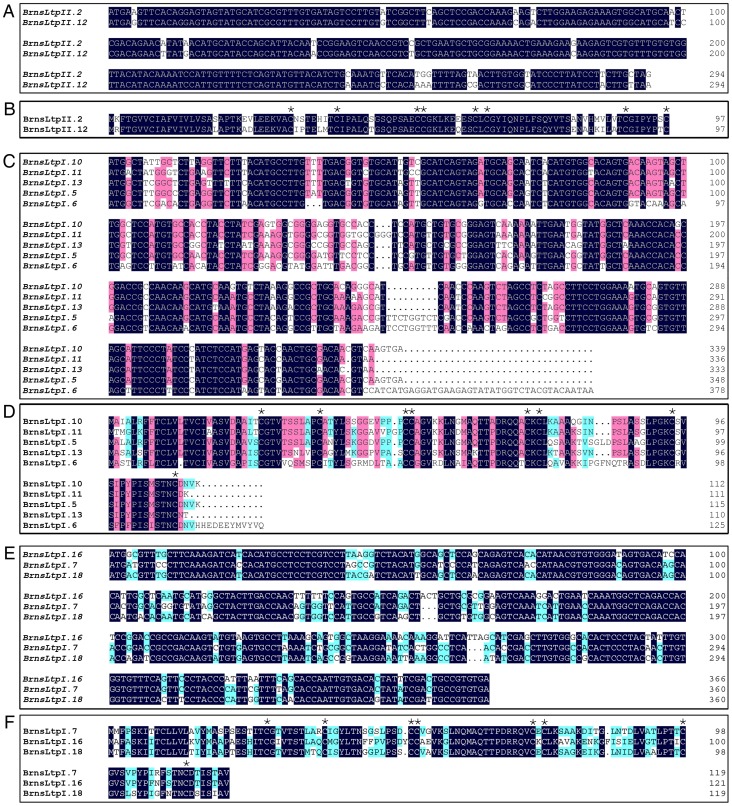
Genomic localization of the *BrnsLtp* genes on the chromosomes of *B. rapa*. Chromosome numbers are indicated above each chromosome. The number of *BrnsLtp* genes distributed on each *B. rapa* chromosome is indicated by an Arabic numeral in the bracket, which is under the relative chromosome number. And the accession number of each gene was showed in the parentheses underneath the corresponding gene name. The *BrnsLtp* genes present on duplicated chromosomal segments are connected by blue lines between the two relevant chromosomes. Tandem duplicated genes are marked on a yellow background. The conserved collinear blocks on each chromosome are labeled A to X and are color-coded according to inferred ancestral chromosomes following an established convention.

The approximate position of each *BrnsLtp* on the physical map of the *B. rapa* genome was marked based on information obtained from the *Brassica* Database (BRAD). The 63 *BrnsLtps* were randomly distributed across ten chromosomes of *B. rapa*, from A01 to A10 (Fig. 5). Twelve genes were located on chromosome A03, nine genes on chromosome A05, eight genes on chromosome A02, six genes each on chromosomes A01, A06, A08, and A09, four genes each on chromosome A04 and A10, and two genes on chromosome A07 (Fig. 5). Additionally, comparison of the *nsLtp* genes those are homologous between Arabidopsis and the three subgenomes of *B. rapa* revealed that 27 *BrnsLtp* genes are located on the least fractionated blocks (LF), 16 *BrnsLtps* are on the medium fractionated blocks (MF1), and 20 *BrnsLtps* are on the most fractionated blocks (MF2) (Table 3).

**Figure 6:**
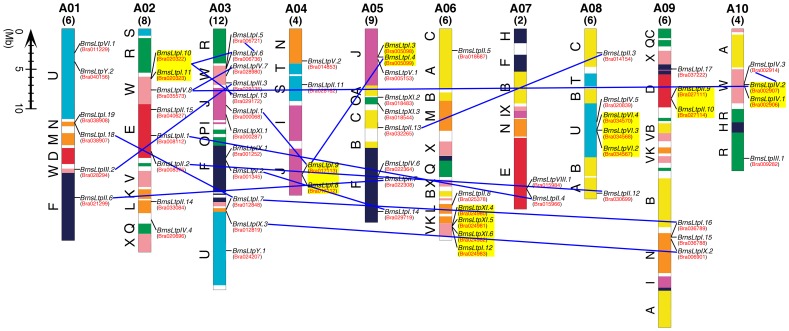
Alignments of the coding sequences and the deduced protein sequences of selected *BrnsLtp* genes in *B. rapa*. (A) Alignment of the *BrnsLtpII.2* and *BrnsLtpII.12* coding sequences. (B) Alignment of the BrnsLtpII.2 and BrnsLtpII.12 protein sequences. (C) Alignment of the *BrnsLtpI.5/6/10/11/13* coding sequences. (D) Alignment of the BrnsLtpI.5/6/10/11/13 protein sequences. Nucleic acid bases or amino acid residues in positions conserved in 100, 75, and 50% of all sequences are shaded in dark blue, purple, and light blue, respectively. The asterisks in (B) and (D) indicate the cysteine residues of the deduced protein backbones.

Wang et al. [56] confirmed that the genome of *B. rapa* is almost a complete triplication of the genome of the small cruciferous model plant *A. thaliana*. Cannon et al. [57] pointed out both segmental and tandem gene duplications play important roles in the expansion and evolution of gene families in plant genomes. In this study, we investigated the influence of duplications on the expansion of the *BrnsLtp* gene family during evolution. Our results also confirmed that segmental duplication, as well as tandem duplication, contributed to the expansion of this gene family in *B. rapa* (Fig. 5 and Table 3). Except for chromosome A06, the other nine chromosomes exhibited segmental *nsLtp* gene duplications (Fig. 5). Commonly, genes separated by ≤5 genes are considered to be tandem duplicates. According to this principle, we found that seven chromosomes, except for chromosomes A01, A03, and A07, had tandem *nsLtp* gene duplications (Fig. 5 and Table 3). These entire 17 tandem duplicated genes belong to five nsLtp types (I, II, IV, VI, and XI) (Fig. 5 and Table 3). A previous study showed that 18 out of the 49 Arabidopsis *nsLtp* genes belonging to three types (I, II, and IV) are tandem duplication repeats [17]. Although the results in Table 3 showed that *BrnsLtpIV.1* and *BrnsLtpIV.2* were not tandem duplicated, we inferred that there was an existing duplication between these two genes based on three reasons: 1. the genes homologous to *BrnsLtpIV.1* and *BrnsLtpIV.2* are *AtLtpIV.5* (AT5G55460.1) and *AtLtpIV.4* (AT5G55450.1), respectively, and these two Arabidopsis *nsLtp* genes are duplication repeats [17]; 2. The BRAD-loci of *BrnsLtpIV.1* and *BrnsLtpIV.2* are Bra002906 and Bra002907 (Table 1) respectively, which is adjacent; and 3. *BrnsLtpIV.1*, *BrnsLtpIV.2*, and *BrnsLtpIV.3* share 80.34% similarity in their coding sequences (data not shown) (Figure S3). As for *BrnsLtpIV.3* (Bra002914) and *BrnsLtpIV.2* (Bra002907), which are separated by six other gene loci, we consider *BrnsLtpIV.3* to be a segmental duplication of *BrnsLtpIV.1* and *BrnsLtpIV.2* rather than a tandem duplication (Fig. 5). In spite of the homologous *nsLtp* genes determined from publicly available data that provided useful information (Table 3), we found some minor defects in BRAD after careful analysis. For example, *BrnsLtpII.2* may be homologous to *BrnsLtpII.12* because their coding sequences and their deduced protein sequences are up to 92.18% and 83.51% identical, respectively (Fig. 6). With the exception of *BrnsLtpI.6/10/11*, *BrnsLtpI.5* and *BrnsLtpI.13* may also be homologous to *AtLtpI.11/12* (Fig. 6). Though the results of Table 3 show that *BrnsLtpI.18* and *BrnsLtpI.19* share a tandem duplication between them, the identity of their coding sequences was low, only 50.96%. This low identity (51.91%) was also observed between *BrnsLtpI.15* and *BrnsLtpI.7* (data not shown). However, *BrnsLtpI.16* has high sequence similarity with *BrnsLtpI.7*/*18* not only in the coding sequences (89.71%) but also in the protein sequences (82.92%) (Fig. 6). Therefore, *BrnsLtpI.7*/*18* might be homologous to *BrnsLtp16* rather than to *BrnsLtpI.15/19* (Fig. 6). Furthermore, comparison of the homologous *nsLtp* genes in Arabidopsis and the three *B. rapa* subgenomes revealed that the conserved collinear block of each *BrnsLtp* is highly identical to that in Arabidopsis (Fig. 5 and Table 3).

### Expression analysis of *BrnsLtp* genes

The *B. rapa* expressed sequence tags (ESTs) in GenBank are a valuable resource for gene discovery, genome annotation, and analysis of gene expression in this species. To investigate the expression patterns of *BrnsLtps*, we first took full advantage of the *B. rapa* ESTs data to analyse EST counts from six different organs (buds, flowers, leaves, roots, and siliques) (Table 4, Table S3). Our results showed that there were no EST data for eight *BrnsLtps*, EST data for 12 BrnsLtps were available only for unspecified tissue, and the most abundant *nsLtp* expression was attributed to type I *BrnsLtps* (Table 4, Table S3). Wang et al. [52] also found that rice type I nsLtps had the most ESTs counts after analysis of the public rice microarray data. Furthermore, the ESTs of 15 *BrnsLtps* seems to be exclusively detected in a single organ (Table 4). These genes included three genes present in the bud with UniGene numbers Bra.140 (*BrnsLtpIII.2/3*) and Bra.20831 (*BrnsLtpIX.2*); one flower gene with UniGene number Bra.25295 (*BransLtpIX.1*); seven leaf genes with UniGene numbers Bra.18420 (*BrnsLtpI.12* and *BrnsLtpXI.4/5/6*), Bra.2602 (*BrnsLtpIV.2/3*), and Bra.30903 (*BrnsLtpXI.2*); three root genes with UniGene numbers Bra.17303 (*BrnsLtpI.17*), Bra.5092 (*BrnsLtpIV.6*), and Bra.26893 (*BrnsLtpV.1*); and one silique gene with the UniGene number Bra.12938 (*BrnsLtpIV.7*) (Table 4). If we consider the bud and flower as a whole (inflorescence), then the ESTs of six other genes appeared to be specifically detected in this reproductive tissue, including Bra.1371 (*BrnsLtpI.16/18*), Bra.503 (*BrnsLtpII.15*), Bra.21209 (*BrnsLtpIII.1*), Bra.21558 (*BrnsLtpIX.3*), and Bra.389 (*BrnsLtpY.1*) (Table 4). In total, 21 out of 63 *BrnsLtps* were likely to show specific expression patterns. Moreover, no ESTs were found specifically in the stem, and only four genes (*BrnsLtpI.1/3/4/8*) had ESTs in the stem in the current databases (Table 4).

**Table 4:** Expression analysis of *BrnsLtp* genes by UniGene (Transcripts Per Million, TPM).

Name	UN^a^	B^b^	F^c^	L^d^	R^e^	S^f^	Si^g^	WP^h^	UT^i^	Other	Name	UN	B	F	L	R	S	Si	WP	UT	Other
*BrnsLtpI.1*	Bra.119	2180	856	1724	349	949	4151	√	√	√	*BrnsLtpII.14*	Bra.11101	–	–	–	–	–	–	–	√	–
*BrnsLtpI.2*	Bra.1471	363	0	172	279	0	125	√	√	√	*BrnsLtpII.15*	Bra.503	1635	2336	0	0	0	0	–	–	–
*BrnsLtpI.3*	Bra.71	2180	1012	57	0	474	4403	–	–	√	*BrnsLtpIII.1*	Bra.21209	1453	233	0	0	0	0	–	–	–
*BrnsLtpI.4*	Bra.119	2180	856	1724	349	949	4151	√	√	√	*BrnsLtpIII.2*	Bra.140	302	0	0	0	0	0	–	–	–
*BrnsLtpI.5*	Bra.13066	–	–	–	–	–	–	–	√	–	*BrnsLtpIII.3*	Bra.140	302	0	0	0	0	0	–	–	–
*BrnsLtpI.6*	Bra.13066	–	–	–	–	–	–	–	√	–	*BrnsLtpIV.1*	–	–	–	–	–	–	–	–	–	–
*BrnsLtpI.7*	Bra.8616	2604	1869	0	0	0	0	√	√	–	*BrnsLtpIV.2*	Bra.2602	0	0	114	0	0	0	√	–	√
*BrnsLtpI.8*	Bra.71	2180	1012	57	0	474	4403	–	–	√	*BrnsLtpIV.3*	Bra.2602	0	0	114	0	0	0	√	–	√
*BrnsLtpI.9*	Bra.1589	0	233	574	139	0	880	√	√	√	*BrnsLtpIV.4*	Bra.3442	0	311	229	0	0	251	–	–	–
*BrnsLtpI.10*	Bra.9345	0	0	57	0	0	3270	√	–	–	*BrnsLtpIV.5*	–	–	–	–	–	–	–	–	–	–
*BrnsLtpI.11*	Bra.293	121	311	57	0	0	2390	–	–	√	*BrnsLtpIV.6*	Bra.5092	0	0	0	209	0	0	–	–	–
*BrnsLtpI.12*	Bra.18420	0	0	57	0	0	0	–	–	√	*BrnsLtpIV.7*	Bra.12938	0	0	0	0	0	125	–	√	–
*BrnsLtpI.13*	Bra.10478	0	0	57	0	0	1509	–	√	√	*BrnsLtpIV.8*	Bra.10653	–	–	–	–	–	–	–	√	–
*BrnsLtpI.14*	Bra.148	121	4985	57	0	0	1635	–	√	–	*BrnsLtpV.1*	Bra.26893	0	0	0	139	0	0	–	–	–
*BrnsLtpI.15*	Bra.3744	0	0	114	69	0	0	√	√	√	*BrnsLtpV.2*	Bra.3434	0	0	114	139	0	125	–	–	–
*BrnsLtpI.16*	Bra.1371	5390	4907	0	0	0	0	–	√	–	*BrnsLtpVI.1*	Bra.7258	–	–	–	–	–	–	–	√	–
*BrnsLtpI.17*	Bra.17303	0	0	0	698	0	0	–	–	√	*BrnsLtpVI.2*	–	–	–	–	–	–	–	–	–	–
*BrnsLtpI.18*	Bra.1371	5390	4907	0	0	0	0	–	√	–	*BrnsLtpVI.3*	–	–	–	–	–	–	–	–	–	–
*BrnsLtpI.19*	Bra.9008	0	389	57	0	0	0	–	–	–	*BrnsLtpVI.4*	–	–	–	–	–	–	–	–	–	–
*BrnsLtpII.1*	–	–	–	–	–	–	–	–	–	–	*BrnsLtpVIII.1*	–	–	–	–	–	–	–	–	–	–
*BrnsLtpII.2*	Bra.11434	–	–	–	–	–	–	–	√	–	*BrnsLtpIX.1*	Bra.25295	0	233	0	0	0	0	–	–	–
*BrnsLtpII.3*	Bra.17924	0	311	0	69	0	0	√	–	√	*BrnsLtpIX.2*	Bra.20831	60	0	0	0	0	0	–	–	–
*BrnsLtpII.4*	–	–	–	–	–	–	–	–	–	–	*BrnsLtpIX.3*	Bra.21558	121	77	0	0	0	0	√	–	–
*BrnsLtpII.5*	Bra.11200	–	–	–	–	–	–	–	√	–	*BrnsLtpXI.1*	Bra.31801	–	–	–	–	–	–	–	–	–
*BrnsLtpII.6*	Bra.4920	0	311	57	628	0	251	√	–	√	*BrnsLtpXI.2*	Bra.30903	0	0	57	0	0	0	–	–	–
*BrnsLtpII.7*	Bra.4920	0	311	57	628	0	251	√	–	√	*BrnsLtpXI.3*	Bra.10370	–	–	√	–	–	–	–	–	√
*BrnsLtpII.8*	–	–	–	–	–	–	–	–	–	–	*BrnsLtpXI.4*	Bra.18420	0	0	57	0	0	0	–	–	√
*BrnsLtpII.9*	Bra.11200	–	–	–	–	–	–	–	√	–	*BrnsLtpXI.5*	Bra.18420	0	0	57	0	0	0	–	–	√
*BrnsLtpII.10*	Bra.11200	–	–	–	–	–	–	–	√	–	*BrnsLtpXI.6*	Bra.18420	0	0	57	0	0	0	–	–	√
*BrnsLtpII.11*	Bra.6760	–	–	–	–	–	–	–	√	–	*BrnsLtpY.1*	Bra.389	181	77	0	0	0	0	–	–	–
*BrnsLtpII.12*	Bra.11434	–	–	–	–	–	–	–	√	–	*BrnsLtpY.2*	Bra.7621	–	–	–	–	–	–	–	√	–
*BrnsLtpII.13*	Bra.17924	0	311	0	69	0	0	√	–	√											

a **UN, unigene number; ^b^B, bud; ^c^F, flower; ^d^L, leaf; ^e^R, root; ^f^S, seed; ^g^Si, silique; ^h^WP, whole plant; ^i^UT, unspecified tissue; “√” and “–” represent “exist” and “not exist”, respectively. Underlined indicated specific expression.**

**Figure 7:**
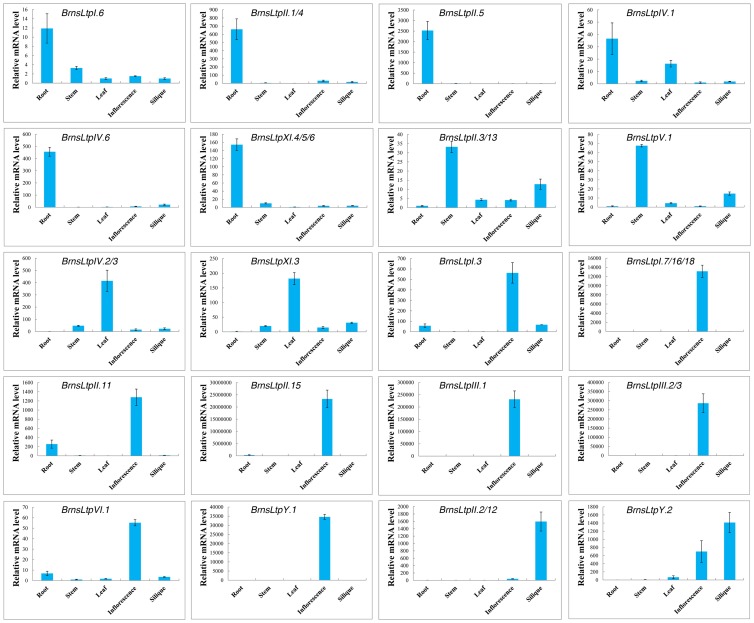
Quantitative RT-PCR analysis for selected *BrnsLtp* genes in tissues and organs of *B. rapa* with specific expression patterns.

**Figure 8:**
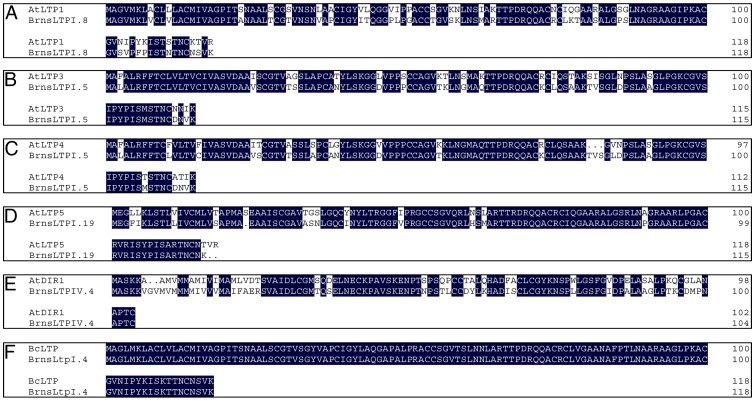
Alignments of the precursor protein sequences of nsLtps with known functions and their homologous in *B. rapa*.

To validate the expression patterns of *BrnsLtp* genes indicated by the UniGene data, we employed quantitative RT-PCR analysis for five different tissues of *B. rapa*, including the roots, stems, leaves, inflorescences, and siliques (Fig. 7, Figure S4, Table S4). Our results showed that the expression profiles of the ten genes were in agreement with the EST data. For example, seven genes (*BrnsLtpI.16/18*, *BrnsLtpIII.1*, *BrnsLtpIII.2/3*, *BrnsLtpII.15*, and *BrnsLtpY.1*) were highly expressed in the inflorescence, while *BrnsLtpIV.2/3* and *BrnsLtpIV.6* were strongly expressed in the leaf and root, respectively (Fig. 7). As for the coding sequence of *BrnsLtpI.7*, it was highly identical to that of *BrnsLtpI.16/18* (Figure S3); this similarity meant that a common primer was shared by these three genes for quantitative RT-PCR analysis. Although the relevant UniGene number of *BrnsLtpI.7* was different from that of *BrnsLtpI.16/18*, their ESTs seems to be only detected in the inflorescence (Table 4). Therefore, we considered that *BrnsLtpI.7* and *BrnsLtpI.16/18* were specifically expressed in the inflorescence, which consistent with the results shown in Figure 7. The expression patterns of the other five *BrnsLtp* genes (including *BrnsLtpI.12*, *BrnsLtpV.1*, and *BrnsLtpXI.4/5/6*) were not in accordance with the results obtained from digital expression analysis (Table 4, Fig. 7, Figure S4). For example, *BrnsLtpV.1* was highly expressed in the stem rather than in the root, while *BrnsLtpXI.4/5/6* was highly expressed in the root rather than in the leaf (Table 4, Fig. 7). Additionally, another 14 *BrnsLtp* genes were found with specific expression patterns, such as five genes (*BrnsLtpI.6*, *BrnsLtpII.1/4*, *BrnsLtpII.5*, and *BrnsLtpIV.1*) that were expressed in the root, two genes (*BrnsLtpII.3/13*) in the stem, one gene (*BrnsLtpXI.3*) in the leaf, three genes (*BrnsLtpI.3*, *BrnsLtpII.11*, and *BrnsLtpVI.1*) in the inflorescence, and three genes (*BrnsLtpII.2/12* and *BrnsLtpY.2*) in the silique (Fig. 7). In sum, 29 out of 63 *BrnsLtps* were found with specific expression patterns, and 11 *BrnsLtps* were specifically expressed in the inflorescence (Fig. 7). The *cis*-acting regulatory elements (CREs) located in the promoter sequence are considered to regulate the gene expression level. Therefore, we investigated the CREs of the 11 *BrnsLtps* with inflorescence-specific expression patterns. And our results indicated that a certain number of POLLEN1LELAT52 [58] and GTGANTG10 [59] CREs that belong to the late pollen genes are present in the promoters of all these 11 *BrnsLtps* (Table 5). Additionally, we found that some BrnsLtps might be homologous to certain nsLtps of other species or varieties with known functions, such as BrnsLtpI.8 and AtLTP1 (AtLtpI.5) (72.88% identity), BrnsLtpI.5 and AtLTP3/4 (AtLtpI.12/11) (82.61% and 80.87% identities, respectively), BrnsLtpI.19 and AtLTP5 (AtLtpI.8) (86.44% identity), and BrnsLtpIV.4 and AtDIR1 (AtLtpIV.1) (66.35% identity); BrnsLtpI.4 was identical to BcLTP (Fig. 8, Table 6). However, due to difficulties in designing specific primers (Figure S3), we did not determine the expression profiles of *BrnsLtpI.4/5/8/19* by quantitative RT-PCR. A previous study indicated that *AtDIR1* is expressed in seedlings, flowers and leaves [60]. A recent study also showed that long distance movement of DIR1 and the role of DIR1-like during systemic acquired resistance in Arabidopsis [61]. Here, our results showed that *BrnsLtpIV.4* was highly expressed in the root, inflorescence, and silique and less so in the stem and leaf (Figure S4). Though *BrnsLtpIV.4* was homologous to *AtDIR1* (*AtLtpIV.1*) (Table 3), their expression patterns were different. This may be due to the differences in regulatory elements in promoter regions via deletion and/or acquisition of regulatory sequences during evolution. In addition, the expression profile of *BrnsLtpII.14* was not presented here because the result was not ideal.

**Table 5:** Analysis of the number of plant CREs belonging to pollen genes in the promoters of selected *BrnsLtps*.

Gene Name	BRAD-Locus	Promoter Length (bp)	Plant Cis-acting Regulatory Elements	
			POLLEN1LELAT52	GTGANTG10
*BrnsLtpI.3*	Bra005098	1927^a^	14	7
*BrnsLtpI.7*	Bra012848	896^a^	4	3
*BrnsLtpI.16*	Bra036789	2000^b^	21	18
*BrnsLtpI.18*	Bra038907	2000^b^	10	5
*BrnsLtpII.11*	Bra028162	2000^b^	12	11
*BrnsLtpII.15*	Bra040627	2000^b^	14	10
*BrnsLtpIII.1*	Bra009282	1348^c^	8	14
*BrnsLtpIII.2*	Bra028294	482^a^	2	5
*BrnsLtpIII.3*	Bra029135	1456^c^	4	13
*BrnsLtpVI.1*	Bra011229	2000^b^	9	11
*BrnsLtpY.1*	Bra024207	2000^b^	4	9

**a: the promoter sequence is interrupted by many uncertain nucleotides marked with “N”; b: the length of intergenic region is more than 2000bp; c: the length of intergenic region is less than 2000bp.**

**Table 6:** The summary of the *nsLtp* genes with known functions.

Gene name (species or varieties)	Accession number	Known functions	Original reference
*LTP1* (*Arabidopsis thaliana*)	At2g38540	Calmodulin-binding protein and cell differentiation	[95], [26]
*LTP3* (*Arabidopsis thaliana*)	At5g59320	Freezing and drought stress	[43]
*LTP4* (*Arabidopsis thaliana*)	At5g59310	Pathogen defence	[96]
*LTP5* (*Arabidopsis thaliana*)	At3g51600	Pollen tube tip growth and fertilization	[32]
*DIR1* (*Arabidopsis thaliana*)	At5g48485	Systemic resistance signalling	[45]
*LTP2* (*Hordeum vulgare* cv. Bomi)	AAA03283.1	Antibacterial activity	[97]
*LTP4* (*Hordeum vulgare* cv. Bomi)	Q43875.1 (Swiss-Prot) Q42842.1 (Swiss-Prot)	Response to bacterial pathogens	[98]
*BcMF15* (*Brassica campestris* ssp. *chinensis*)	EF600901	Microspore development	[99]
*BcLTP* (*Brassica campestris* ssp. *chinensis*)	EF216852	Secrete and combine extracellular CaM	[100]
*CaMF2* (*Capsicum annuum* L.)	JF411954	Pollen development	[27]
*nsLTP1* (*Ginkgo biloba*)	DQ836633	Proteinase inhibitor	[101]
*LTP* (*Lilium longiflorum* cv. Nellie White)	AF171094	Pollen tube adhesion	[29]
*Ace*-AMP1 (*Allium cepa* L.)	AF004946.1	Antimicrobial	[54]
*OsC6* (*Oryza sativa* L.)	AK064672 (GenBank) OSJNBa0060K21 (EMBL)	Postmeiotic anther development	[28]
*OsDIL* (*Oryza sativa* L.)	Os10g0148000	Drought tolerance	[42]
*MtN5* (*Medicago truncatula*)	MTR_5g094210	Efficient nodulation	[39]
*NtLTP1* (*Nicotiana tabacum* cv. Xanthi)	AB625593	Lipid secretion from glandular trichomes	[102]
*TobLTP2* (*Nicotiana tabacum* cv. Petit Havana SR1)	Q03461 (Swiss-Prot)	Cell wall extension	[34]
*CALTPI* (*Capsicum annuum* L.)	AF208832	Pathogen resistance and long-distance systemic signaling	[38]
*CALTPII* (*Capsicum annuum* L.)	AF208833	Pathogen resistance and long-distance systemic signaling	[38]

## Discussion

The genus *Brassica*, which currently comprises 38 species and numerous varieties, is one of the most significant genera contributing to agriculture. Several species and types of *Brassicas* are cultivated as vegetables, fodder, and sources of oil and condiments [62], [63]. Due to their agricultural importance, *Brassica* plants have been the subject of much scientific interest. Previous studies have indicated that polyploidy has played a crucial role in the evolution of Brassicaceae. The famous U's triangle theory, determined from cytological analyses of inter-specific hybrids, indicates that three diploid species, *B. rapa* (A genome), *Brassica nigra* (B genome), and *Brassica oleracea* (C genome), were the ancestors of the three amphidiploid species, *B. napus* (AC genome), *B. juncea* (AB genome), and *Brassica carinata* (BC genome) [64]. Further genetic linkage analysis has confirmed the relationship between these six widely cultivated *Brassica* species via each possible pair-wise combination [65]–[68]. Additionally, *B. rapa* and Arabidopsis are close relatives [56], and blocks of conserved genome sequence have been identified between these *Brassica* species [69]–[74]. Therefore, the *B. rapa* ‘A’ genome is a valuable resource for studying the evolution of polyploid genomes and underpins the genetic improvement of *Brassica*-related crops. The astonishing current developments in next-generation sequencing technologies provide unprecedented opportunities for decoding genomic information from various plant species. Fortunately, the genome of Chinese cabbage (*B. rapa* subsp. *pekinensis*) has been recently released [56]. The availability of whole genome information can enable us to advance research by focusing on the identification and systematical analysis of large gene families with significant functions. Plant nsLtp is a large transporter family composed of 49 members in Arabidopsis, 52 in rice, 156 in wheat, and 24 in *Lotus japonicus*, all of which are classified as different types [17], [52], [75]. In the present study, we identified 63 putative nsLtps in the genome of *B. rapa*, including 19 type I, 15 type II, three type III, eight type IV, two type V, four type VI, one type VIII, three type IX, six type XI, and two nsLTPY nsLtps (Table 1). Previous studies indicated that in addition to searching for the proteins with the Pfam domain PF00234 against entire proteins databases, Blast searches also identified some putative nsLtps [17], [52]. Similarly, four additional putative *BrnsLtp* genes were found using Blast searches (Fig. 1). Therefore, Blast seems to be a necessary and complementary method for identifying putative and previously unknown nsLtps.

After careful comparison, we found no VII type nsLtps in *B. rapa* similar to those in Arabidopsis (Table 2). Meanwhile, only one type VII nsLtp was found in the monocotyledon rice genome [17]. This may be further evidence that the *B. rapa* is more closely related to the dicotyledon Arabidopsis than to the monocotyledon rice. Moreover, a new type (XI) of nsLtps containing six members was identified in the *B. rapa* genome, which arose during the triplication that occurred after the divergence from Arabidopsis. A recent study showed that nsLtps evolved very early and novel nsLtps types may also have evolved during land plant evolution [51]. Additionally, nine type II *AtLtps* were found to have no homologous genes in the three subgenomes of *B. rapa* (Table 3). Wang et al. [56] identified each of the orthologous blocks in the *B. rapa* genome corresponding to ancestral blocks using collinearity between orthologs on the genomes of *B. rapa* and *A. thaliana* and found significant disparity in the gene loss across the triplicated blocks. Therefore, it was evident that polyploid evolution processes are not simple duplications or triplications but are also likely accompanied by gene mutation and loss. It is an intriguing question as to why *B. rapa* evolved type XI and lost several type II nsLtps. Is it influenced by the relative importance of the genes and their positions on the chromosomes, or is it simply a random phenomenon? Maybe the upcoming release of the *B. napus* (AC) and *B. oleracea* (C) genomes along with further gene function analyses will help to answer these questions. Additionally, the gene duplication analysis demonstrated that 42 of the 63 *BrnsLtp* genes resulted from duplications, including 17 type I, ten type II, two type III, five type IV, three type VI, two type IX, and three type XI BrnsLtps (Fig. 5). Nineteen of the 42 duplicated genes were tandemly duplicated genes, while the other 23 genes were segmentally duplicated genes. This suggests that these two types of duplication events contributed equally to the expansion of the *nsLtp* genes in *B. rapa*. Moreover, we found that only one type VIII and very few type III/V/VI *nsLtp* genes existed in *B. rapa* (Table 1), just like those in Arabidopsis and rice [17]. A previous study demonstrated that four of the types (III/V/VI/VIII) were not identified in six Solanaceae species thus far [18]. Our results also showed that type V/VIII *BrnsLtps* had no duplications (Fig. 5). Why do types III/V/VI/VIII *nsLtps* possess such few members or none at all? It is still an intriguing question that will need to be answered in the future.

**Figure 9:**
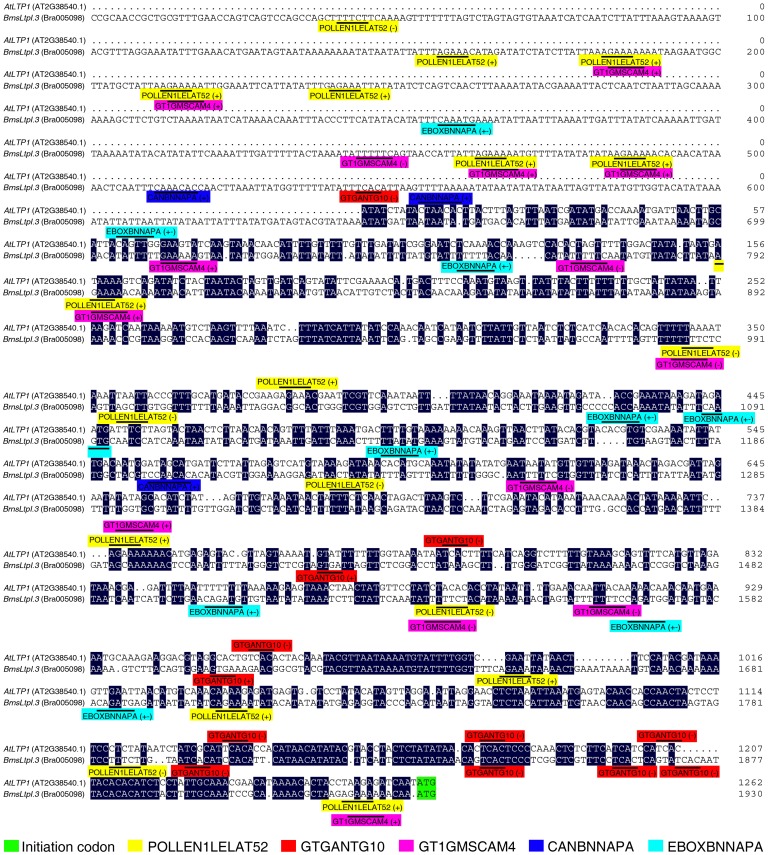
Schematic representation of the selected CREs in the *AtLTP1* and *BrnsLtpI.3* promoters.

Although many putative nsLtps have been identified in the plant kingdom, until now, only a few of their functions have been illustrated. An increasing amount of evidence has suggested that nsLtps may be involved in many biological processes such as defense reactions against phytopathogens, the adaptation of plants to various environmental conditions, and sexual reproduction such as pollen development, pollen tube adhesion and growth, and fertilization (Table 6). As we know, *B. rapa* and other *Brassica*-related crops often suffer various diseases caused by phytopathogens and ultimately lead to yield losses. For example, Sclerotinia stem rot is a world-wide plant disease that results in a 10%–20% yield loss in rapeseed in China; in years of serious illness, this yield loss can be as high as 50%. Previously, Sels et al. [40] had classified some Arabidopsis nsLtps into PR-14 proteins. We found that almost all of these PR-14 proteins belong to type I nsLtps. Amazingly, all these Arabidopsis genes have homologous *nsLtp* genes and duplications in *B. rapa* (data not shown). Therefore, ‘A’ genome of *B. rapa* could help us seek pathogen-resistant genes in *B. napus* (AC genome) and *B. juncea* (AB genome). Furthermore, many nsLtps have already been reported to possess antimicrobial properties, such as Arabidopsis LTP4, DIR, *Ace*-AMP1, CALTPI, and CALTPII (summarized in Table 6). We believed that some nsLtps among the 63 identified BrnsLtps may also have this function, and these elite gene resources may accelerate disease-resistant crop improvement. Additionally, the expression profile of one gene in a particular tissue is an important prerequisite to subsequent elucidation of the corresponding protein required for proper execution of developmental, metabolic and signaling process. Investigation of the expression patterns of the *BrnsLtp* genes demonstrated that 29 *BrnsLtp* genes were specifically or highly expressed in a particular tissue (Fig. 7). Interestingly, 11 *BrnsLtp* genes (*BrnsLtpI.3*, *BrnsLtpI.7/16/18*, *BrnsLtpII.11*, *BrnsLtpII.15*, *BrnsLtpIII.1*, *BrnsLtpIII.2/3*, *BrnsLtpVI.1*, and *BrnsLtpY.1*) (including all three type III *BrnsLtps*) were showed to be specifically expressed in inflorescence (Fig. 7). Liu et al. [18] pointed out that no type III *nsLtps* were identified in six Solanaceae species so far. Do these three type III *BrnsLtps*, with inflorescence-specific expression patterns, have special functions? A previous study indicated that a high rate of lipid biosynthesis in pollen grains starts after pollen mitosis I [76]. This is later corroborated by the high expression levels observed in the tapetum for several enzymes related to lipid biosynthesis in *B. napus* [77]. The lipid biosynthesis during anther development is essential for exine formation [78]. A lipid-rich coat, termed the pollen coat, fills the spaces between the baculae of the exine surface and provides several important functions, such as attachment to pollinators, pollen-stigma interactions, and pathogen attack resistance [79]. Several *Ltps* are expressed in the tapetum, raising the possibility that these genes may function in the transfer of fatty acids and other lipid precursors during pollen wall deposition. Furthermore, stigma/style cysteine-rich adhesin (SCA)-like Arabidopsis Ltps may have diverse roles in plant growth and reproduction [31]. The Arabidopsis LTP1 protein is highly expressed in the cell walls of stigma and pollen grains [47]. Chae et al. [31] also found that *LTP1* was specifically abundant in the stigma, as determined by GUS analysis. Though *LTP5* was found to be the most weakly expressed gene in the inflorescence among SCA-like LTPs, RT-PCR and GUS analyses showed that *LTP5* is present in pollen and the pistil transmitting tract [32]. Additionally, the *E2* gene encoding an Ltp of *B. napus* is exclusively expressed in tapetal cells and developing microspores [80], and the precursor protein sequence of E2 to be almost identical to that of BrnsLtpI.18, with 98.32% identity (data not shown). Actually,our CREs analsysis also showed that all these 11 *BrnsLtps* with inflorescence-specific expression profile had a certain number of POLLEN1LELAT52 and GTGANTG10 elements that belong to late pollen genes. Therefore, these 11 *BrnsLtps* may play important roles in sexual reproduction. After carefully analysis, we found that *BrnsLtpI.3* may be homologous to Arabidopsis *LTP1* (*AtLtpI.5*) (Table 3). Previous studies demonstrated that Arabidopsis LTP1 may function as a calmodulin-binding protein in Arabidopsis, and the distribution of these protein epitopes was associated with morphogenic events during somatic embryogenesis (Table 6). Interestingly, except for 14 POLLEN1LELAT52 and 7 GTGANTG10, we also found 13 GT1GMSCAM4 (belongs to soybean *calmodulin* isoform, *SCaM-4*) [81], two CANBNNAPA (belongs to *B. napus napA* storage-protein gene, and is required for embyo- and endosperm-specific transcription) [82] and ten EBOXBNNAPA (belongs to *B. napus napA* storage-protein gene) [83] CREs existed in the *BrnsLtpI.3* promoter region (Fig. 9). So, we inferred that *BrnsLtpI.3* may have the same functions like Arabidopsis *LTP1*.

The current study has led to the discovery of 63 putative nsLtps in the genome of *B. rapa*, including 19 type I, 15 type II, three type III, eight type IV, two type V, four type VI, one type VIII, three type IX, six type XI, and two nsLTPY nsLtps. Interestingly, type XI appeared as a new type of nsLtps of *B. rapa* containing six members, while nine type II *AtLtps* have no homologous genes in *B. rapa*, indicating that gene loss and mutation are also common events that occur as a consequence of polyploidy. This identification and classification may contribute to increased knowledge regarding the *nsLtp* gene family in plants. Additionally, the expansion of *nsLtp* genes in *B. rapa* was attributed to both segmental and tandem duplications. Based on careful analysis, we hypothesize that some of the type I BrnsLtps may have functions similar to Arabidopsis PR-14 proteins in protecting plants by avoiding phytopathogen attacks. Eleven *BrnsLtps* with inflorescence-specific expression may play important roles in sexual reproduction. Additionally, *BrnsLtpI.3* may have functions similar to Arabidopsis *LTP1*. Going forward, it is important to experimentally characterize these identified *BrnsLtps* to facilitate our understanding of their functions.

## Materials and Methods

### Plant materials

The Chinese cabbage (*B. rapa* ssp. *Pekinensis* line Chiifu-401–42) plants were cultivated at 20±2°C with 12-h light/12-h dark cycles in a greenhouse at the Oil Crops Research Institute (OCRI) of the Chinese Academy of Agricultural Sciences (CAAS). Five tissues (roots, stems, leaves, inflorescence, and siliques) were collected from 10-week-old plants and frozen in liquid nitrogen and stored at −80°C until RNA extraction.

### Identification of BrnsLtps and bioinformatic analysis

All protein files (.pep files) from *B. rapa* were downloaded from the *Brassica* Database (BRAD; http://brassicadb.org/brad/). Proteins with the Pfam domain PF00234 (plant lipid transfer/seed storage/trypsin-alpha amylase inhibitor) were then identified using prediction algorithms (http://pfam.sanger.uk/) [84]. Simultaneously, all the ECMs (C…C…CC…CXC…C…C, where X stands for any amino acid) of AtLtps were used as queries for a BlastP search against all proteins from *B. rapa* with a cut-off value of e^-3^ to avoid the loss of *nsLtp* genes due to mis-annotation and not being annotated. Then, the deduced protein sequences of candidate *nsLtp* genes were manually checked for the presence of the ECMs, and proteins lacking the essential Cys residues were excluded. Subsequently, the proteins without NSSs (checked by the SignalP 4.0, http://www.cbs.dtu.dk/services/SignalP) [85] and with C-terminal GPI anchor signals (predicted by the big-PI Plant Predictor [86] and PSORT [87]), were also removed. After that, the putative proline-rich proteins were excluded from further analyses. The remaining candidate proteins were submitted to the Batch Web CD-Search Tool (http://www.ncbi.nlm.nih.gov/Structure/cdd/wrpsb.cgi) to verify the presence of LTP domains. The protein sequences of the RAT1 [88] and At2S1 [89] were then Blast-searched against the rest of the candidate nsLtp proteins to exclude the possible inhibitors and cereal storage proteins. Additionally, the proteins with more 120 amino acids at maturity were discarded. The pI and MM of each BrnsLtp were calculated by Compute pI/Mw tool (http://web.expasy.org/compute_pi/). The coding sequences (CDSs) of the *BrnsLtp* genes and the protein backbones of BrnsLtps are shown in Tables S1 and S2, respectively. The three-dimensional structures of all putative BrnsLtps were also predicted by Phyre^2^ (http://www.sbg.bio.ic.ac.uk/phyre2/html/page.cgi?id=index) [90] and are shown in Figure S1.

### Sequence and phylogenetic analysis

The genomic DNA sequences of *BrnsLtps* and *AtLtps* were obtained from e!^BrassEnsembl^ (http://www.brassica.info/BrassEnsembl/index.html) and TAIR (http://www.arabidopsis.org), respectively. The number and position of exons and introns were determined by comparing the CDSs with their corresponding genomic DNA sequences, and a map of the gene structure was constructed using a gene structure display server (http://gsds.cbi.pku.edu.cn) [91] and modified by Adobe Photoshop CS3 tool (http://www.photoshop.com). Amino acid sequences were aligned and manually corrected using Clustal X (1.83). The resultant sequence alignments (.fasta file) were converted into a MEGA file (.meg), which then served as input to generate a phylogenetic tree with the Neighbor-Joining algorithm statistical method within MEGA 5.05 software [92]. Bootstrapping was performed 10,000 times to obtain support values for each branch.

Additionally, a Bayesian estimation of phylogeny between BrnsLtps and AtLtps was performed using the MrBayes software (http://mrbayes.csit.fsu.edu/index.php). The MCMC (Markov Chain Monte Carlo) method was used to approximate the posterior probabilities of the trees. The nsLtps.nexus file was generated by MEGA 5.05 using an input file in a fasta format that contained the amino acid sequences of ECMs of BrnsLtps and AtLtps. The commands “execute nsLtps.nexus”, “prset aamodelpr = mixed”, and “mcmc ngen = 1000000 samplefreq = 1000” were consecutively employed in the MrBayes software. The program was stopped after execution of the “sump burnin = 25” command. Ultimately, the phylogram was visualized in TreeView software using file (a.t) as input data.

### Multiple sequence alignment of the ECMs

The amino acid sequences of the ECMs of the 63 BrnsLtps were obtained according to the eight Cys residues. The alignment of these sequences was then conducted and manually edited using the DNAMAN program (http://www.lynnon.com).

### Chromosome localization and gene duplications

The positions of the *BrnsLtps* were mapped to the ten *B. rapa* chromosomes by the BRAD-*Brassica* Genome Browser (http://brassicadb.org/cgi-bin/gbrowse/cbgdb11/). Conserved collinear blocks on the ten chromosomes of the *B. rapa* genome were drawn as described previously [56]. These A to X blocks were color-coded based on the inferred ancestral chromosome following an established convention [93]. The duplication of *BrnsLtps* and the position of each *nsLtp* gene on the syntenic blocks were checked by searching for homologous genes between Arabidopsis and three subgenomes of *B. rapa* (LF, MF1, and MF2) [56] (http://brassicadb.org/brad/searchSynteny.php).

### Digital expression analysis

The total 153,065 expressed sequence tags (ESTs) of *B. rapa* subsp. Pekinensis were downloaded from the NCBI website (ftp://ftp.ncbi.nih.gov/repository/UniGene/Brassica_rapa/) as UniGene files (Bra.seq.all.gz). The file (Bra.seq.all) was used to construct a local formatted database by the program (blast-2.2.26-ia32-win32.exe, ftp://ftp:ncbi.nlm.nih.gov/blast/executables/release/LATEST/) in Windows system. After that, all the CDSs of 63 *BrnsLtps* were saved as fasta format in a query file named “query.fasta”. Then, Basic Local Alignment Search Tool (BLAST) was carried out against the formatted database by using the “query.fasta” file as query. Sequences that satisfied e-value less than 10^−10^ and score-value more than 100 were considered to find the corresponding UniGene number. Finally, the expression profile was suggested by analyzing the EST counts based on UniGene (http://www.ncbi.nlm.nih.gov/UniGene/UGOrg.cgi?TAXID=3711).

### Quantitative RT-PCR analysis

We used TRIzol (Sigma, http://www.sigmaaldrich.com) to extract the total RNA from each sample. An amount of 1 μg of total RNA was first digested with DNase I at 37°C for 30 min to remove DNA contamination, and 1 μl of EDTA (50 mM) was added into the mixture to inactive the DNase I by incubation at 65°C for 10 min. Then, the pretreated RNA was transcribed into first-strand cDNA using a Reverse Transcription System (TOYOBO, http://www.toyobo.co.jp/e/). The synthesized cDNA was diluted ten times by DEPC-treated water and used as template for quantitative RT-PCR. Gene-specific primers or common primers were designed using GeneTool (Table S4, Fig. S2). A housekeeping gene, *Actin* (Table S4), was used to normalize the expression of each gene in different RNA samples. Quantitative RT-PCR analysis was performed by LightCycler@480 SYBR Green I Master using a LightCycler@480II real-time PCR machine (Roche, http://www.roche-applied-science.com), and the relative expression levels were analysed as described by Yuan et al. [94].

### Promoter sequences analysis

The promoter sequences of the *BrnsLtps* and *AtLtps* were obtained from e!^BrassEnsembl^ and SeqViewer (http://tairvm09.tacc.utexas.edu/servlets/sv), respectively. If the length of intergenic region between *nsLtp* and its adjacent upstream gene is more than 2 kb, a 2 kb upstream sequence starts from the ATG initiation code was selected as promoter. The CREs of the *nsLtps* were predicted though PLACE (http://www.dna.affrc.go.jp/PLACE). Then the number of plant CREs belonging to pollen genes in the promoters of selected *BrnsLtps* was manually counted.

## Supporting Information

Figure S1 **Three-dimensional structures of the mature BrnsLtp proteins predicted by Phyre^2^.**
(PDF)

Figure S2 **Bayesian phylogenetic analysis of the nsLtp family in** ***B. rapa*** **using MrBayes software.** The amino acids of the ECMs region were used for the phylogram construction. The different types of nsLtps were marked by a circle or a triangle with different colors. And the accession number of each gene was showed in the parentheses on the right of the corresponding gene name.
(TIF)

Figure S3 **Schematic representation of the primers used in quantitative RT-PCR analysis.**
(PDF)

Figure S4 **Quantitative RT-PCR analysis for selected** ***BrnsLtp*** **genes in tissues and organs of** ***B. rapa*** **with unspecific expression patterns.**
(TIF)

Table S1 **The coding sequences of** ***BrnsLtp*** **genes in** ***B. rapa*** **.**
(DOCX)

Table S2 **The protein backbones of BrnsLtps.**
(DOCX)

Table S3 **List of ESTs of the** ***BrnsLtp*** **genes in** ***B. rapa*** **.**
(XLSX)

Table S4 **Primers used in quantitative RT-PCR.**
(XLSX)

## References

[1] Blein JP, Coutos-Thevenot P, Marion D, Ponchet M (2002) From elicitins to lipid-transfer proteins: a new insight in cell signalling involved in plant defence mechanisms. Trends Plant Sci 7: 293–296. 12119165 10.1016/s1360-1385(02)02284-7

[2] Sleight RG (1987) Intracellular lipid transport in eukaryotes. Annu Rev Physiol 49: 193–208. 3551798 10.1146/annurev.ph.49.030187.001205

[3] Sprong H, van der Sluijs P, van Meer G (2001) How proteins move lipids and lipids move proteins. Nat Rev Mol Cell Biol 2: 504–513. 11433364 10.1038/35080071

[4] Kaplan MR, Simoni RD (1985) Intracellular transport of phosphatidylcholine to the plasma membrane. J Cell Biol 101: 441–445. 4040519 10.1083/jcb.101.2.441 PMC2113683

[5] Vance JE, Aasman EJ, Szarka R (1991) Brefeldin A does not inhibit the movement of phosphatidylethanolamine from its sites for synthesis to the cell surface. J Biol Chem 266: 8241–8247. 2022641

[6] Holthuis JC, Levine TP (2005) Lipid traffic: floppy drives and a superhighway. Nat Rev Mol Cell Biol 6: 209–220. 15738987 10.1038/nrm1591

[7] Levine T (2004) Short-range intracellular trafficking of small molecules across endoplasmic reticulum junctions. Trends Cell Biol 14: 483–490. 15350976 10.1016/j.tcb.2004.07.017

[8] Kader JC (1975) Proteins and the intracellular exchange of lipids. I. Stimulation of phospholipid exchange between mitochondria and microsomal fractions by proteins isolated from potato tuber. Biochim Biophys Acta 380: 31–44. 804327

[9] Ostergaard J, Vergnolle C, Schoentgen F, Kader JC (1993) Acyl-binding/lipid-transfer proteins from rape seedlings, a novel category of proteins interacting with lipids. Biochim Biophys Acta 1170: 109–117. 8399333 10.1016/0005-2760(93)90059-i

[10] Kader JC, Julienne M, Vergnolle C (1984) Purification and characterization of a spinach-leaf protein capable of transferring phospholipids from liposomes to mitochondria or chloroplasts. Eur J Biochem 139: 411–416. 6698022 10.1111/j.1432-1033.1984.tb08020.x

[11] Arondel V, Vergnolle C, Tchang F, Kader JC (1990) Bifunctional lipid-transfer: fatty acid-binding proteins in plants. Mol Cell Biochem 98: 49–56. 2266969 10.1007/BF00231367

[12] Tsuboi S, Osafune T, Tsugeki R, Nishimura M, Yamada M (1992) Nonspecific lipid transfer protein in castor bean cotyledon cells: subcellular localization and a possible role in lipid metabolism. J Biochem 111: 500–508. 1618741 10.1093/oxfordjournals.jbchem.a123787

[13] Jose-Estanyol M, Gomis-Ruth FX, Puigdomenech P (2004) The eight-cysteine motif, a versatile structure in plant proteins. Plant Physiol Biochem 42: 355–365. 15191737 10.1016/j.plaphy.2004.03.009

[14] Douliez JP, Michon T, Elmorjani K, Marion D (2000) Structure, biological and technological functions of lipid transfer proteins and indolines, the major lipid binding proteins from cereal kernels. J Cereal Sci 32: 1–20.

[15] Vergnolle C, Arondel V, Jolliot A, Kader JC (1992) Phospholipid transfer proteins from higher plants. Methods Enzymol 209: 522–530. 1495432 10.1016/0076-6879(92)09063-9

[16] Shin DH, Lee JY, Hwang KY, Kim KK, Suh SW (1995) High-resolution crystal structure of the non-specific lipid-transfer protein from maize seedlings. Structure 3: 189–199. 7735835 10.1016/s0969-2126(01)00149-6

[17] Boutrot F, Chantret N, Gautier MF (2008) Genome-wide analysis of the rice and Arabidopsis *non-specific lipid transfer protein* (*nsLtp*) gene families and identification of wheat nsLtp genes by EST data mining. BMC Genomics 9: 86. 18291034 10.1186/1471-2164-9-86 PMC2277411

[18] Liu W, Huang D, Liu K, Hu S, Yu J, et al (2010) Discovery, Identification and Comparative Analysis of Non-Specific Lipid Transfer Protein (nsLtp) Family in Solanaceae. Genomics, Proteomics & Bioinformatics 8: 229–237. 10.1016/S1672-0229(10)60024-1 PMC5054125 21382591

[19] Kader JC (1996) Lipid-Transfer Proteins in Plants. Annu Rev Plant Physiol Plant Mol Biol 47: 627–654. 15012303 10.1146/annurev.arplant.47.1.627

[20] Carvalho Ade O, Gomes VM (2007) Role of plant lipid transfer proteins in plant cell physiology-a concise review. Peptides 28: 1144–1153. 17418913 10.1016/j.peptides.2007.03.004

[21] Kim TH, Park JH, Kim MC, Cho SH (2008) Cutin monomer induces expression of the rice *OsLTP5* lipid transfer protein gene. J Plant Physiol 165: 345–349. 17765359 10.1016/j.jplph.2007.06.004

[22] Cameron KD, Teece MA, Smart LB (2006) Increased accumulation of cuticular wax and expression of lipid transfer protein in response to periodic drying events in leaves of tree tobacco. Plant Physiol 140: 176–183. 16361524 10.1104/pp.105.069724 PMC1326042

[23] Lee SB, Go YS, Bae HJ, Park JH, Cho SH, et al (2009) Disruption of glycosylphosphatidylinositol-anchored lipid transfer protein gene altered cuticular lipid composition, increased plastoglobules, and enhanced susceptibility to infection by the fungal pathogen *Alternaria brassicicola* . Plant Physiol 150: 42–54. 19321705 10.1104/pp.109.137745 PMC2675750

[24] Sterk P, Booij H, Schellekens GA, Van Kammen A, De Vries SC (1991) Cell-specific expression of the carrot EP2 lipid transfer protein gene. Plant Cell 3: 907–921. 1822991 10.1105/tpc.3.9.907 PMC160059

[25] Coutos-Thevenot P, Jouenne T, Maes O, Guerbette F, Grosbois M, et al (1993) Four 9-kDa proteins excreted by somatic embryos of grapevine are isoforms of lipid-transfer proteins. Eur J Biochem 217: 885–889. 8223644 10.1111/j.1432-1033.1993.tb18317.x

[26] Potocka I, Baldwin TC, Kurczynska EU (2012) Distribution of lipid transfer protein 1 (LTP1) epitopes associated with morphogenic events during somatic embryogenesis of *Arabidopsis thaliana* . Plant Cell Rep 31: 2031–2045. 22821363 10.1007/s00299-012-1314-0 PMC3472069

[27] Chen C, Chen G, Hao X, Cao B, Chen Q, et al (2011) *CaMF2*, an anther-specific lipid transfer protein (LTP) gene, affects pollen development in *Capsicum annuum* L. Plant Sci. 181: 439–448. 10.1016/j.plantsci.2011.07.003 21889050

[28] Zhang D, Liang W, Yin C, Zong J, Gu F, et al (2010) *OsC6*, encoding a lipid transfer protein, is required for postmeiotic anther development in rice. Plant Physiol 154: 149–162. 20610705 10.1104/pp.110.158865 PMC2938136

[29] Park SY, Jauh GY, Mollet JC, Eckard KJ, Nothnagel EA, et al (2000) A lipid transfer-like protein is necessary for lily pollen tube adhesion to an *in vitro* stylar matrix. Plant Cell 12: 151–164. 10634914 10.1105/tpc.12.1.151 PMC140221

[30] Park SY, Lord EM (2003) Expression studies of SCA in lily and confirmation of its role in pollen tube adhesion. Plant Mol Biol 51: 183–189. 12602877 10.1023/a:1021139502947

[31] Chae K, Gonong BJ, Kim SC, Kieslich CA, Morikis D, et al (2010) A multifaceted study of stigma/style cysteine-rich adhesin (SCA)-like Arabidopsis lipid transfer proteins (LTPs) suggests diversified roles for these LTPs in plant growth and reproduction. J Exp Bot 61: 4277–4290. 20667964 10.1093/jxb/erq228 PMC2955742

[32] Chae K, Kieslich CA, Morikis D, Kim SC, Lord EM (2009) A gain-of-function mutation of Arabidopsis lipid transfer protein 5 disturbs pollen tube tip growth and fertilization. Plant Cell 21: 3902–3914. 20044438 10.1105/tpc.109.070854 PMC2814499

[33] Chae K, Lord EM (2011) Pollen tube growth and guidance: roles of small, secreted proteins. Ann Bot 108: 627–636. 21307038 10.1093/aob/mcr015 PMC3170145

[34] Nieuwland J, Feron R, Huisman BA, Fasolino A, Hilbers CW, et al (2005) Lipid transfer proteins enhance cell wall extension in tobacco. Plant Cell 17: 2009–2019. 15937228 10.1105/tpc.105.032094 PMC1167548

[35] Guiderdoni E, Cordero MJ, Vignols F, Garcia-Garrido JM, Lescot M, et al (2002) Inducibility by pathogen attack and developmental regulation of the rice *Ltp1* gene. Plant Mol Biol 49: 683–699. 12081375 10.1023/a:1015595100145

[36] Jung HW, Kim W, Hwang BK (2003) Three pathogen-inducible genes encoding lipid transfer protein from pepper are differentially activated by pathogens, abiotic, and environmental stresses. Plant Cell Environ 26: 915–928. 12803619 10.1046/j.1365-3040.2003.01024.x

[37] Lin P, Xia L, Ng TB (2007) First isolation of an antifungal lipid transfer peptide from seeds of a Brassica species. Peptides 28: 1514–1519. 17692430 10.1016/j.peptides.2007.06.028

[38] Sarowar S, Kim YJ, Kim KD, Hwang BK, Ok SH, et al (2009) Overexpression of lipid transfer protein (LTP) genes enhances resistance to plant pathogens and LTP functions in long-distance systemic signaling in tobacco. Plant Cell Rep 28: 419–427. 19089429 10.1007/s00299-008-0653-3

[39] Pii Y, Molesini B, Pandolfini T (2013) The involvement of *Medicago truncatula* non-specific lipid transfer protein N5 in the control of rhizobial infection. Plant Signal Behav 8. 10.4161/psb.24836 PMC3909036 23656864

[40] Sels J, Mathys J, De Coninck BM, Cammue BP, De Bolle MF (2008) Plant pathogenesis-related (PR) proteins: a focus on PR peptides. Plant Physiol Biochem 46: 941–950. 18674922 10.1016/j.plaphy.2008.06.011

[41] Schweiger W, Steiner B, Ametz C, Siegwart G, Wiesenberger G, et al (2013) Transcriptomic characterization of two major *Fusarium* resistance quantitative trait loci (QTLs), *Fhb1* and *Qfhs.ifa-5A*, identifies novel candidate genes. Mol Plant Pathol 14: 772–785. 23738863 10.1111/mpp.12048 PMC3902993

[42] Guo C, Ge X, Ma H (2013) The rice *OsDIL* gene plays a role in drought tolerance at vegetative and reproductive stages. Plant Mol Biol 82: 239–253. 23686450 10.1007/s11103-013-0057-9

[43] Guo L, Yang H, Zhang X, Yang S (2013) *Lipid transfer protein 3* as a target of MYB96 mediates freezing and drought stress in Arabidopsis. J Exp Bot 64: 1755–1767. 23404903 10.1093/jxb/ert040 PMC3617838

[44] Giordani T, Buti M, Natali L, Pugliesi C, Cattonaro F, et al (2011) An analysis of sequence variability in eight genes putatively involved in drought response in sunflower (*Helianthus annuus* L.). Theor Appl Genet 122: 1039–1049. 21184050 10.1007/s00122-010-1509-0

[45] Maldonado AM, Doerner P, Dixon RA, Lamb CJ, Cameron RK (2002) A putative lipid transfer protein involved in systemic resistance signalling in Arabidopsis. Nature 419: 399–403. 12353036 10.1038/nature00962

[46] Pii Y, Pandolfini T, Crimi M (2010) Signaling LTPs: A new plant LTPs sub-family? Plant Signal Behav 5. 10.4161/psb.11499 PMC7080482 20404561

[47] Thoma S, Hecht U, Kippers A, Botella J, De Vries S, et al (1994) Tissue-specific expression of a gene encoding a cell wall-localized lipid transfer protein from Arabidopsis. Plant Physiol 105: 35–45. 8029357 10.1104/pp.105.1.35 PMC159326

[48] Wang NJ, Lee CC, Cheng CS, Lo WC, Yang YF, et al (2012) Construction and analysis of a plant non-specific lipid transfer protein database (nsLTPDB). BMC Genomics 13 Suppl 1S9. 10.1186/1471-2164-13-S1-S9 PMC3303721 22369214

[49] Jose-Estanyol M, Puigdomenech P (2000) Plant cell wall glycoproteins and their genes. Plant Physiology and Biochemistry 38: 97–108.

[50] Dvorakova L, Cvrckova F, Fischer L (2007) Analysis of the hybrid proline-rich protein families from seven plant species suggests rapid diversification of their sequences and expression patterns. BMC Genomics 8: 412. 17997832 10.1186/1471-2164-8-412 PMC2216038

[51] Edstam MM, Viitanen L, Salminen TA, Edqvist J (2011) Evolutionary history of the non-specific lipid transfer proteins. Mol Plant 4: 947–964. 21486996 10.1093/mp/ssr019

[52] Wang HW, Hwang SG, Karuppanapandian T, Liu A, Kim W, et al (2012) Insight into the molecular evolution of non-specific lipid transfer proteins via comparative analysis between rice and sorghum. DNA Res 19: 179–194. 22368182 10.1093/dnares/dss003 PMC3325081

[53] Lehmann MS, Pebay-Peyroula E, Cohen-Addad C, Odani S (1989) Crystallographic data for soybean hydrophobic protein. J Mol Biol 210: 235–236. 2585518 10.1016/0022-2836(89)90303-3

[54] Tassin S, Broekaert WF, Marion D, Acland DP, Ptak M, et al (1998) Solution structure of *Ace-AMP1*, a potent antimicrobial protein extracted from onion seeds. Structural analogies with plant nonspecific lipid transfer proteins. Biochemistry 37: 3623–3637. 9521681 10.1021/bi9723515

[55] Long M, Rosenberg C, Gilbert W (1995) Intron phase correlations and the evolution of the intron/exon structure of genes. Proc Natl Acad Sci U S A 92: 12495–12499. 8618928 10.1073/pnas.92.26.12495 PMC40384

[56] Wang X, Wang H, Wang J, Sun R, Wu J, et al (2011) The genome of the mesopolyploid crop species *Brassica rapa* . Nat Genet 43: 1035–1039. 21873998 10.1038/ng.919

[57] Cannon SB, Mitra A, Baumgarten A, Young ND, May G (2004) The roles of segmental and tandem gene duplication in the evolution of large gene families in *Arabidopsis thaliana* . BMC Plant Biol 4: 10. 15171794 10.1186/1471-2229-4-10 PMC446195

[58] Bate N, Twell D (1998) Functional architecture of a late pollen promoter: pollen-specific transcription is developmentally regulated by multiple stage-specific and co-dependent activator elements. Plant Mol Biol 37: 859–869. 9678581 10.1023/a:1006095023050

[59] Rogers HJ, Bate N, Combe J, Sullivan J, Sweetman J, et al (2001) Functional analysis of cis-regulatory elements within the promoter of the tobacco late pollen gene *g10* . Plant Mol Biol 45: 577–585. 11414616 10.1023/a:1010695226241

[60] Champigny MJ, Shearer H, Mohammad A, Haines K, Neumann M, et al (2011) Localization of DIR1 at the tissue, cellular and subcellular levels during Systemic Acquired Resistance in Arabidopsis using DIR1:GUS and DIR1:EGFP reporters. BMC Plant Biol 11: 125. 21896186 10.1186/1471-2229-11-125 PMC3180652

[61] Champigny MJ, Isaacs M, Carella P, Faubert J, Fobert PR, et al (2013) Long distance movement of DIR1 and investigation of the role of DIR1-like during systemic acquired resistance in Arabidopsis. Front Plant Sci 4: 230. 23847635 10.3389/fpls.2013.00230 PMC3701462

[62] Cardoza V, Stewart CN (2004) Invited review: Brassica biotechnology: Progress in cellular and molecular biology. In Vitro Cellular & Developmental Biology-Plant 40: 542–551.

[63] Cheng F, Mandakova T, Wu J, Xie Q, Lysak MA, et al (2013) Deciphering the diploid ancestral genome of the Mesohexaploid *Brassica rapa* . Plant Cell 25: 1541–1554. 23653472 10.1105/tpc.113.110486 PMC3694691

[64] U N (1935) Genome analysis in *Brassica* with special reference to the experimental formation of *B. napus* and peculiar mode of fertilization. Jpn J Bot 7: 389–452.

[65] Parkin IA, Sharpe AG, Keith DJ, Lydiate DJ (1995) Identification of the A and C genomes of amphidiploid *Brassica napus* (oilseed rape). Genome 38: 1122–1131. 18470236 10.1139/g95-149

[66] Bohuon EJ, Keith DJ, Parkin IA, Sharpe AG, Lydiate DJ (1996) Alignment of the conserved C genomes of *Brassica oleracea* and *Brassica napus* . Theor Appl Genet 93: 833–839. 24162415 10.1007/BF00224083

[67] Panjabi P, Jagannath A, Bisht NC, Padmaja KL, Sharma S, et al (2008) Comparative mapping of *Brassica juncea* and *Arabidopsis thaliana* using Intron Polymorphism (IP) markers: homoeologous relationships, diversification and evolution of the A, B and C Brassica genomes. BMC Genomics 9: 113. 18315867 10.1186/1471-2164-9-113 PMC2277410

[68] Suwabe K, Morgan C, Bancroft I (2008) Integration of Brassica A genome genetic linkage map between *Brassica napus* and *B. rapa* . Genome 51: 169–176. 18356952 10.1139/G07-113

[69] Lukens L, Zou F, Lydiate D, Parkin I, Osborn T (2003) Comparison of a *Brassica oleracea* genetic map with the genome of *Arabidopsis thaliana* . Genetics 164: 359–372. 12750346 10.1093/genetics/164.1.359 PMC1462567

[70] Parkin IA, Sharpe AG, Lydiate DJ (2003) Patterns of genome duplication within the *Brassica napus* genome. Genome 46: 291–303. 12723045 10.1139/g03-006

[71] Parkin IA, Gulden SM, Sharpe AG, Lukens L, Trick M, et al (2005) Segmental structure of the *Brassica napus* genome based on comparative analysis with *Arabidopsis thaliana* . Genetics 171: 765–781. 16020789 10.1534/genetics.105.042093 PMC1456786

[72] Lim GA, Jewell EG, Li X, Erwin TA, Love C, et al (2007) A comparative map viewer integrating genetic maps for Brassica and Arabidopsis. BMC Plant Biol 7: 40. 17645810 10.1186/1471-2229-7-40 PMC1940001

[73] Lysak MA, Koch MA, Pecinka A, Schubert I (2005) Chromosome triplication found across the tribe *Brassiceae* . Genome Res 15: 516–525. 15781573 10.1101/gr.3531105 PMC1074366

[74] Lysak MA, Cheung K, Kitschke M, Bures P (2007) Ancestral chromosomal blocks are triplicated in Brassiceae species with varying chromosome number and genome size. Plant Physiol 145: 402–410. 17720758 10.1104/pp.107.104380 PMC2048728

[75] Tapia G, Morales-Quintana L, Parra C, Berbel A, Alcorta M (2013) Study of nsLTPs in *Lotus japonicus* genome reveal a specific epidermal cell member (LjLTP10) regulated by drought stress in aerial organs with a putative role in cutin formation. Plant Mol Biol 82: 485–501. 23733601 10.1007/s11103-013-0080-x

[76] Evans DE, Taylor PE, Singh MB, Knox RB (1992) The interrelationship between the accumulation of lipids, protein and the level of acyl carrier protein during the development of *Brassica napus* L. Planta. 186: 343–354. 10.1007/BF00195314 24186730

[77] Piffanelli P, Ross JH, Murphy DJ (1997) Intra- and extracellular lipid composition and associated gene expression patterns during pollen development in *Brassica napus* . Plant J 11: 549–562. 9107041 10.1046/j.1365-313x.1997.11030549.x

[78] Zhou Z, Dun X, Xia S, Shi D, Qin M, et al (2012) *BnMs3* is required for tapetal differentiation and degradation, microspore separation, and pollen-wall biosynthesis in *Brassica napus* . J Exp Bot 63: 2041–2058. 22174440 10.1093/jxb/err405 PMC3295392

[79] Boavida LC, Becker JD, Feijo JA (2005) The making of gametes in higher plants. Int J Dev Biol 49: 595–614. 16096968 10.1387/ijdb.052019lb

[80] Foster GD, Robinson SW, Blundell RP, Roberts MR, Hodge R (1992) A *Brassica napus* mRNA encoding a protein homologous to phospholipid transfer proteins, is expressed especially in the tapetum and developing microspores. Plant Sci 84: 184–192.

[81] Park HC, Kim ML, Kang YH, Jeon JM, Yoo JH, et al (2004) Pathogen- and NaCl-induced expression of the *SCaM-4* promoter is mediated in part by a GT-1 box that interacts with a GT-1-like transcription factor. Plant Physiol 135: 2150–2161. 15310827 10.1104/pp.104.041442 PMC520786

[82] Ellerstrom M, Stalberg K, Ezcurra I, Rask L (1996) Functional dissection of a napin gene promoter: identification of promoter elements required for embryo and endosperm-specific transcription. Plant Mol Biol 32: 1019–1027. 9002600 10.1007/BF00041385

[83] Stalberg K, Ellerstom M, Ezcurra I, Ablov S, Rask L (1996) Disruption of an overlapping E-box/ABRE motif abolished high transcription of the napA storage-protein promoter in transgenic *Brassica napus* seeds. Planta 199: 515–519. 8818291 10.1007/BF00195181

[84] Finn RD, Mistry J, Schuster-Bockler B, Griffiths-Jones S, Hollich V, et al (2006) Pfam: clans, web tools and services. Nucleic Acids Res 34: D247–251. 16381856 10.1093/nar/gkj149 PMC1347511

[85] Petersen TN, Brunak S, von Heijne G, Nielsen H (2011) SignalP 4.0: discriminating signal peptides from transmembrane regions. Nat Methods 8: 785–786. 21959131 10.1038/nmeth.1701

[86] Eisenhaber B, Wildpaner M, Schultz CJ, Borner GH, Dupree P, et al (2003) Glycosylphosphatidylinositol lipid anchoring of plant proteins. Sensitive prediction from sequence- and genome-wide studies for Arabidopsis and rice. Plant Physiol 133: 1691–1701. 14681532 10.1104/pp.103.023580 PMC300724

[87] Nakai K, Horton P (1999) PSORT: a program for detecting sorting signals in proteins and predicting their subcellular localization. Trends Biochem Sci 24: 34–36. 10087920 10.1016/s0968-0004(98)01336-x

[88] Alam N, Gourinath S, Dey S, Srinivasan A, Singh TP (2001) Substrate-inhibitor interactions in the kinetics of alpha-amylase inhibition by ragi alpha-amylase/trypsin inhibitor (RATI) and its various N-terminal fragments. Biochemistry 40: 4229–4233. 11284678 10.1021/bi002537v

[89] Guerche P, Tire C, De Sa FG, De Clercq A, Van Montagu M, et al (1990) Differential Expression of the Arabidopsis 2S Albumin Genes and the Effect of Increasing Gene Family Size. Plant Cell 2: 469–478. 12354963 10.1105/tpc.2.5.469 PMC159903

[90] Kelley LA, Sternberg MJ (2009) Protein structure prediction on the Web: a case study using the Phyre server. Nat Protoc 4: 363–371. 19247286 10.1038/nprot.2009.2

[91] Guo AY, Zhu QH, Chen X, Luo JC (2007) GSDS: a gene structure display server. Yi Chuan 29: 1023–1026. 17681935

[92] Tamura K, Peterson D, Peterson N, Stecher G, Nei M, et al (2011) MEGA5: molecular evolutionary genetics analysis using maximum likelihood, evolutionary distance, and maximum parsimony methods. Mol Biol Evol 28: 2731–2739. 21546353 10.1093/molbev/msr121 PMC3203626

[93] Schranz ME, Lysak MA, Mitchell-Olds T (2006) The ABC's of comparative genomics in the Brassicaceae: building blocks of crucifer genomes. Trends Plant Sci 11: 535–542. 17029932 10.1016/j.tplants.2006.09.002

[94] Yuan J, Chen D, Ren Y, Zhang X, Zhao J (2008) Characteristic and expression analysis of a metallothionein gene, *OsMT2b*, down-regulated by cytokinin suggests functions in root development and seed embryo germination of rice. Plant Physiol 146: 1637–1650. 18258694 10.1104/pp.107.110304 PMC2287339

[95] Wang Z, Xie W, Chi F, Li C (2005) Identification of non-specific lipid transfer protein-1 as a calmodulin-binding protein in Arabidopsis. FEBS Lett 579: 1683–1687. 15757661 10.1016/j.febslet.2005.02.024

[96] Brotman Y, Lisec J, Meret M, Chet I, Willmitzer L, et al (2012) Transcript and metabolite analysis of the *Trichoderma*-induced systemic resistance response to *Pseudomonas syringae* in *Arabidopsis thaliana* . Microbiology 158: 139–146. 21852347 10.1099/mic.0.052621-0

[97] Molina A, Garcia-Olmedo F (1997) Enhanced tolerance to bacterial pathogens caused by the transgenic expression of barley lipid transfer protein LTP2. Plant J 12: 669–675. 9351251 10.1046/j.1365-313x.1997.00669.x

[98] Molina A, Diaz I, Vasil IK, Carbonero P, Garcia-Olmedo F (1996) Two cold-inducible genes encoding lipid transfer protein LTP4 from barley show differential responses to bacterial pathogens. Mol Gen Genet 252: 162–168. 8804389 10.1007/BF02173216

[99] Tian A, Cao J, Huang L, Yu X, Ye W (2009) Characterization of a male sterile related gene *BcMF15* from *Brassica campestris* ssp. *chinensis* . Mol Biol Rep 36: 307–314. 18034318 10.1007/s11033-007-9180-5

[100] Wang C, Xie W, Chi F, Hu W, Mao G, et al (2008) BcLTP, a novel lipid transfer protein in *Brassica chinensis*, may secrete and combine extracellular CaM. Plant Cell Rep 27: 159–169. 17891402 10.1007/s00299-007-0434-4

[101] Sawano Y, Hatano K, Miyakawa T, Komagata H, Miyauchi Y, et al (2008) Proteinase inhibitor from ginkgo seeds is a member of the plant nonspecific lipid transfer protein gene family. Plant Physiol 146: 1909–1919. 18305212 10.1104/pp.107.111500 PMC2287358

[102] Choi YE, Lim S, Kim HJ, Han JY, Lee MH, et al (2012) Tobacco *NtLTP1*, a glandular-specific lipid transfer protein, is required for lipid secretion from glandular trichomes. Plant J 70: 480–491. 22171964 10.1111/j.1365-313X.2011.04886.x

