# Supplementary material for: Genome-Wide Survey and Expression Analysis of the Putative Non-Specific Lipid Transfer Proteins in Brassica rapa L
Source: PLoS One. 2014 Jan 31;9(1):e84556. doi: 10.1371/journal.pone.0084556 (PMC3908880; doi:10.1371/journal.pone.0084556)
Supplement: Figure S1 — Three-dimensional structures of the mature BrnsLtp proteins predicted by Phyre2. (PDF) [file pone.0084556.s001.pdf]

Figure S1. Three-dimensional structures of the mature BrnsLtp proteins predicted by Phyre[2]

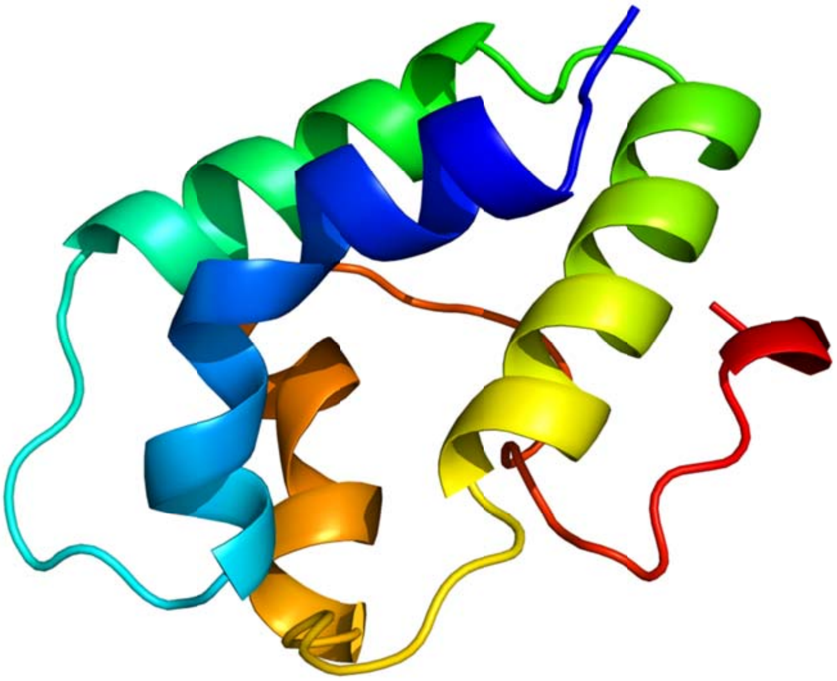

BrnsLtpI.1

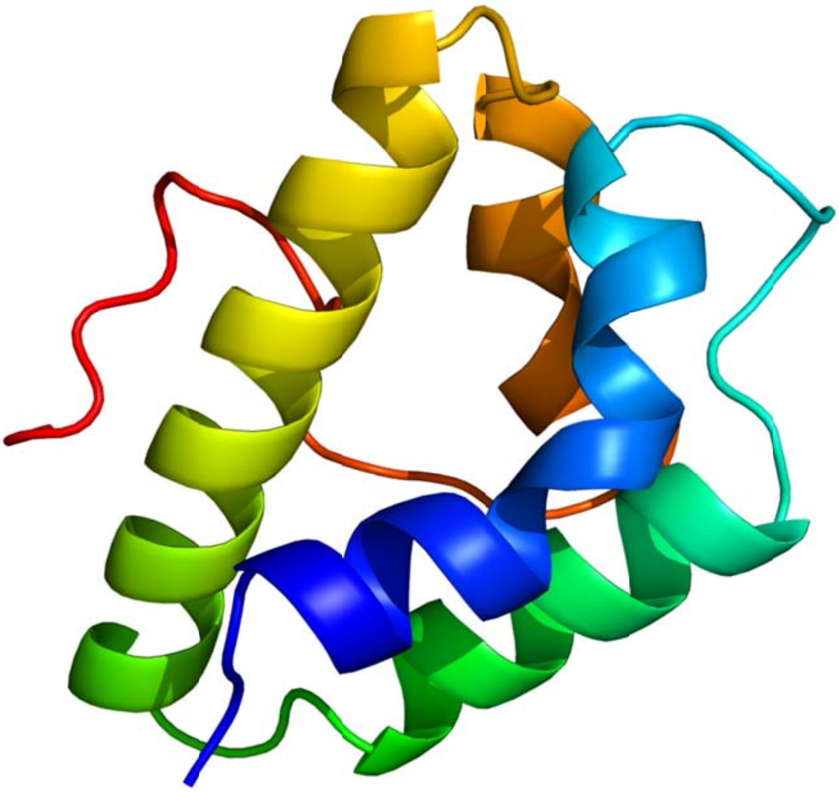

BrnsLtpI.2

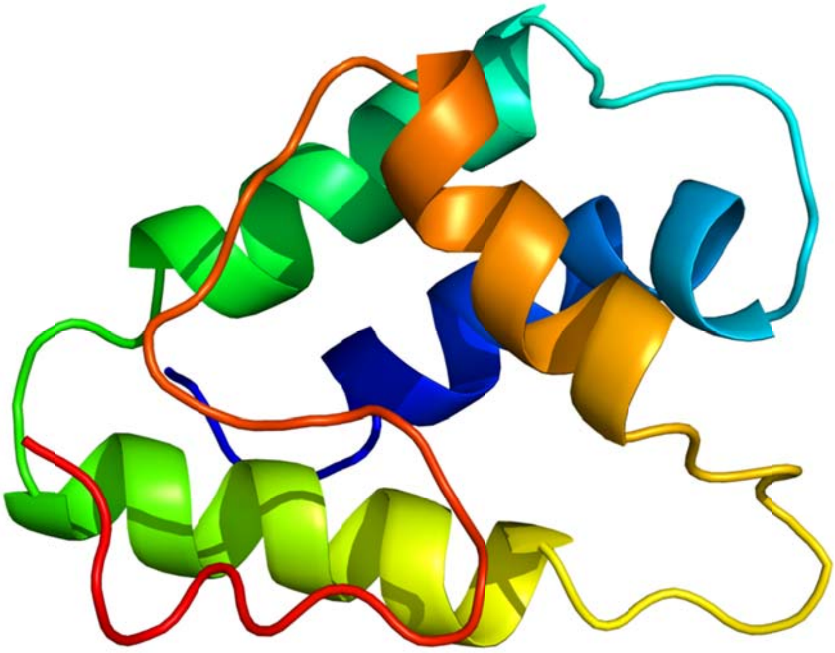

BrnsLtpI.3

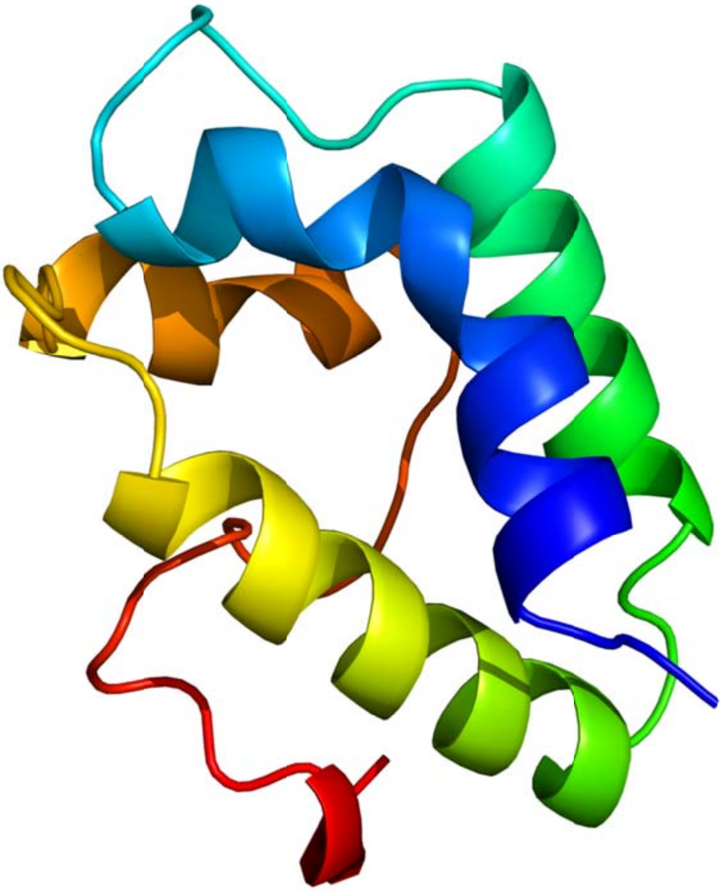

BrnsLtpI.4

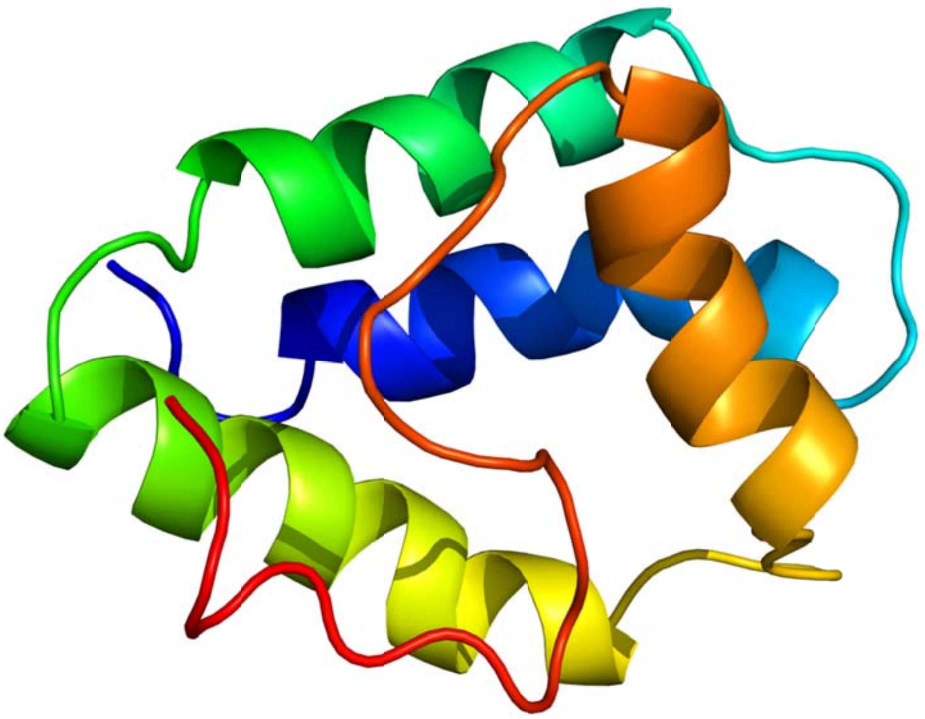

BrnsLtpI.5

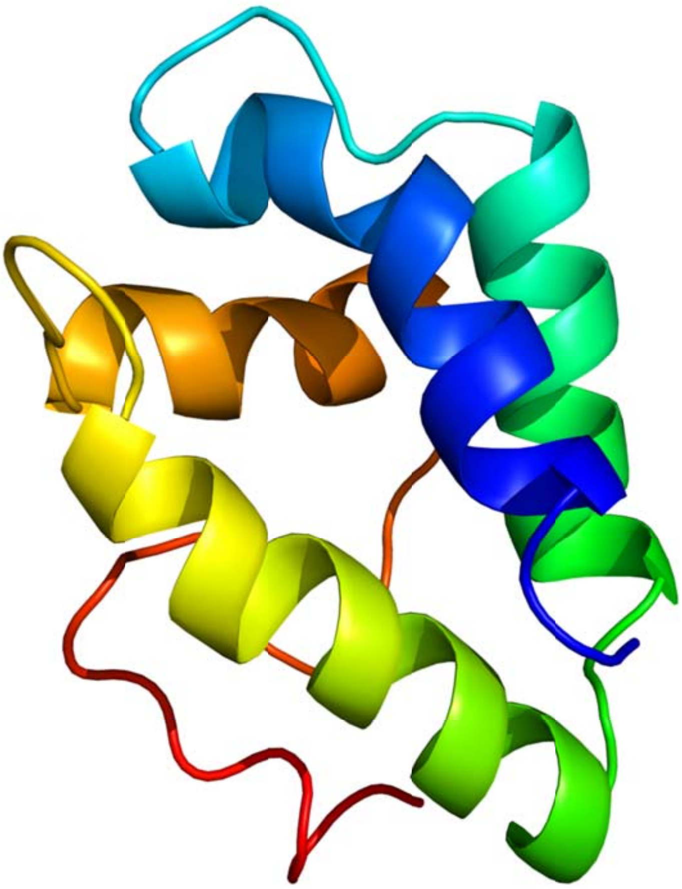

BrnsLtpI.6

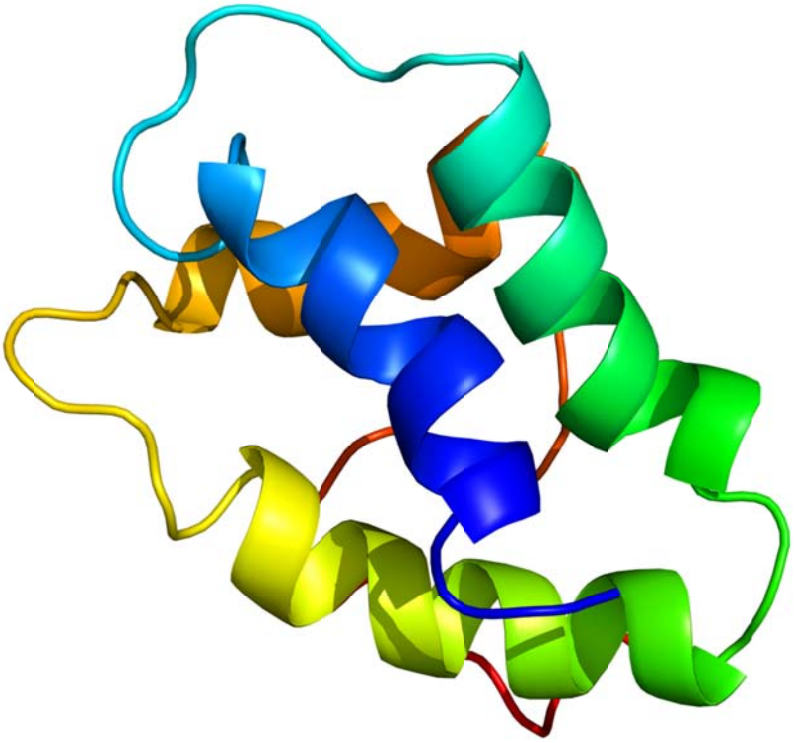

BrnsLtpI.7

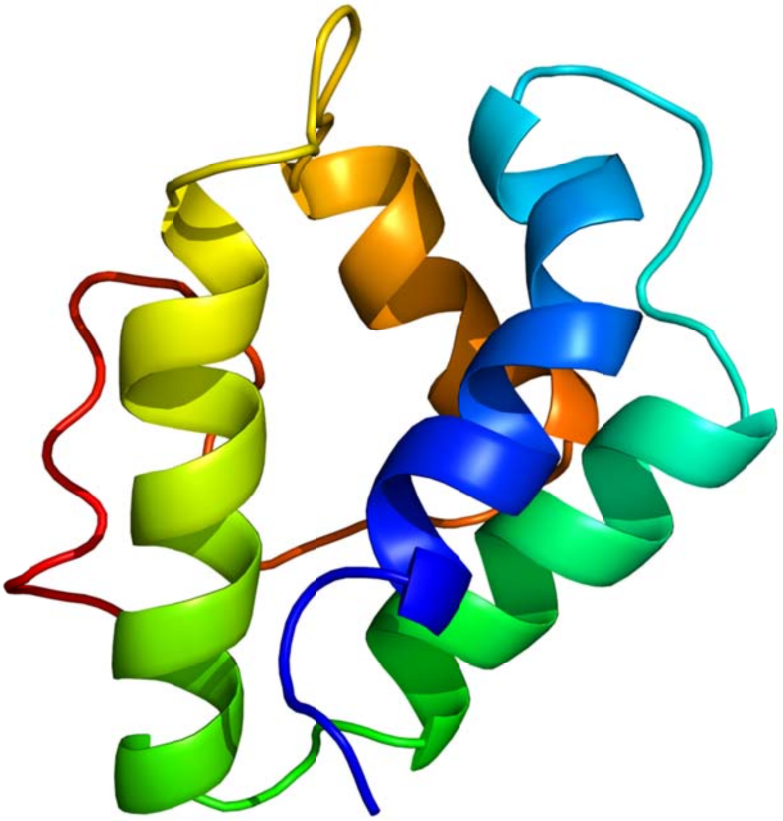

BrnsLtpI.8

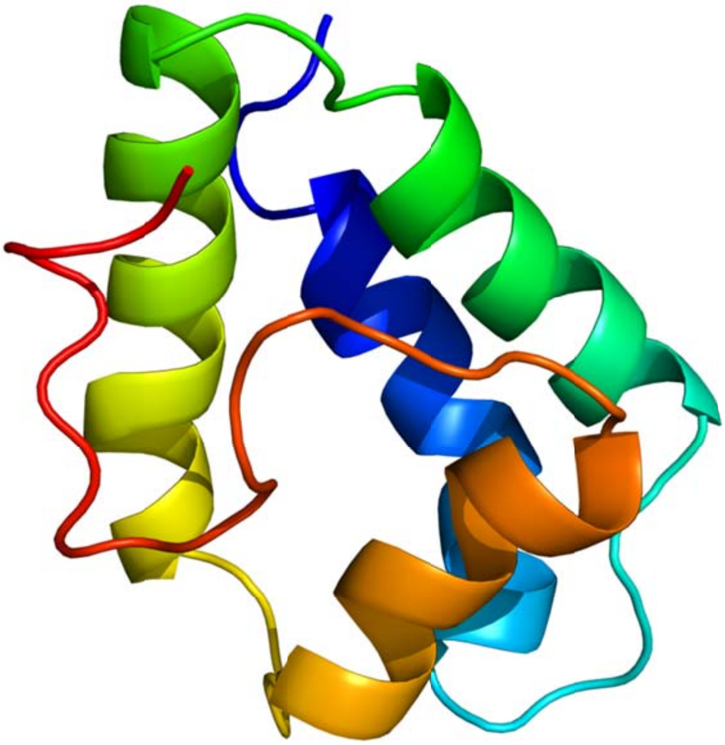

BrnsLtpI.9

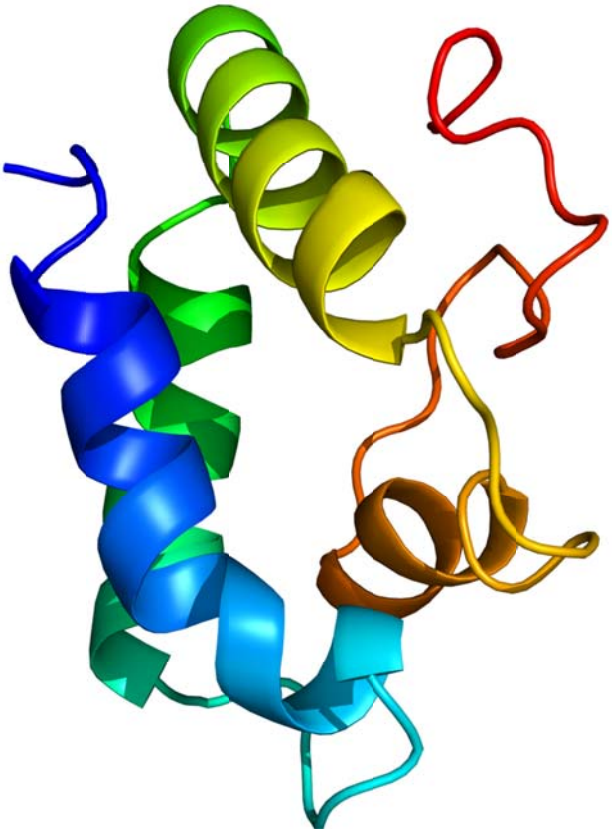

BrnsLtpI.10

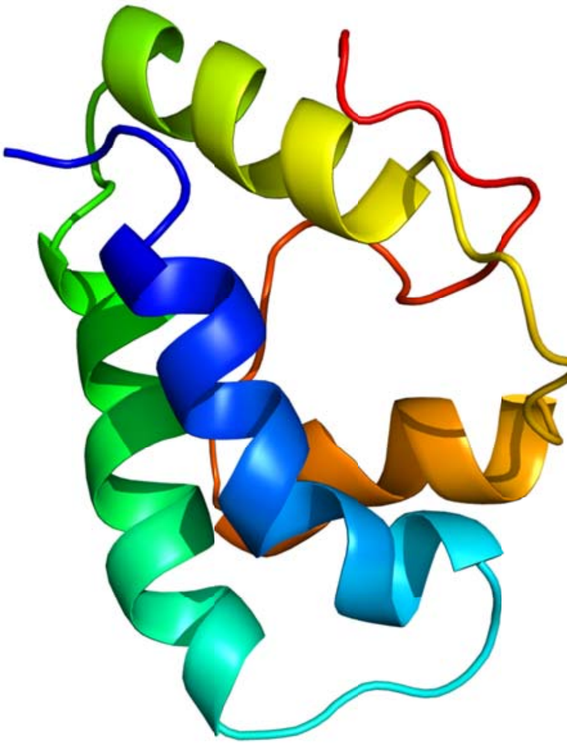

BrnsLtpI.11

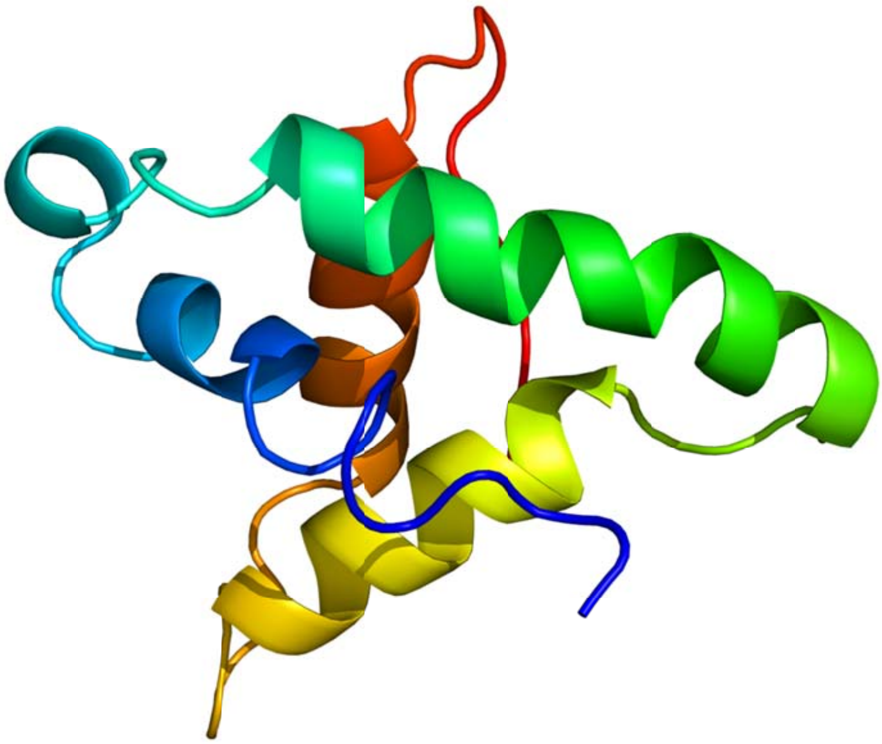

BrnsLtpI.12

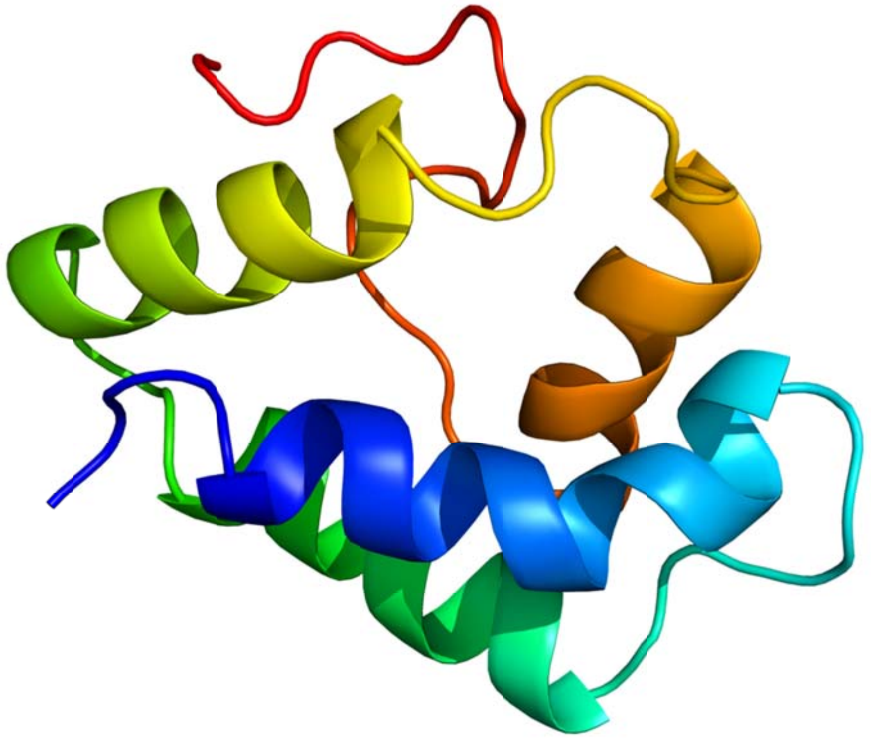

BrnsLtpI.13

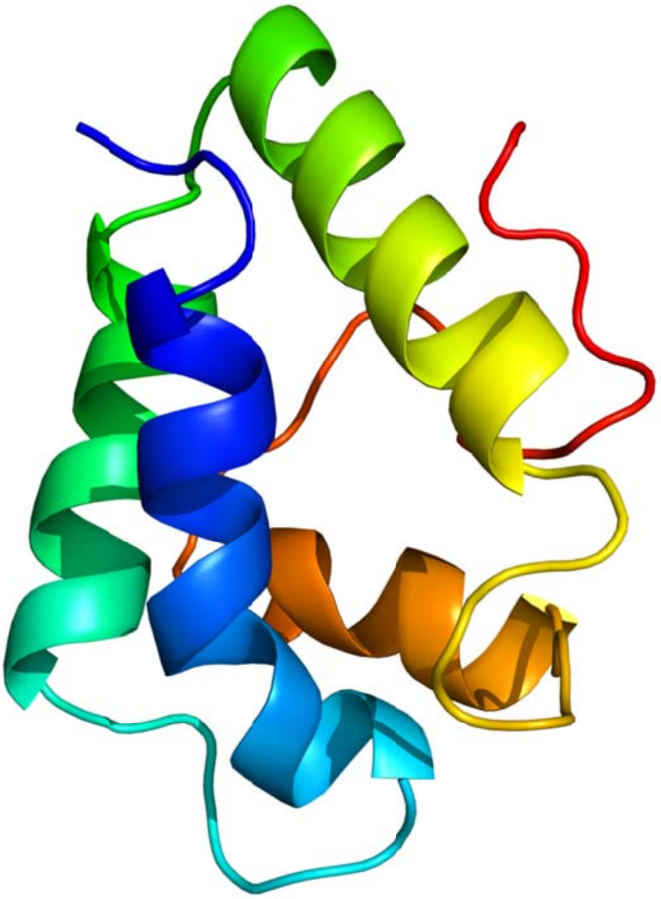

BrnsLtpI.14

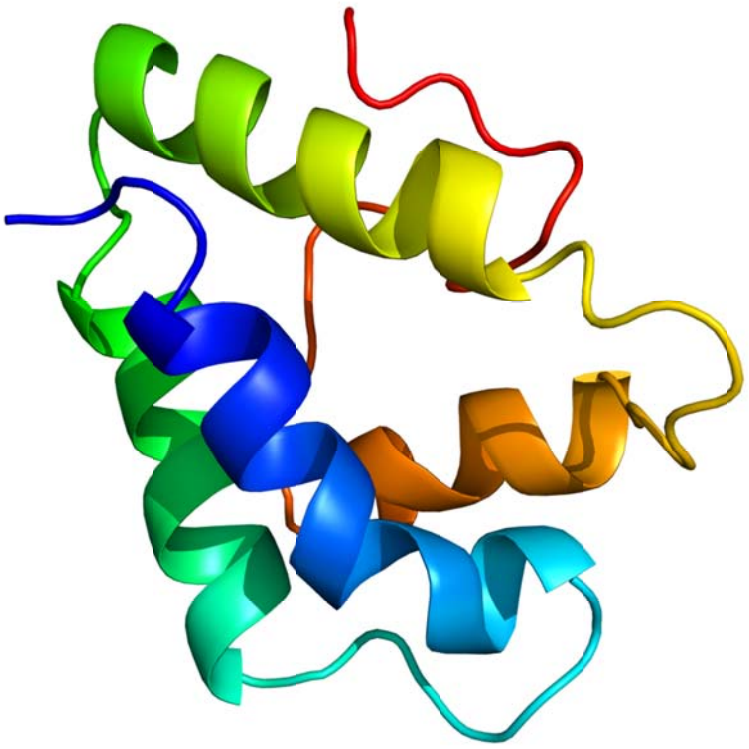

BrnsLtpI.15

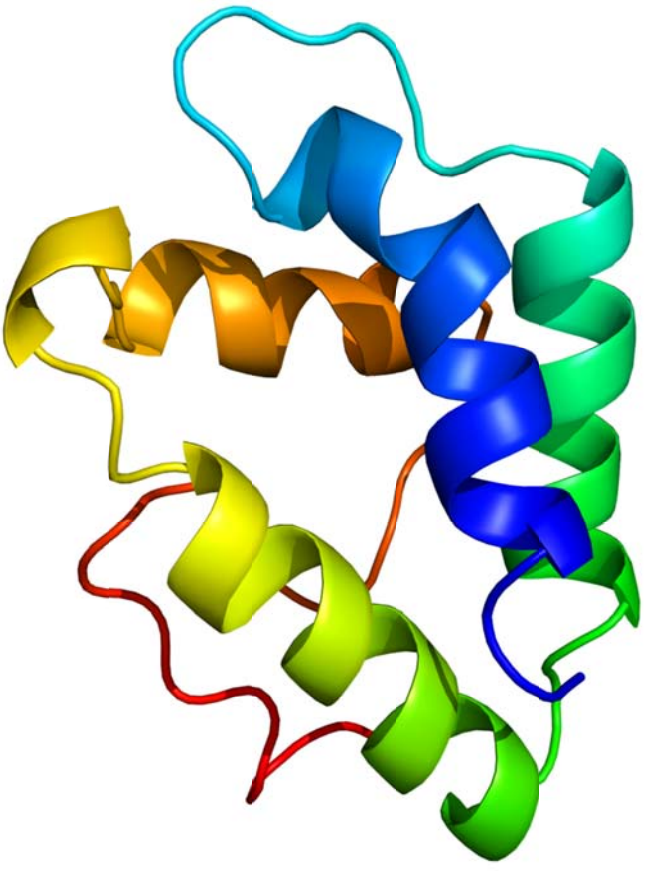

BrnsLtpI.16

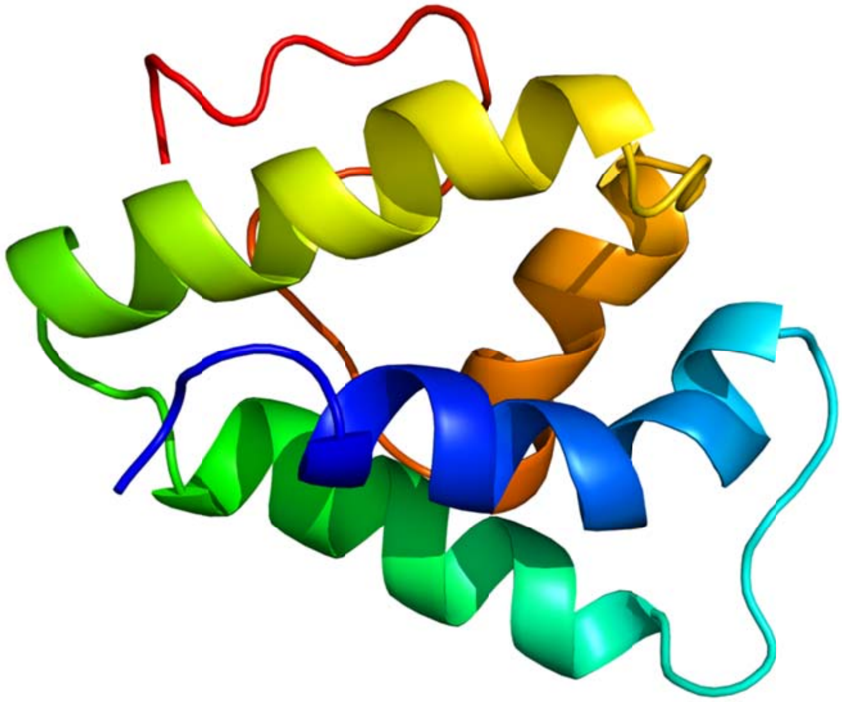

BrnsLtpI.17

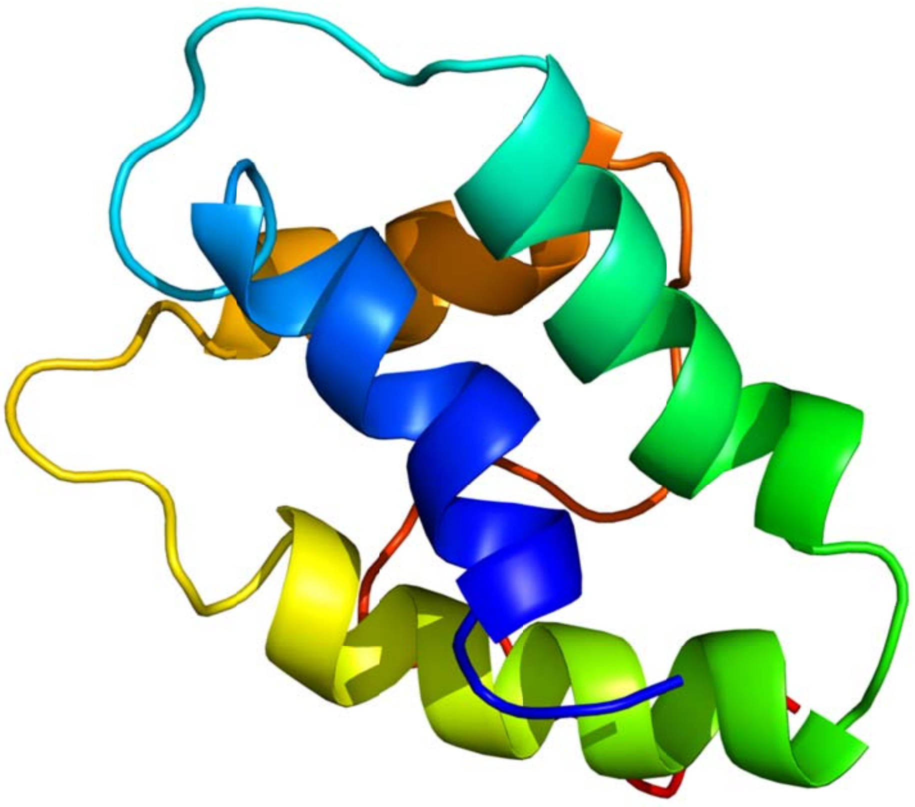

BrnsLtpI.18

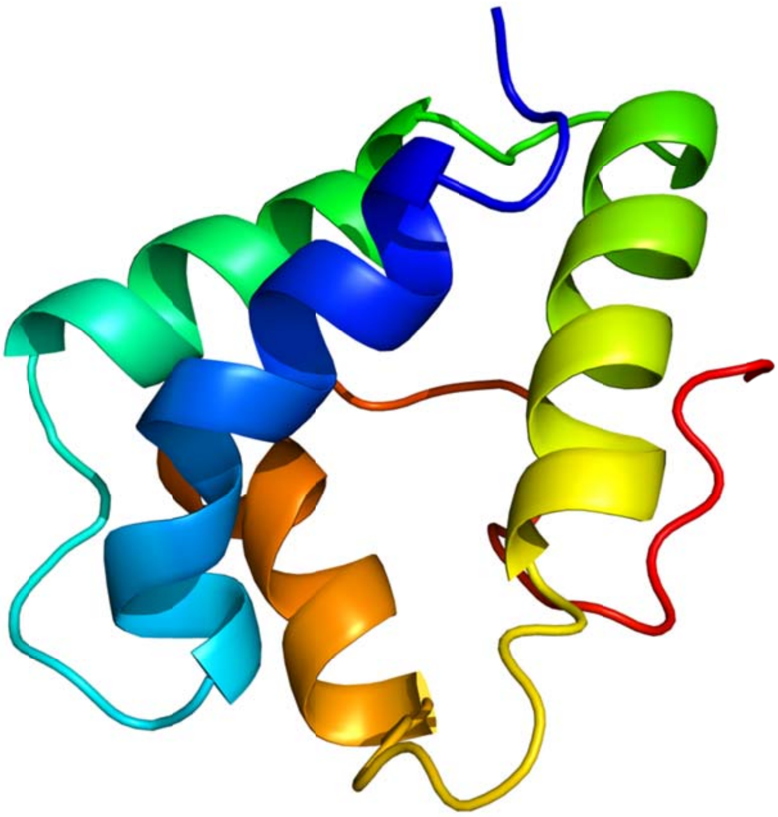

BrnsLtpI.19

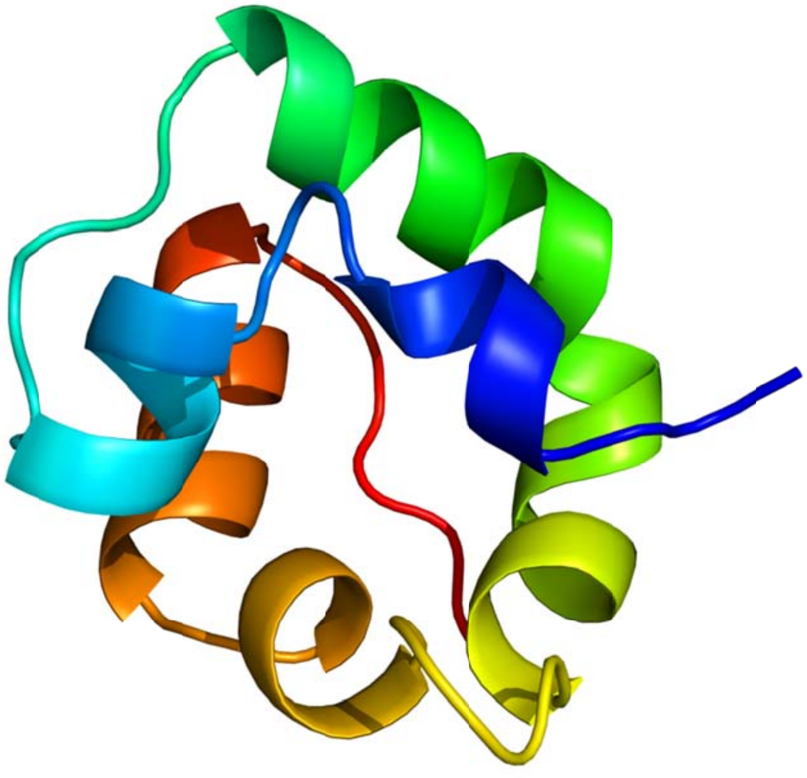

BrnsLtpII.1

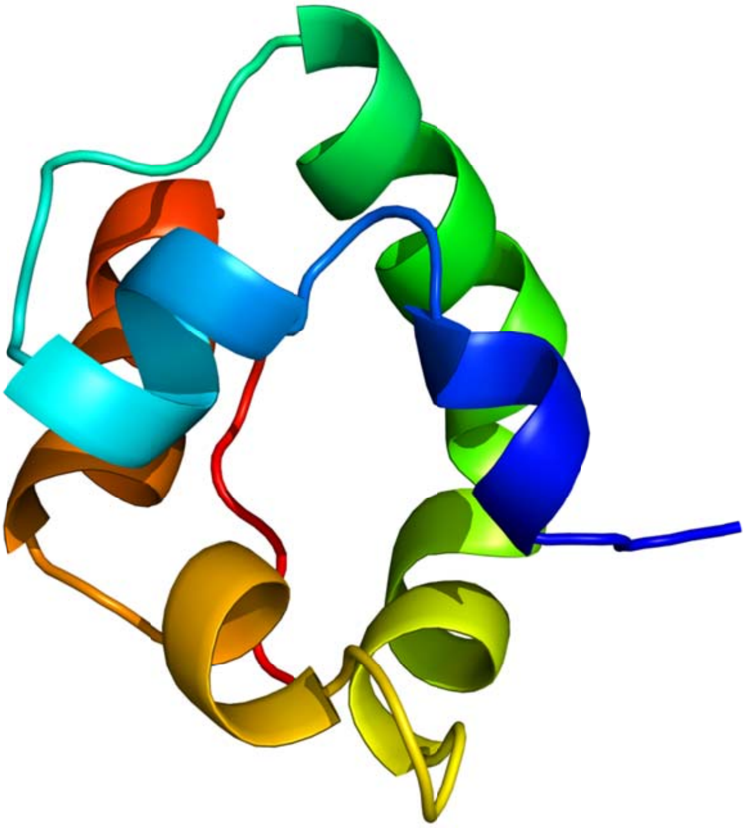

BrnsLtpII.2

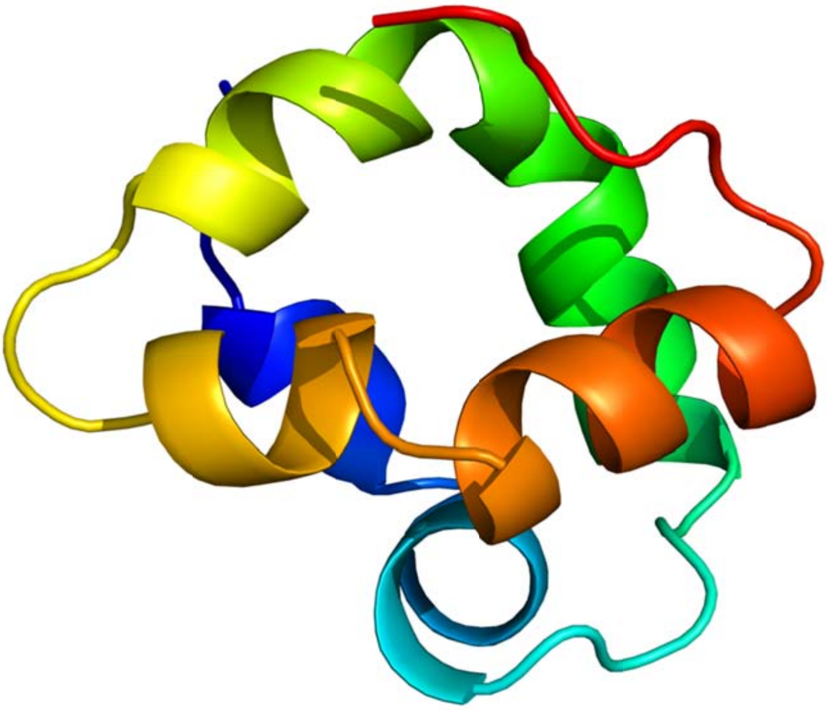

BrnsLtpII.3

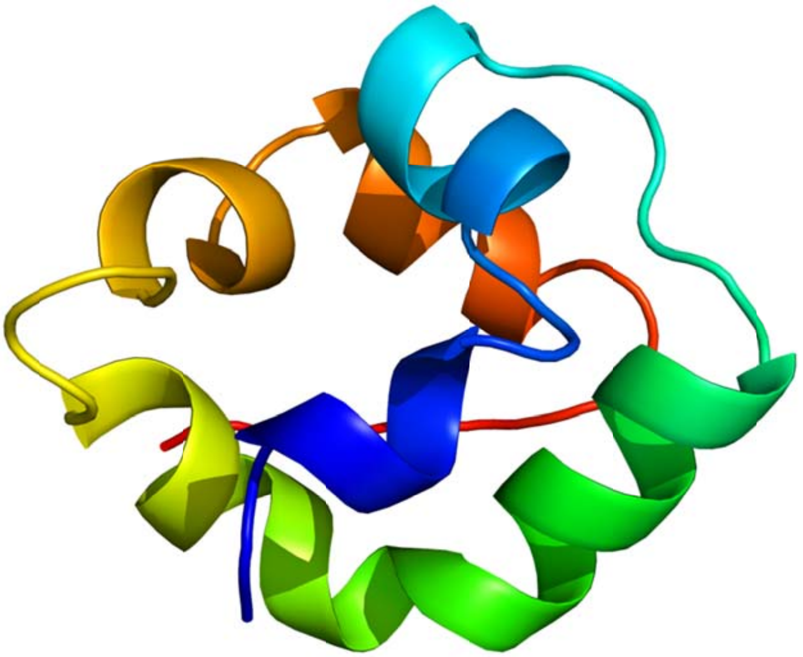

BrnsLtpII.4

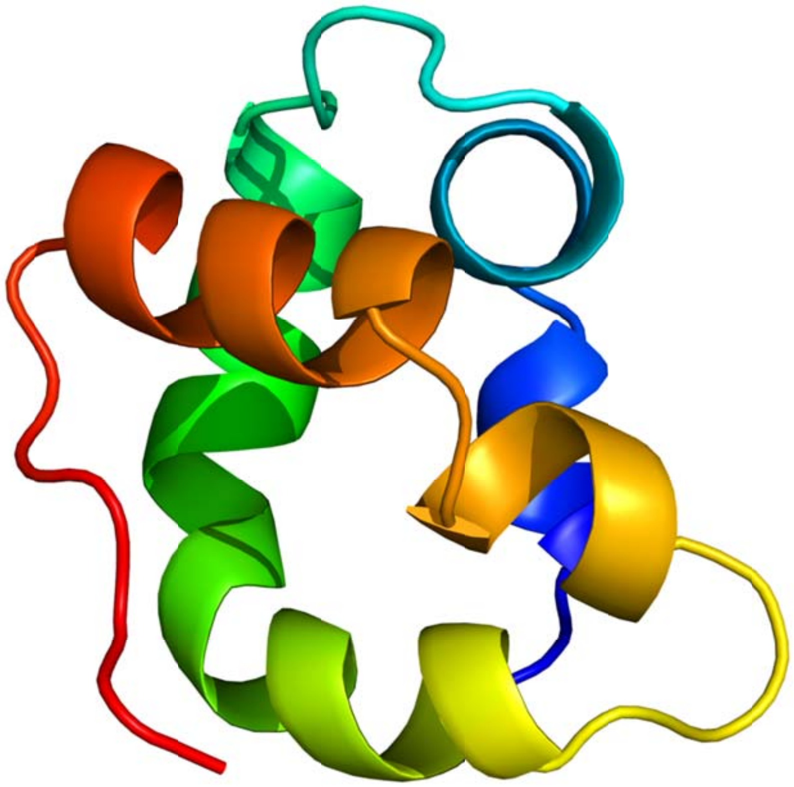

BrnsLtpII.5

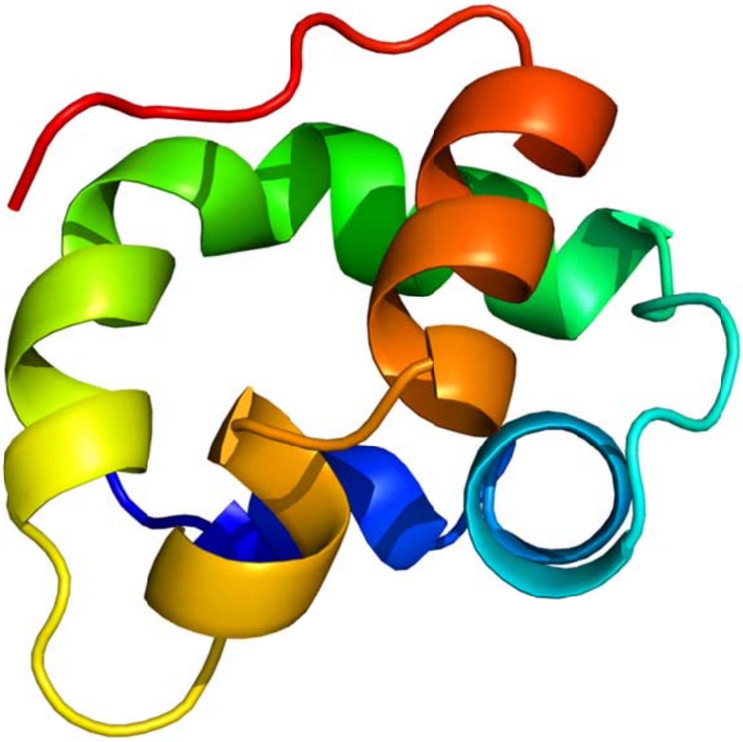

BrnsLtpII.6

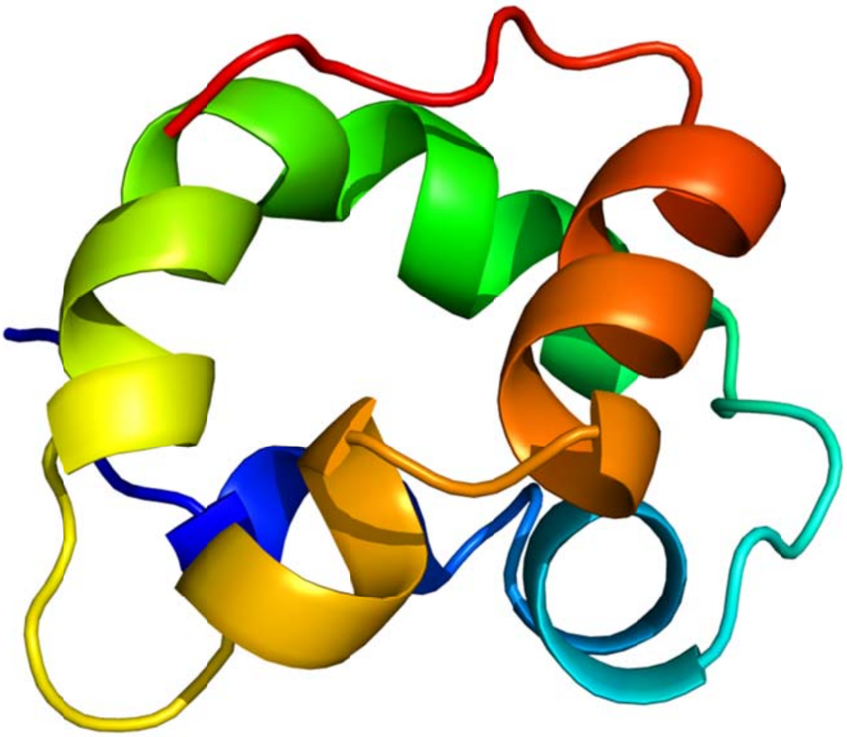

BrnsLtpII.7

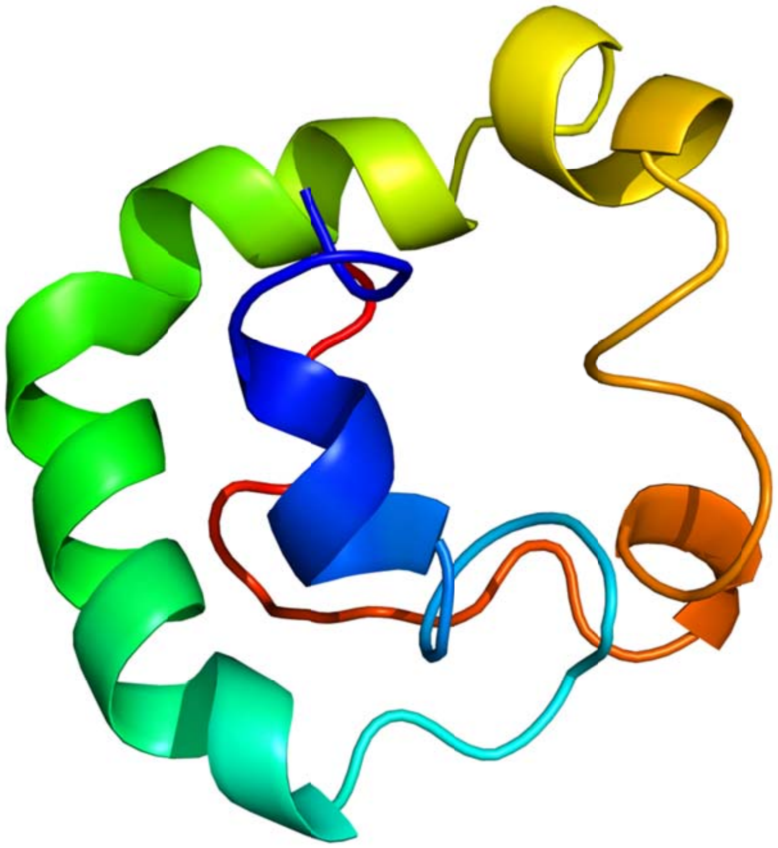

BrnsLtpII.8

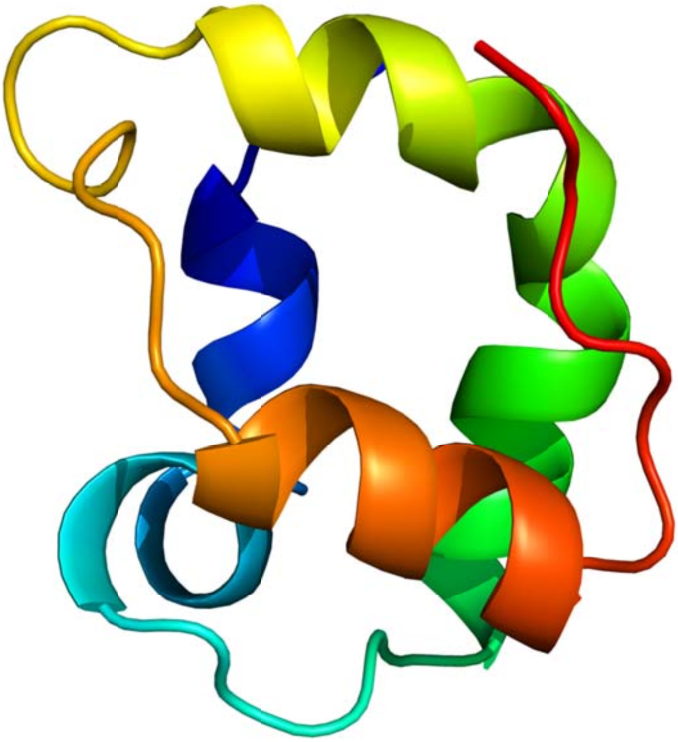

BrnsLtpII.9

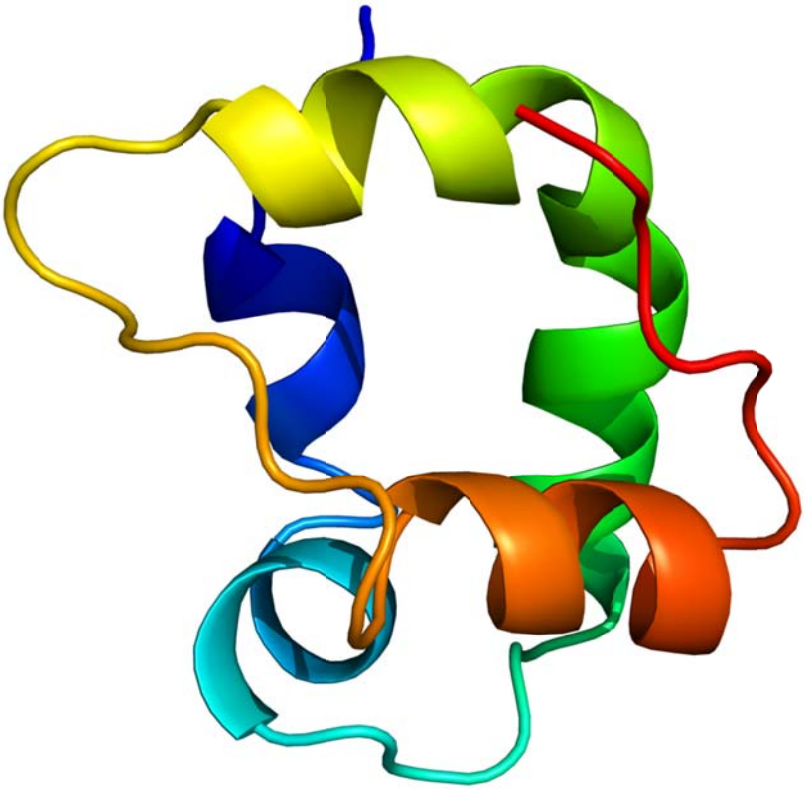

BrnsLtpII.10

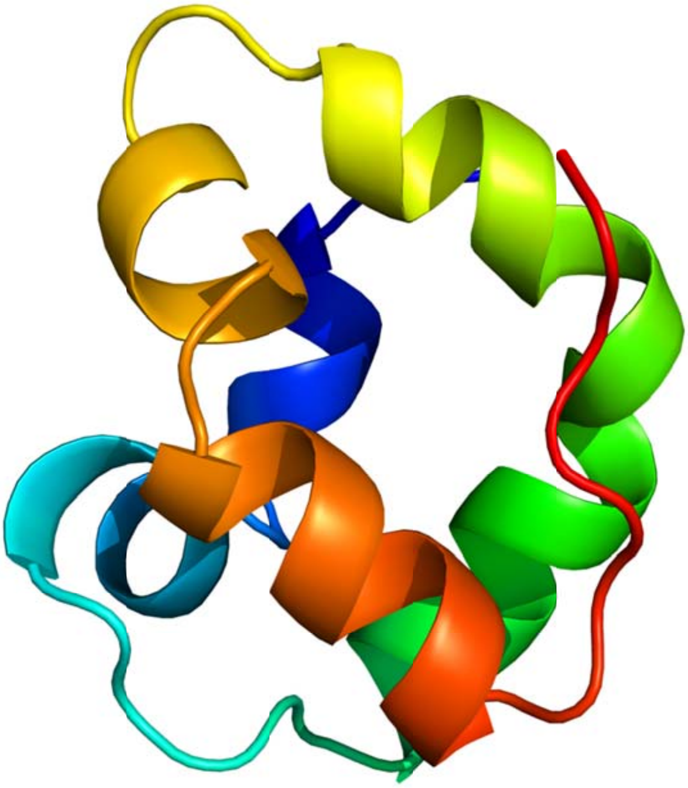

BrnsLtpII.11

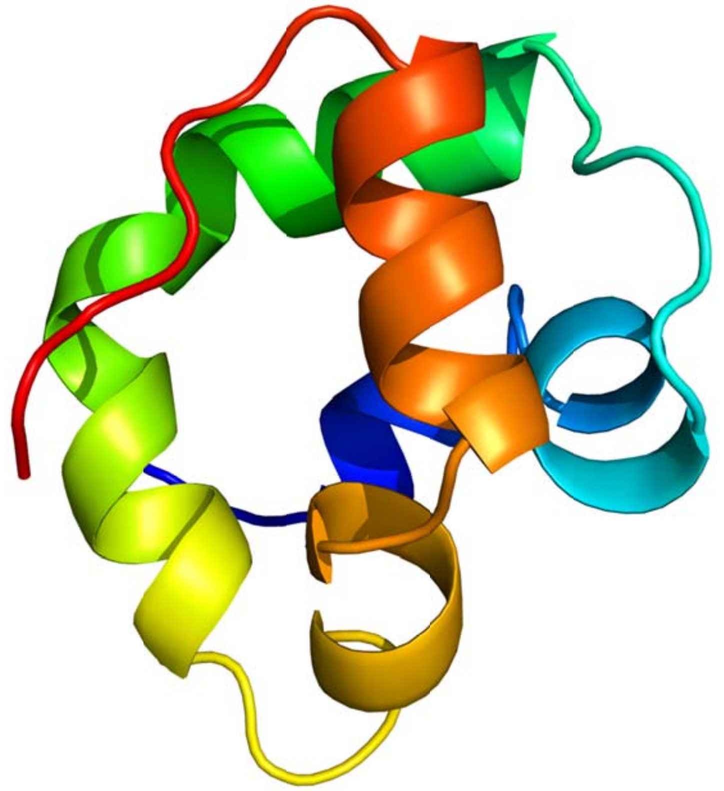

BrnsLtpII.12

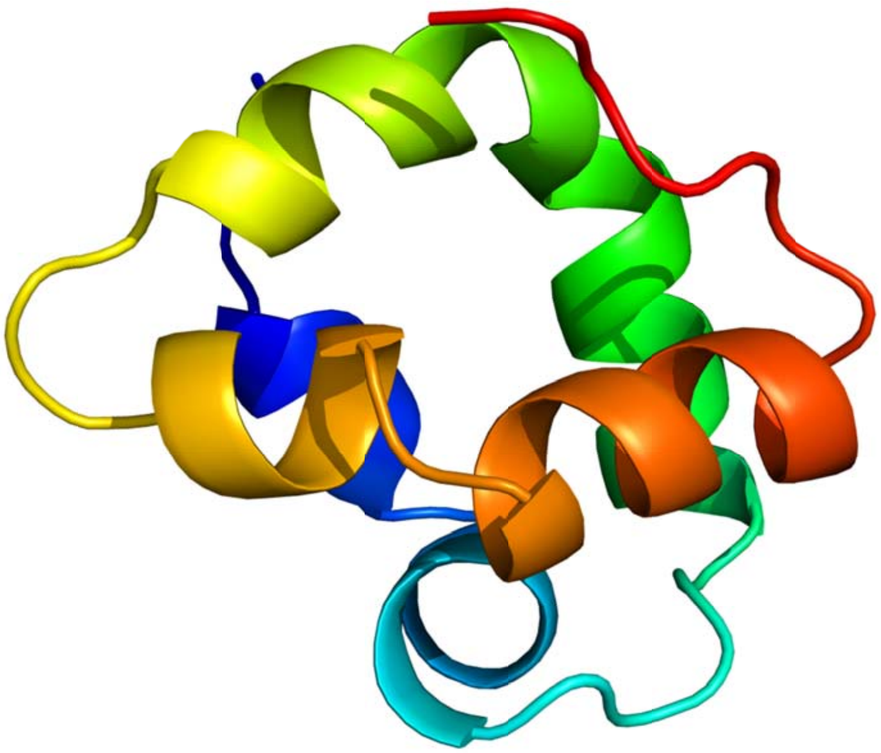

BrnsLtpII.13

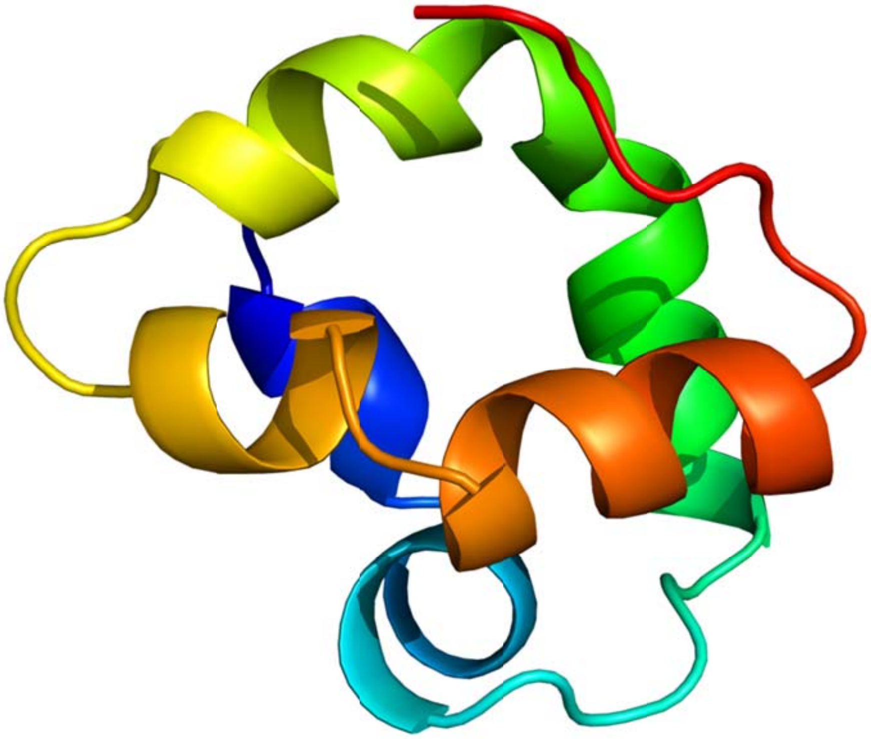

BrnsLtpII.14

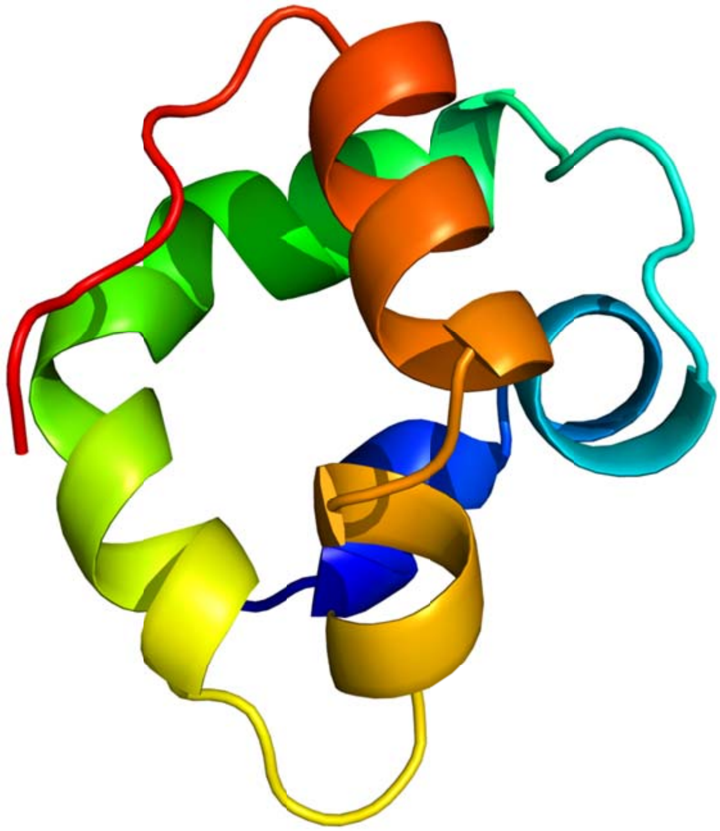

BrnsLtpII.15

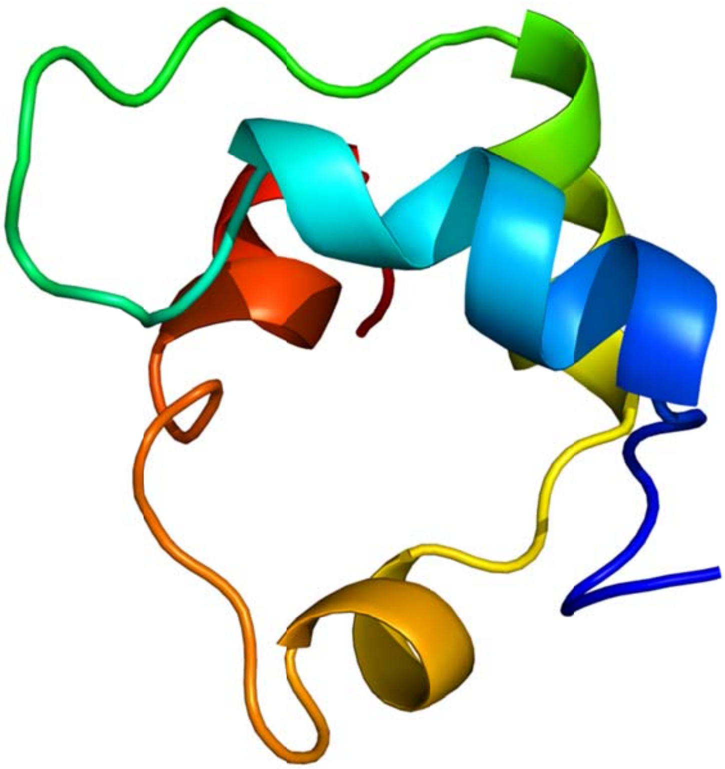

BrnsLtpIII.1

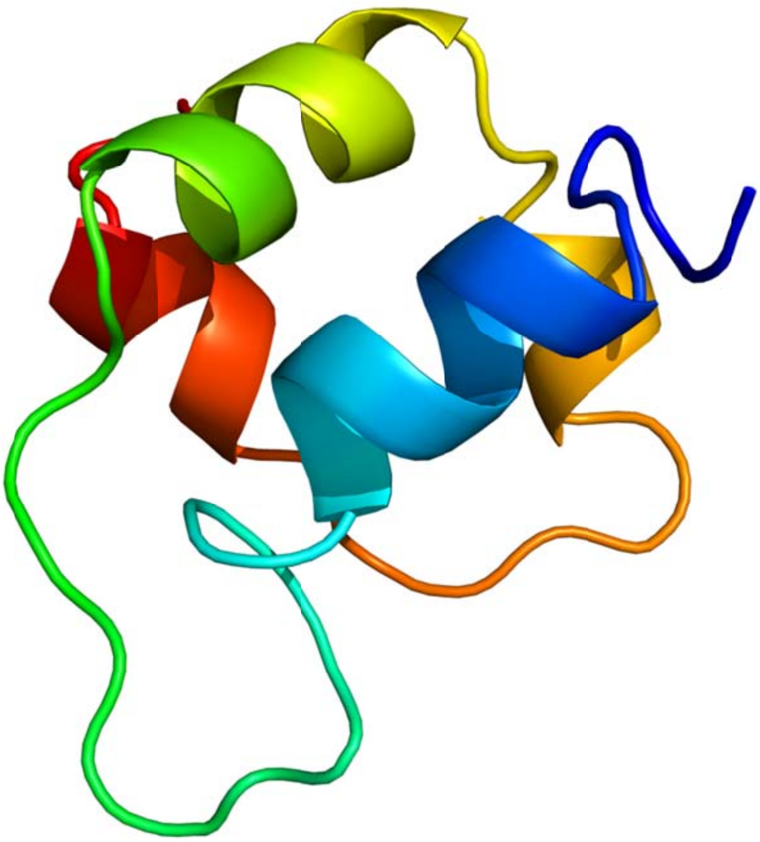

BrnsLtpIII.2

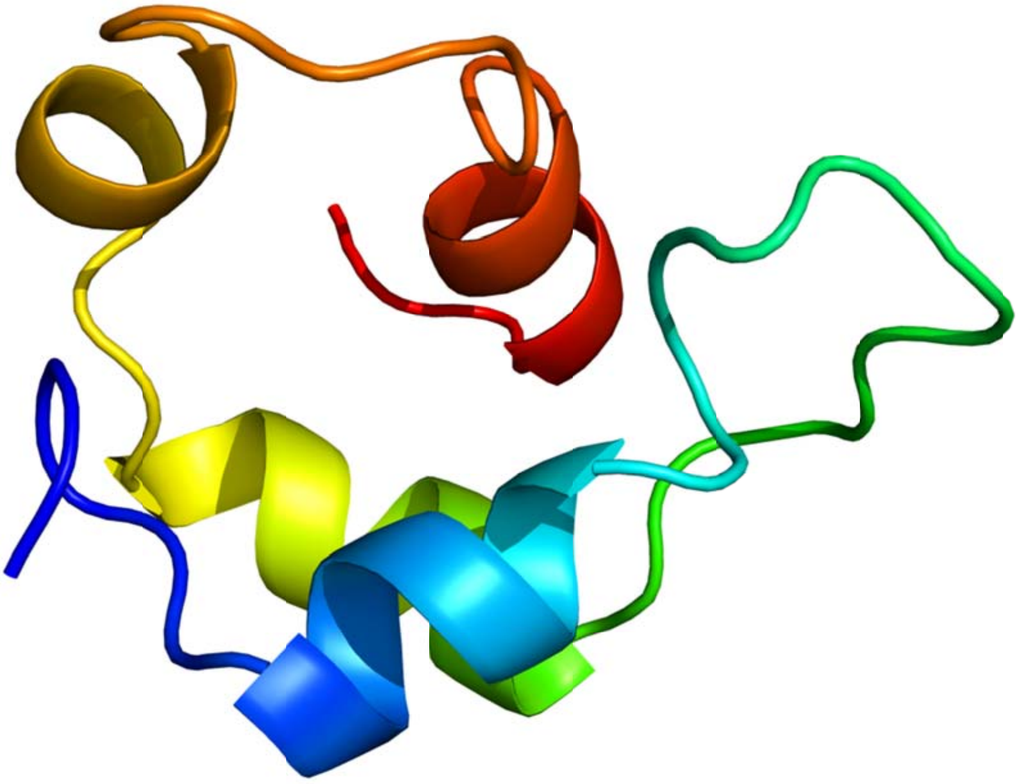

BrnsLtpIII.3

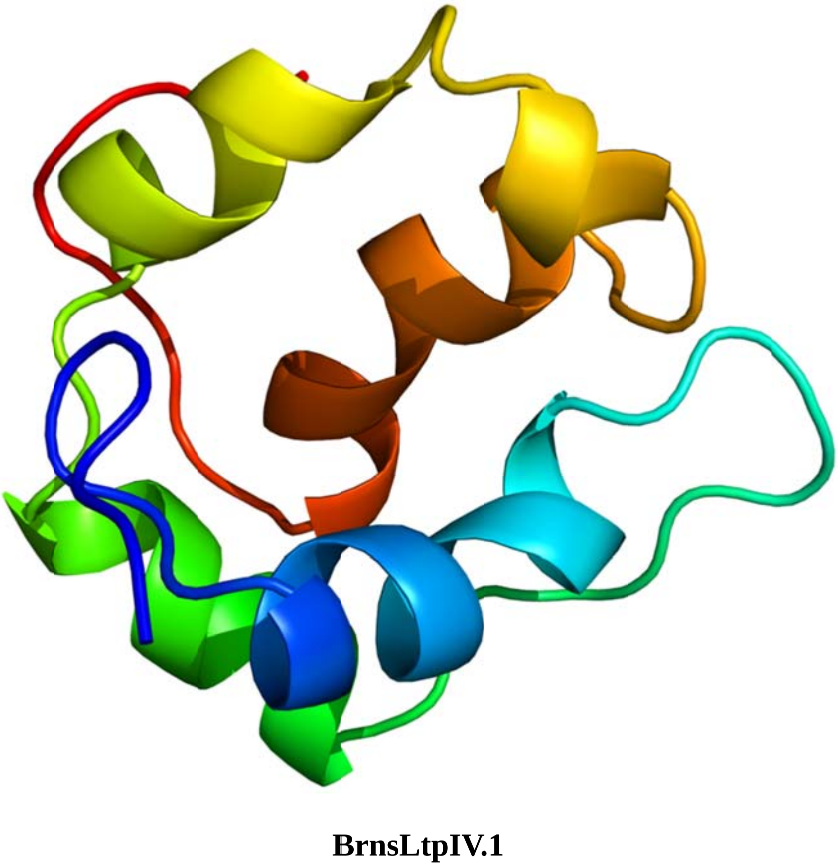

BrnsLtpIV.1

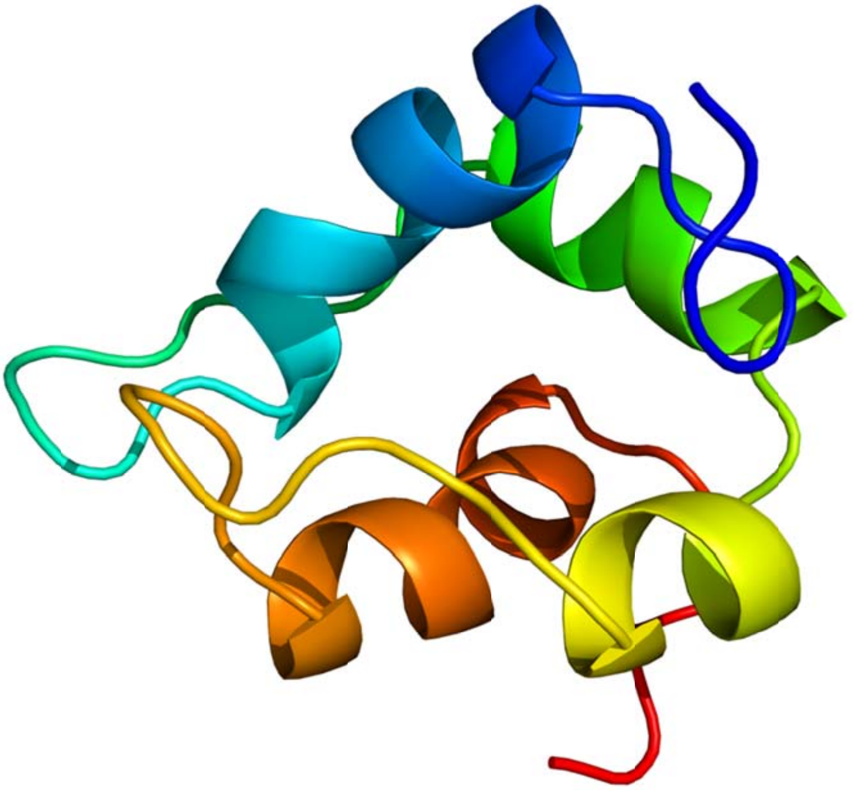

BrnsLtpIV.2

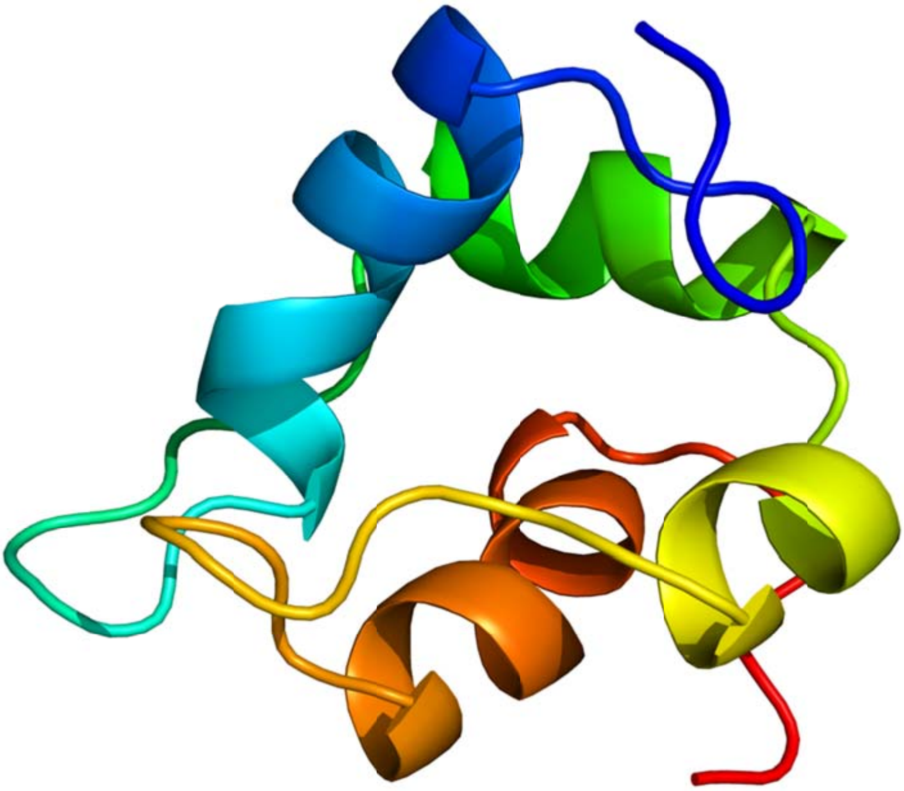

BrnsLtpIV.3

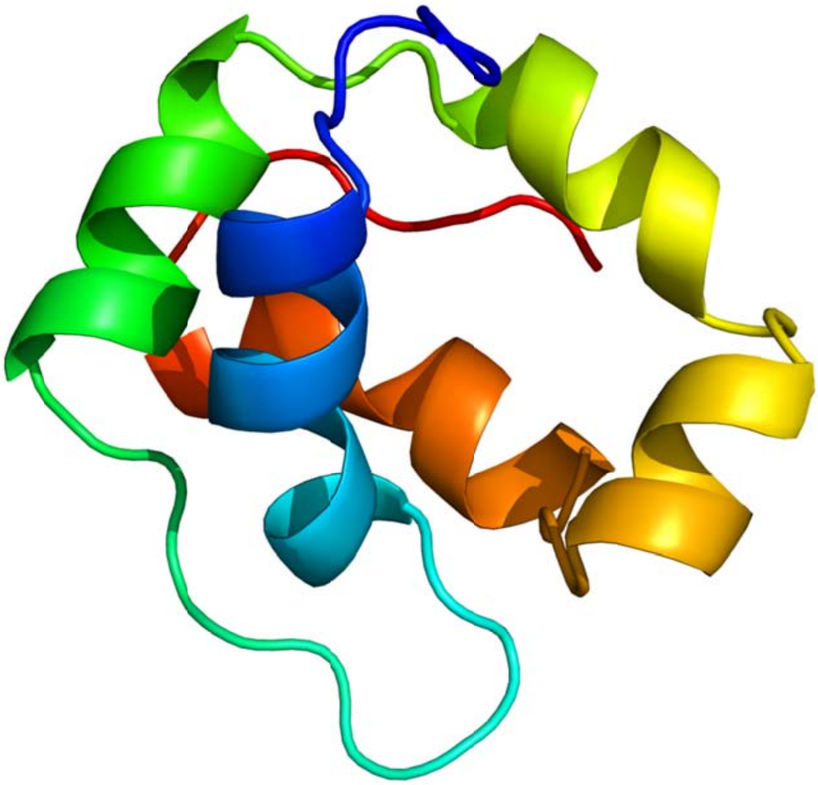

BrnsLtpIV.4

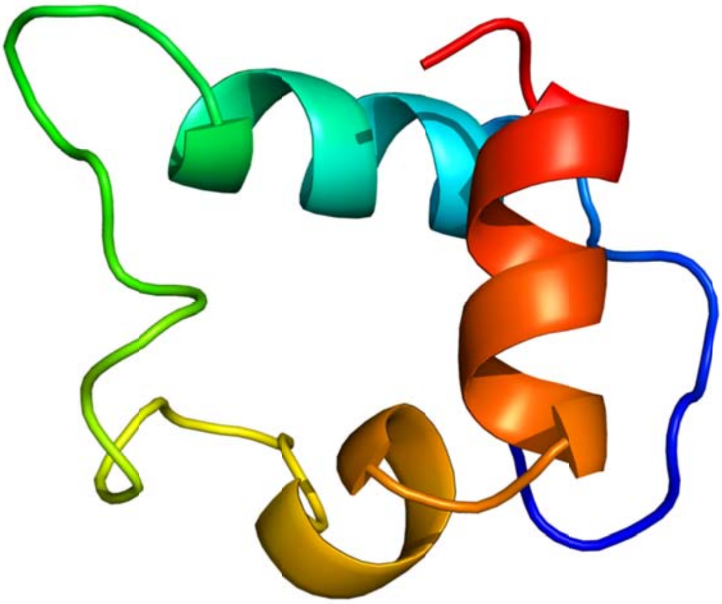

BrnsLtpIV.5

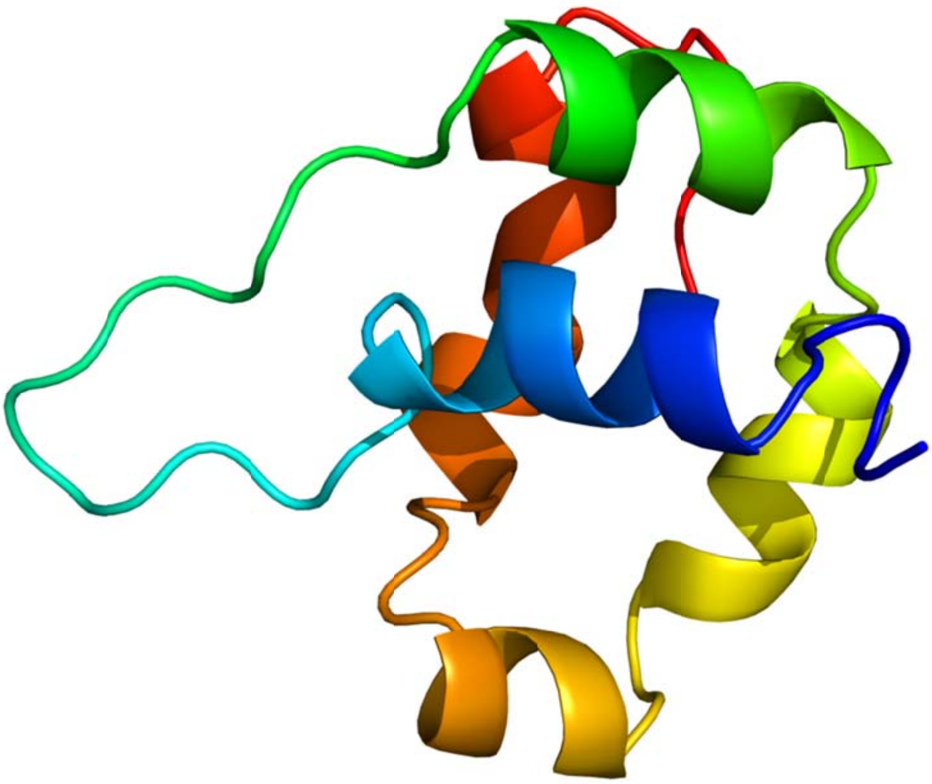

BrnsLtpIV.6

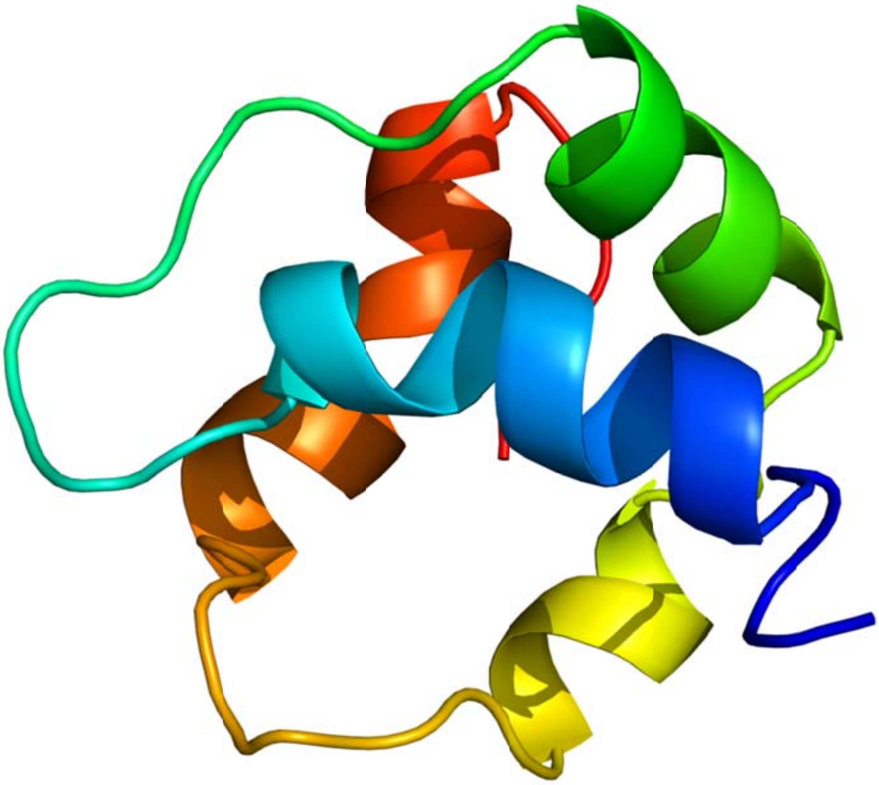

BrnsLtpIV.7

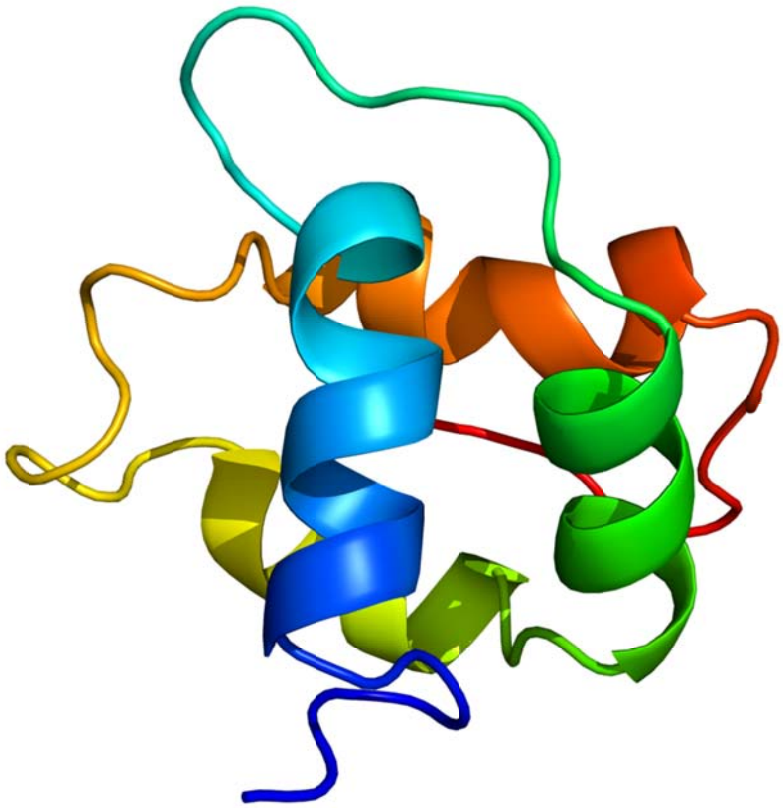

BrnsLtpIV.8

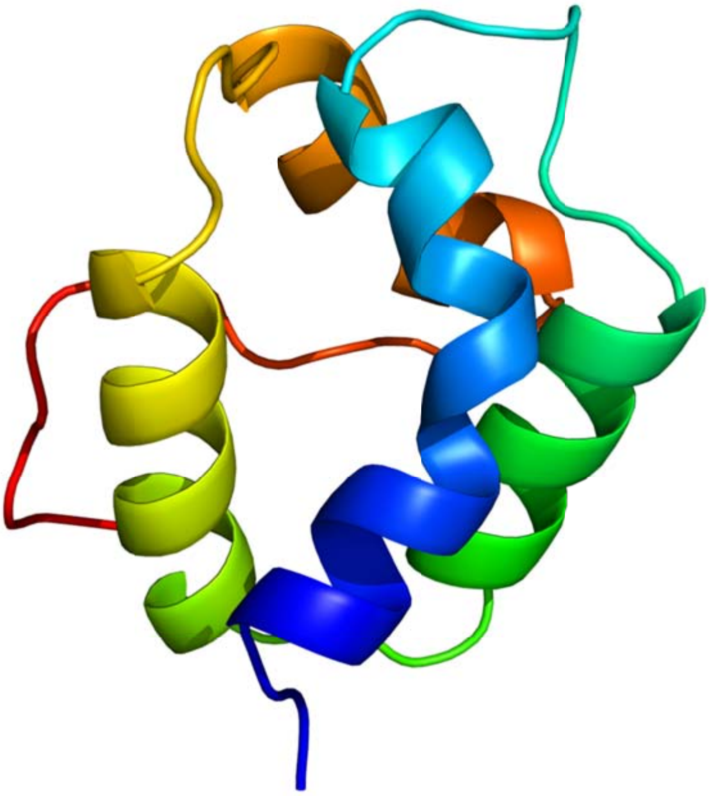

BrnsLtpV.1

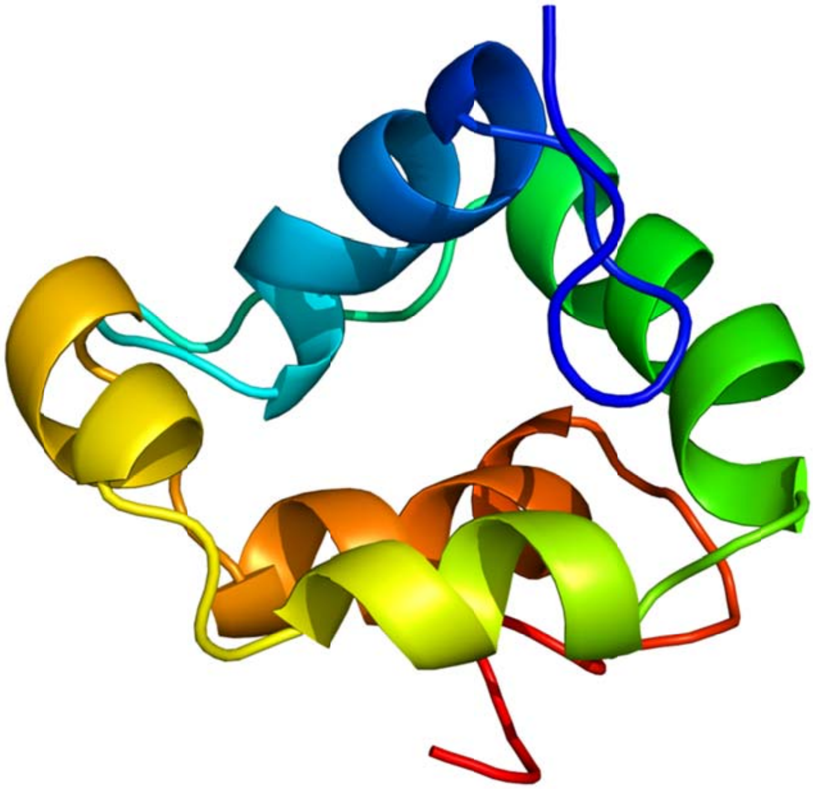

BrnsLtpV.2

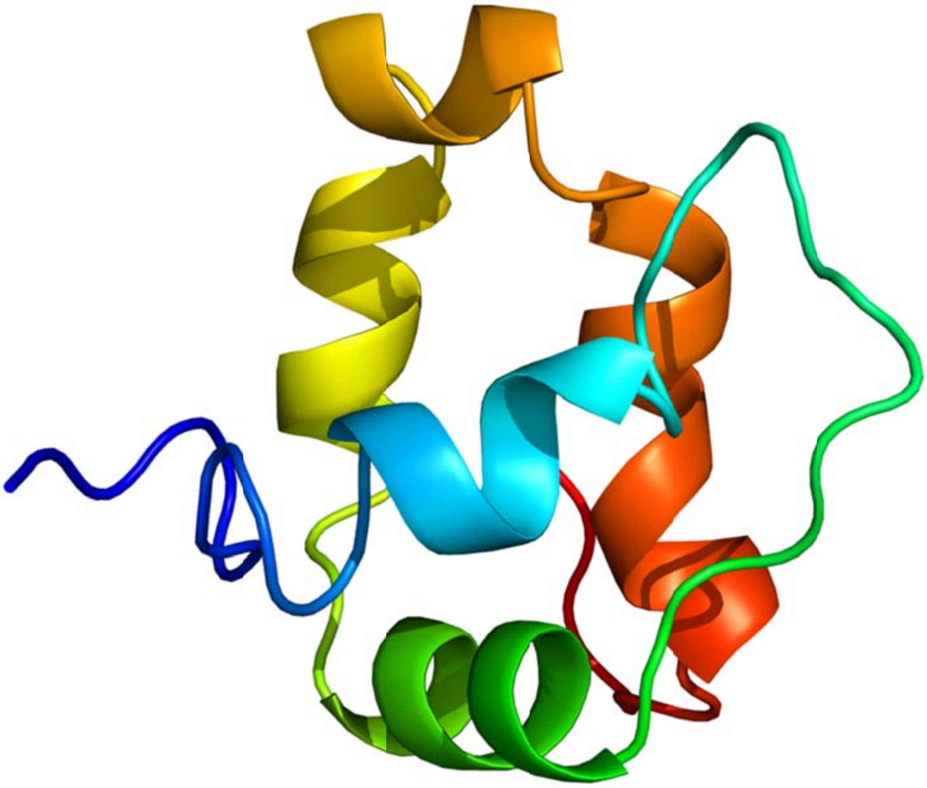

BrnsLtpVI.1

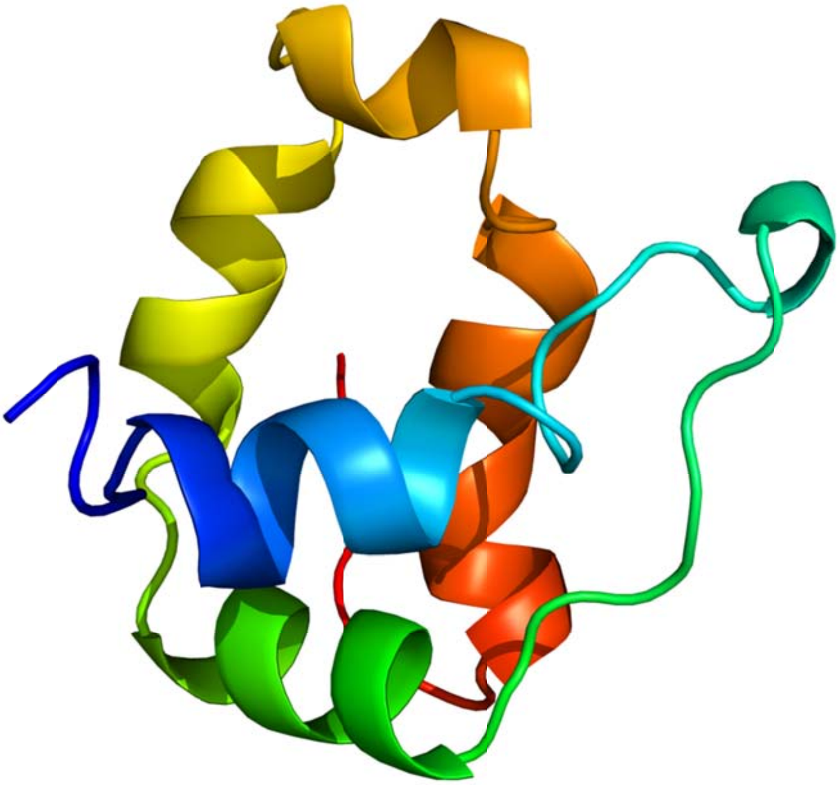

BrnsLtpVI.2

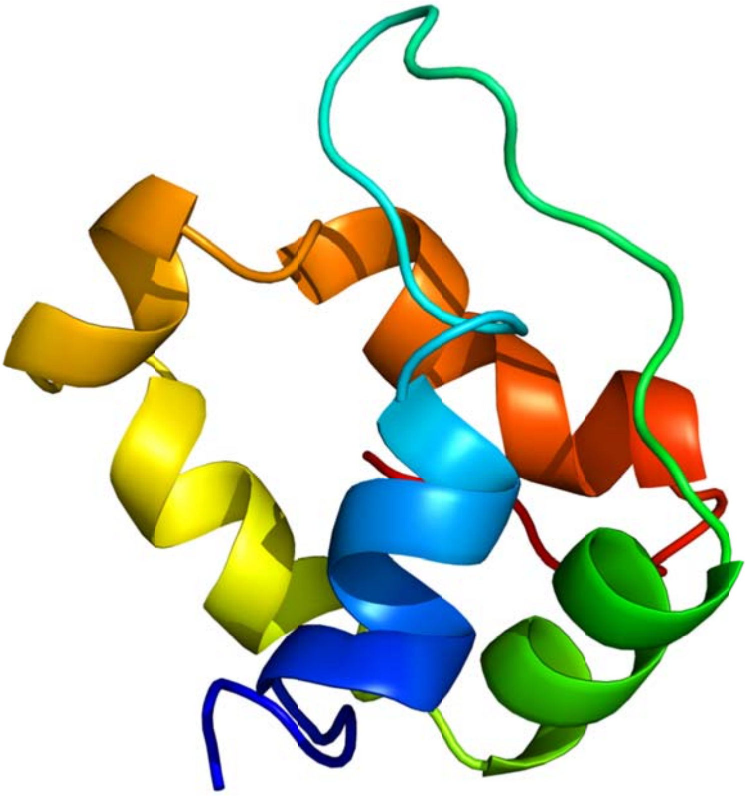

BrnsLtpVI.3

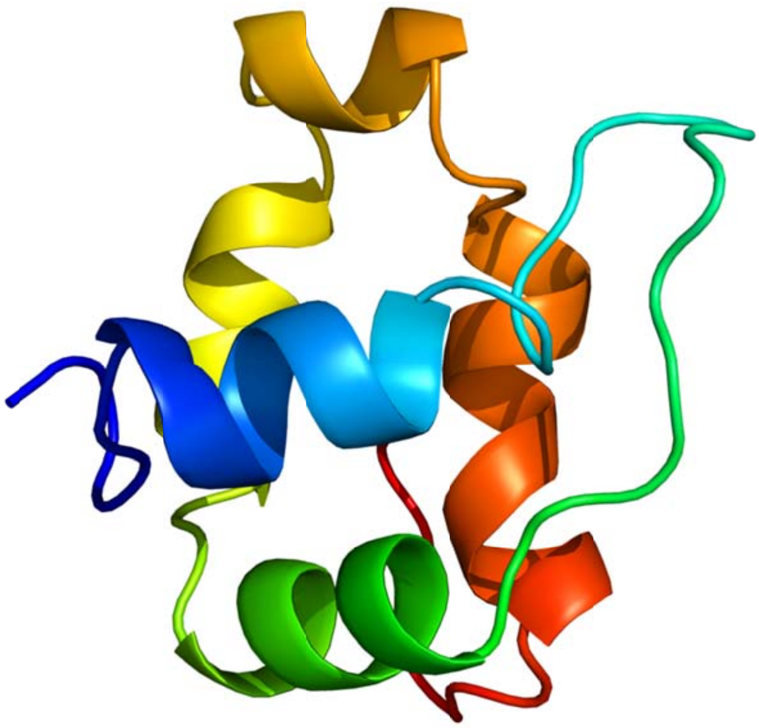

BrnsLtpVI.4

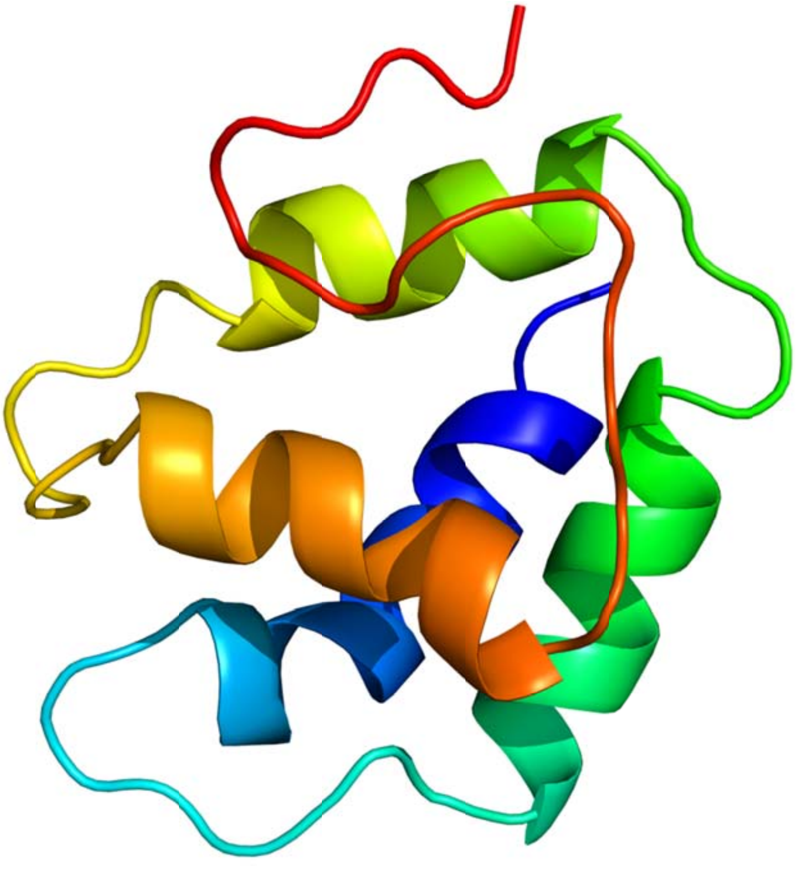

BrnsLtpVIII.1

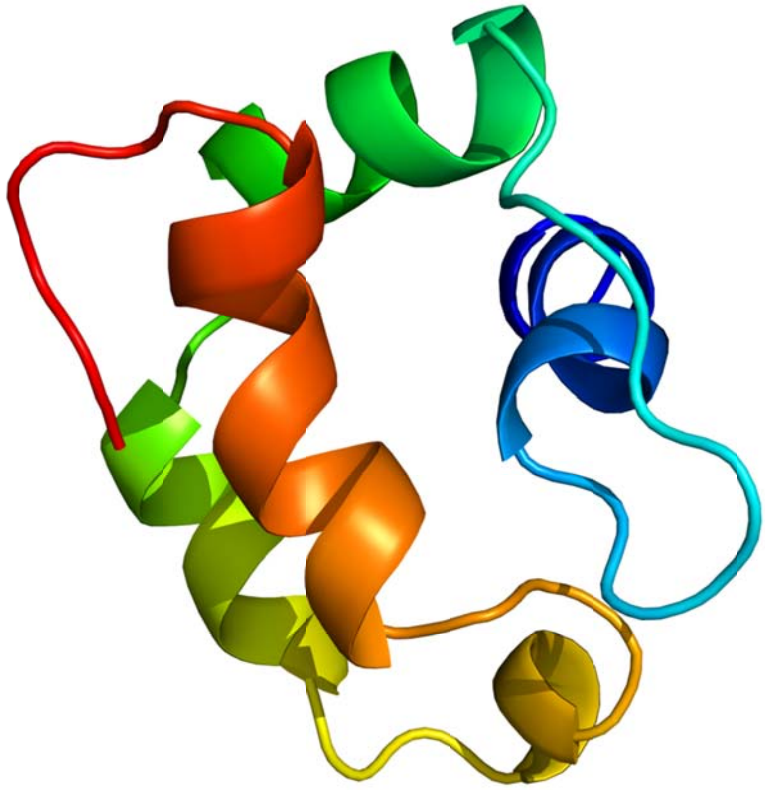

BrnsLtpIX.1

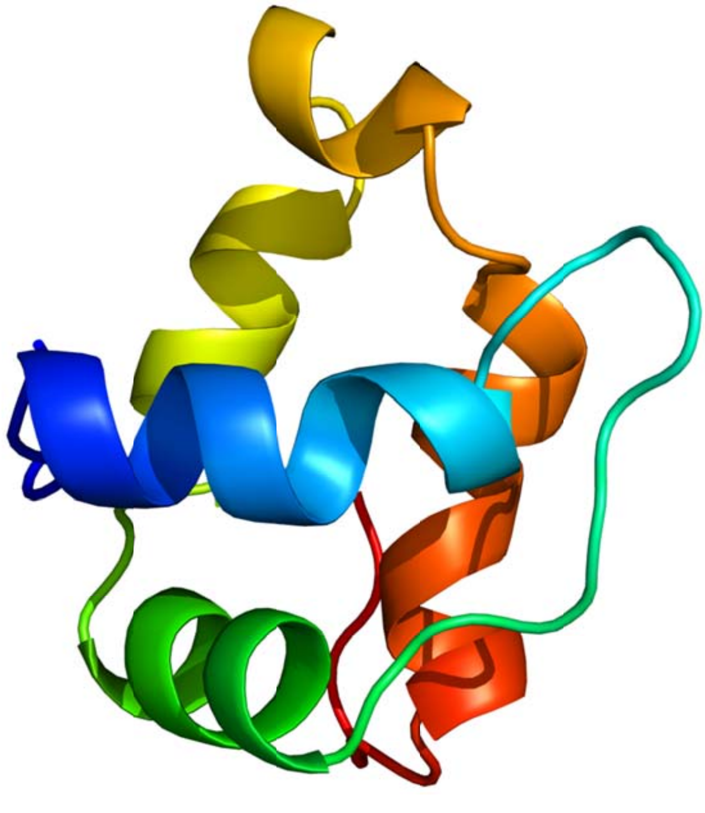

BrnsLtpIX.2

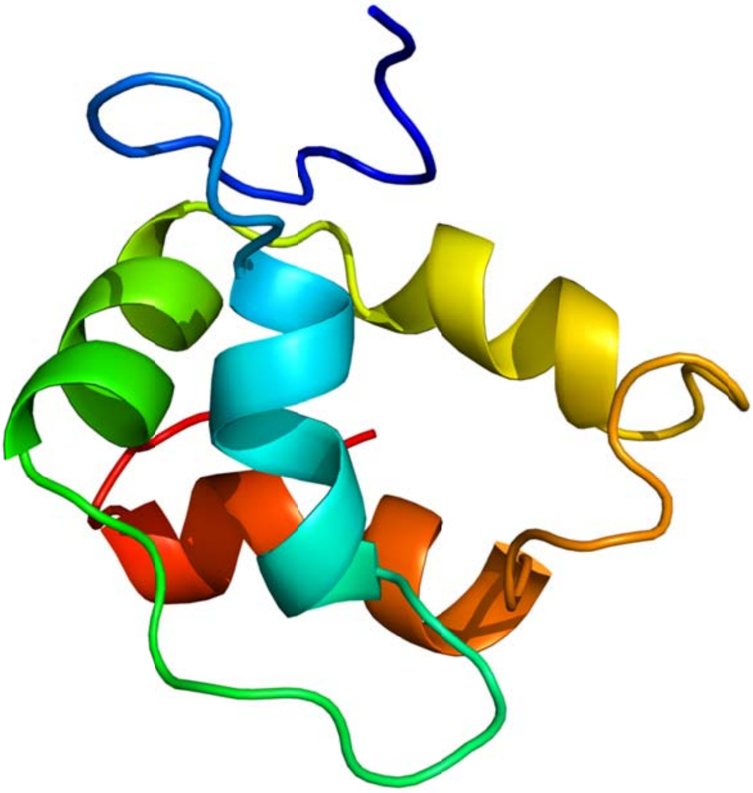

BrnsLtpIX.3

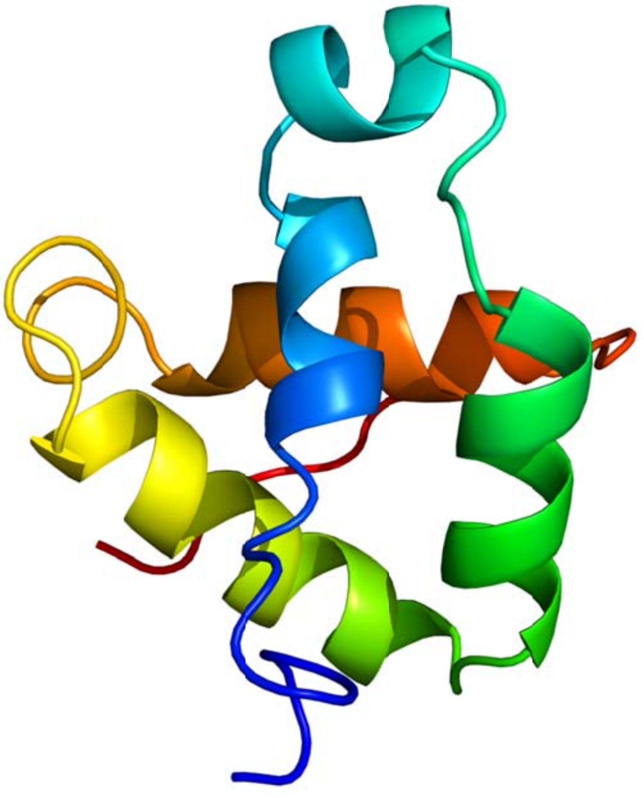

BrnsLtpXI.1

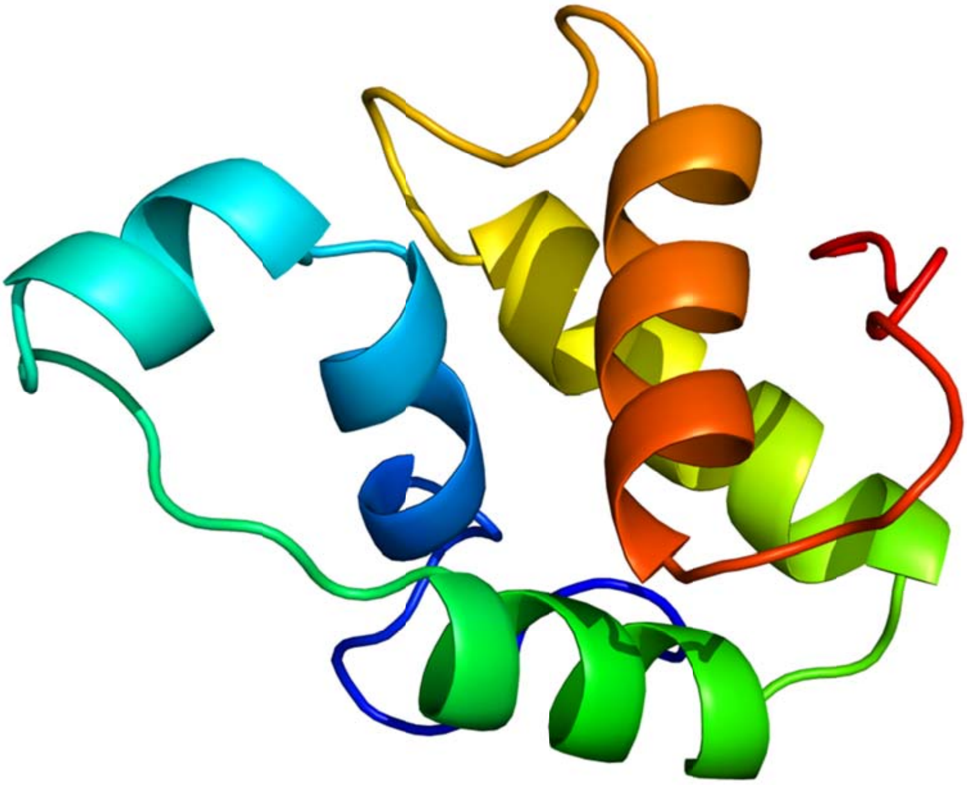

BrnsLtpXI.2

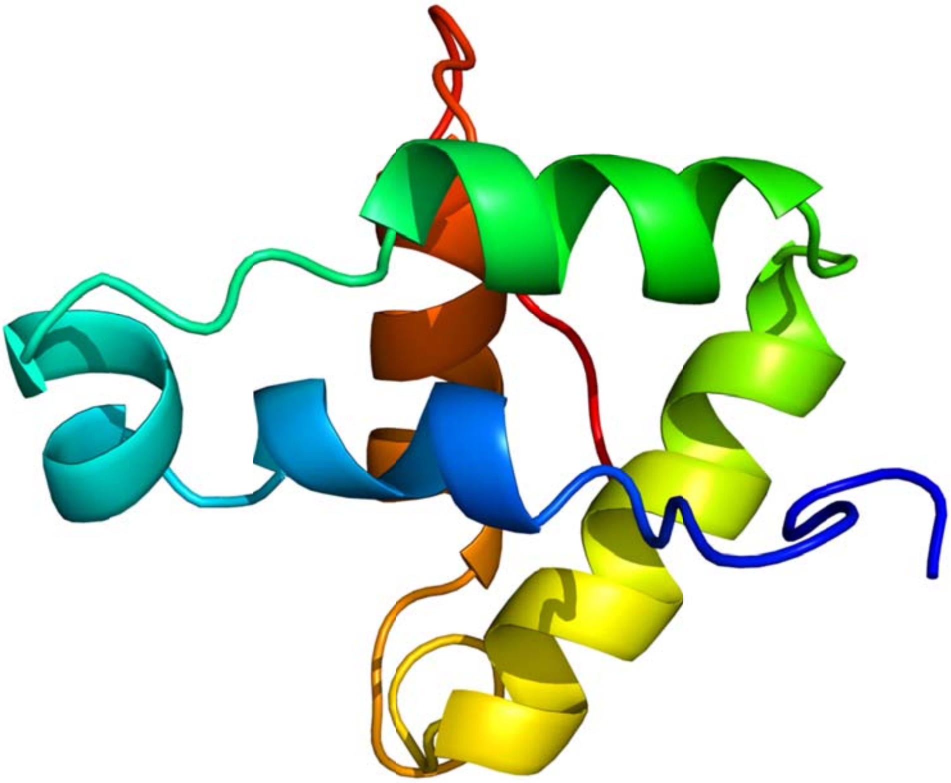

BrnsLtpXI.3

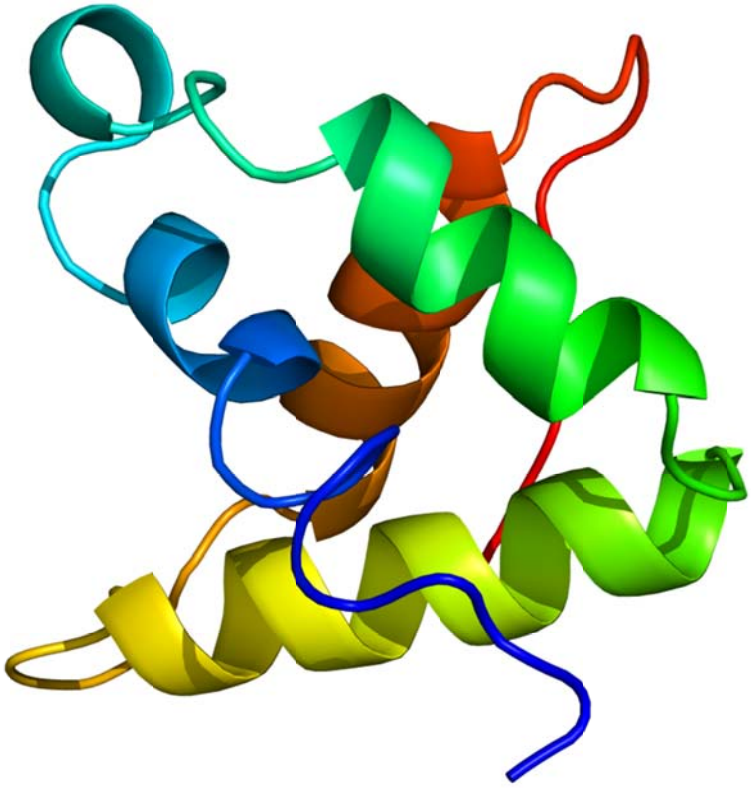

BrnsLtpXI.4

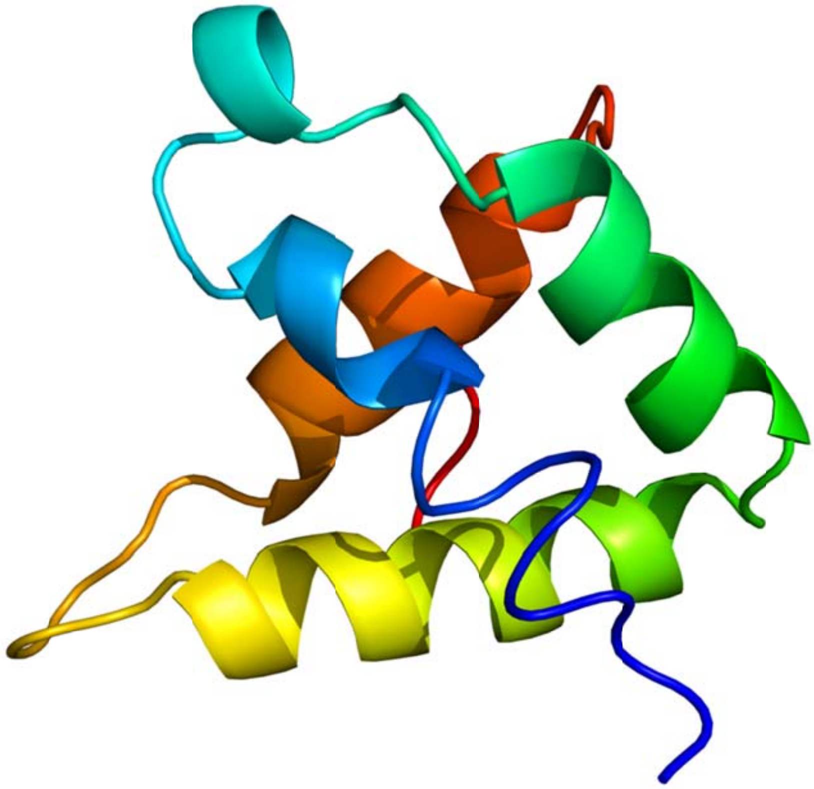

BrnsLtpXI.5

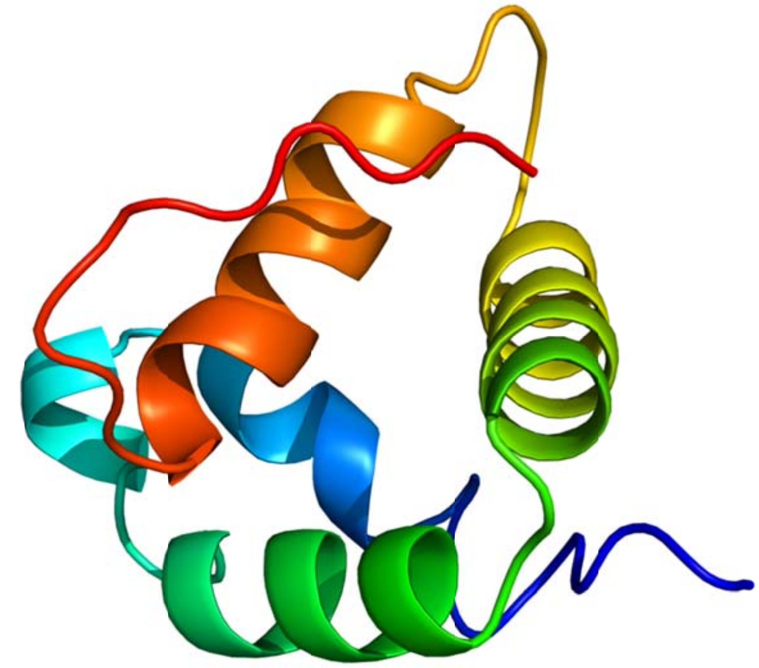

BrnsLtpXI.6

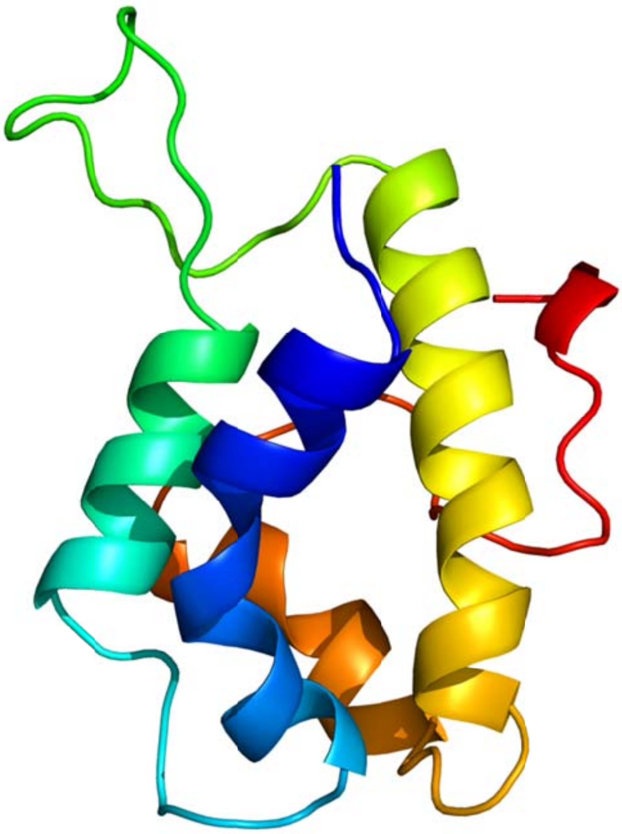

BrnsLtpY.1

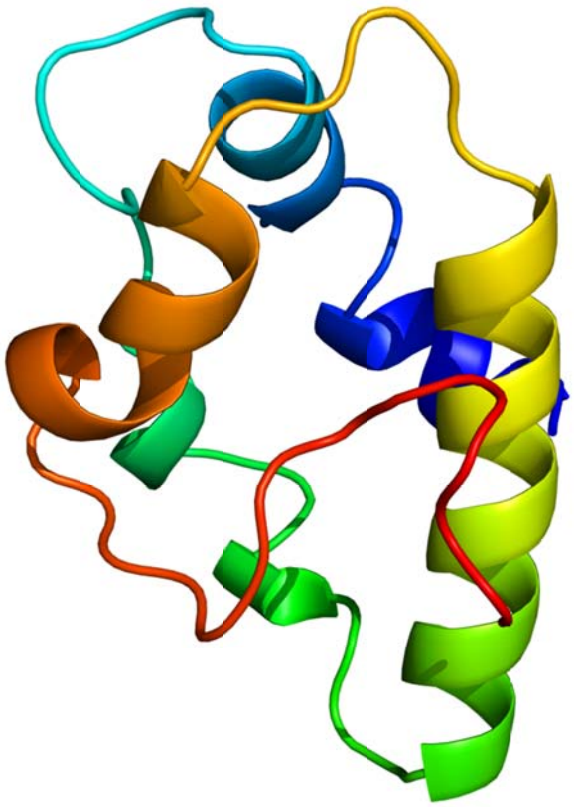

BrnsLtpY.2
