# Supplementary material for: Genome-Wide Survey and Expression Analysis of the Putative Non-Specific Lipid Transfer Proteins in Brassica rapa L
Source: PLoS One. 2014 Jan 31;9(1):e84556. doi: 10.1371/journal.pone.0084556 (PMC3908880; doi:10.1371/journal.pone.0084556)
Supplement: Figure S3 — Schematic representation of the primers used in quantitative RT-PCR analysis. (PDF) [file pone.0084556.s003.pdf]

Figure S3. Schematic representation of primers used in quantitative RT-PCR analysis.

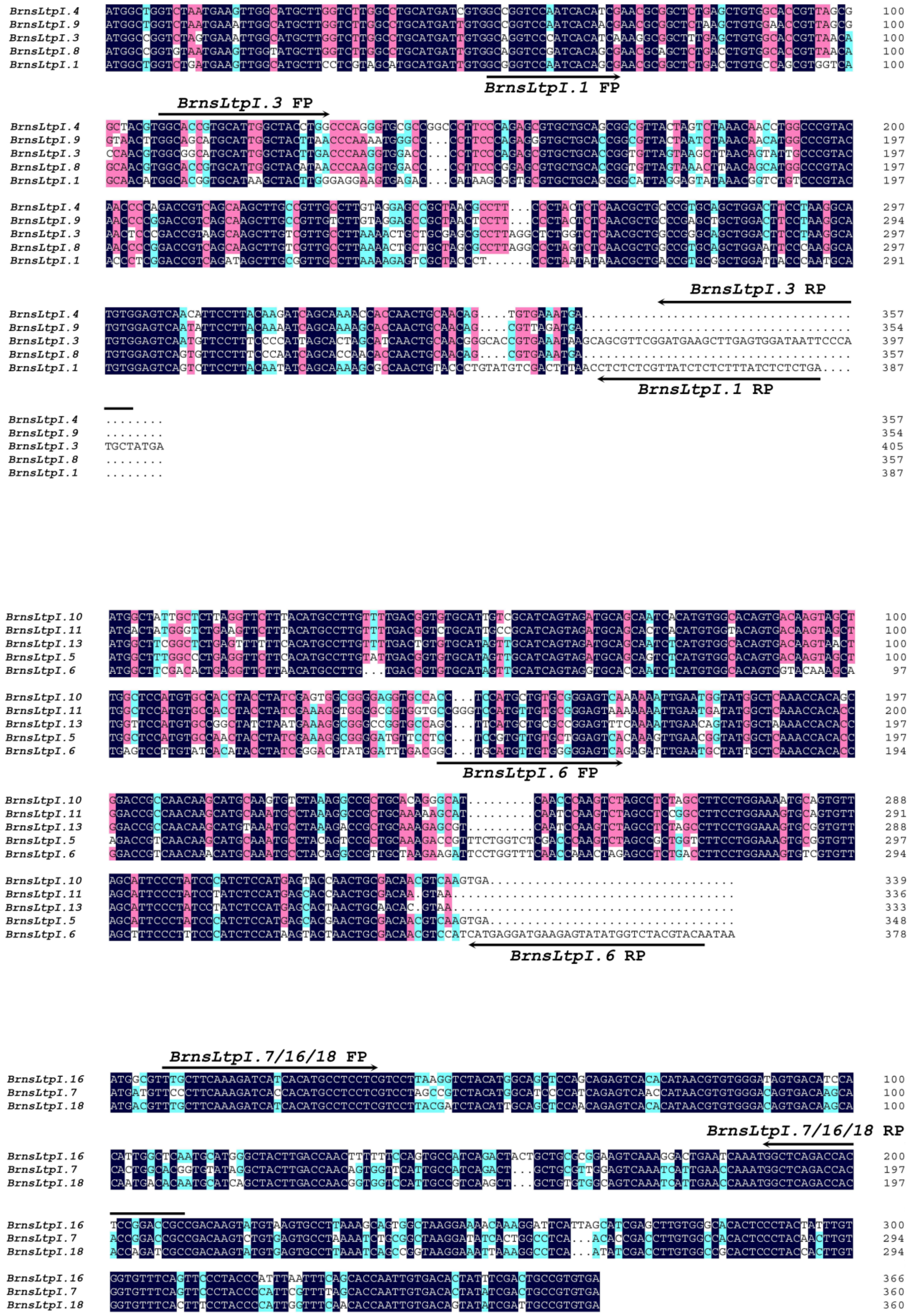

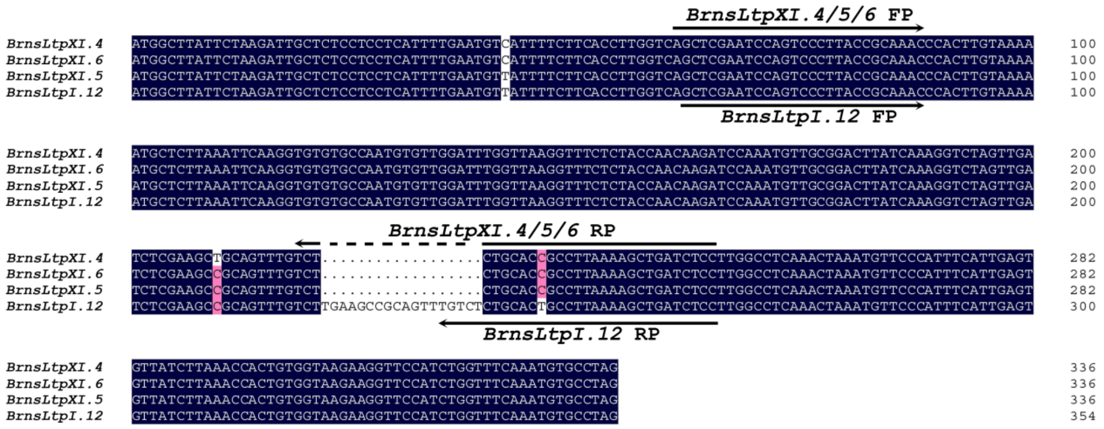
BrnsLtpXI.4/5/6 FP
BrnsLtpXI.4
BrnsLtpXI.6
BrnsLtpXI.5
BrnsLtpI.12
BrnsLtpI.12 FP
BrnsLtpXI.4/5/6 RP
BrnsLtpI.12 RP

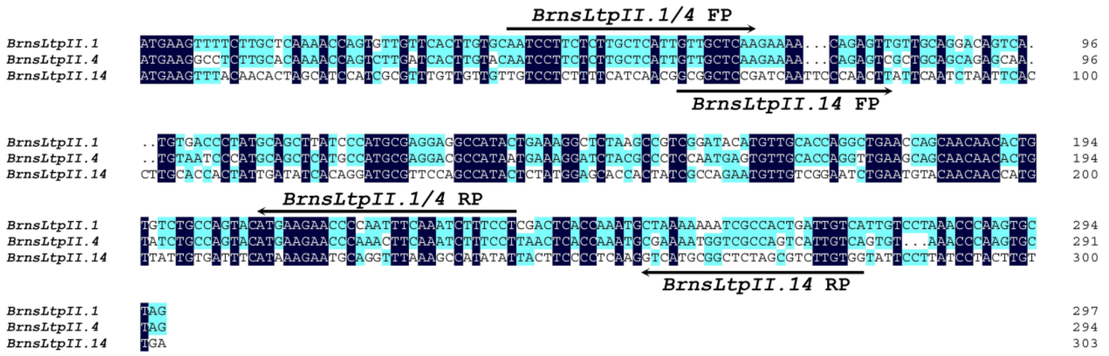
BrnsLtpII.1/4 FP
BrnsLtpII.1
BrnsLtpII.4
BrnsLtpII.14
BrnsLtpII.14 FP
BrnsLtpII.1/4 RP
BrnsLtpII.14 RP

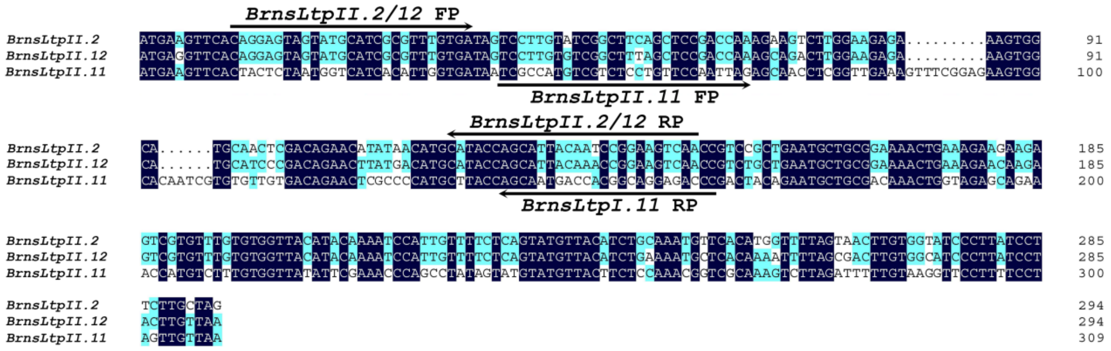
BrnsLtpII.2/12 FP
BrnsLtpII.2
BrnsLtpII.12
BrnsLtpII.11
BrnsLtpII.11 FP
BrnsLtpII.2/12 RP
BrnsLtpI.11 RP

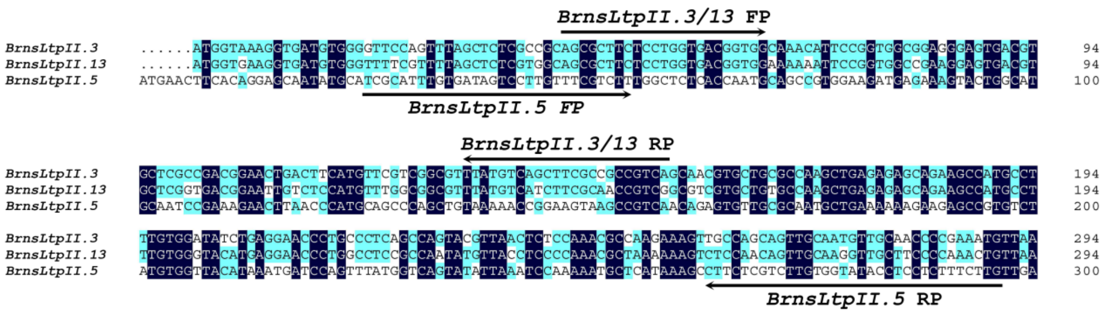
BrnsLtpII.3/13 FP
BrnsLtpII.3
BrnsLtpII.13
BrnsLtpII.5
BrnsLtpII.5 FP
BrnsLtpII.3/13 RP
BrnsLtpII.5 RP

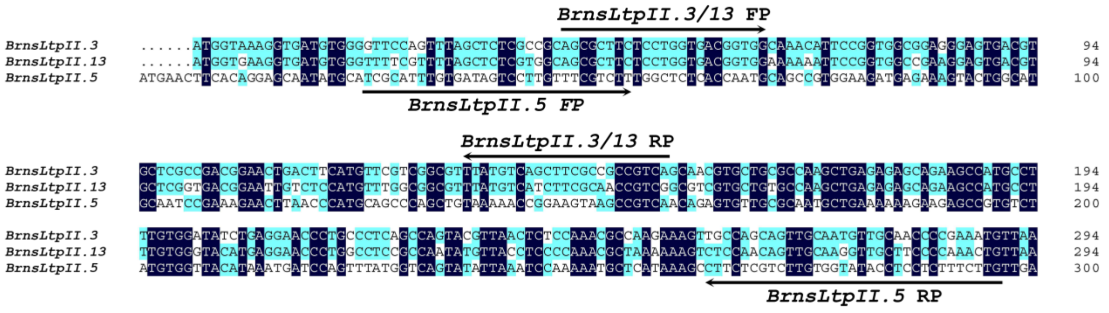
BrnsLtpII.3/13 FP
BrnsLtpII.3
BrnsLtpII.13
BrnsLtpII.5
BrnsLtpII.5 FP
BrnsLtpII.3/13 RP
BrnsLtpII.5 RP

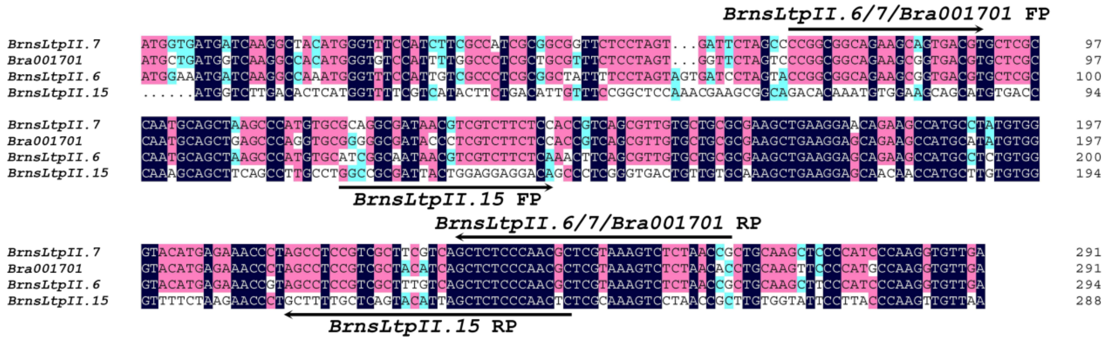
BrnsLtpII.6/7/Bra001701 FP
BrnsLtpII.7
Bra001701
BrnsLtpII.6
BrnsLtpII.15
BrnsLtpII.15 FP
BrnsLtpII.6/7/Bra001701 RP
BrnsLtpII.15 RP

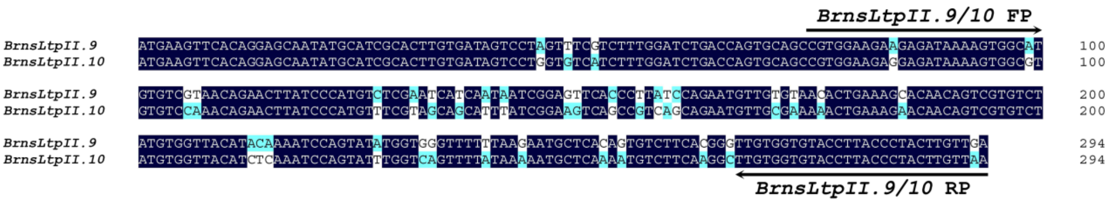
BrnsLtpII.9/10 FP
BrnsLtpII.9
BrnsLtpII.10
BrnsLtpII.9/10 RP

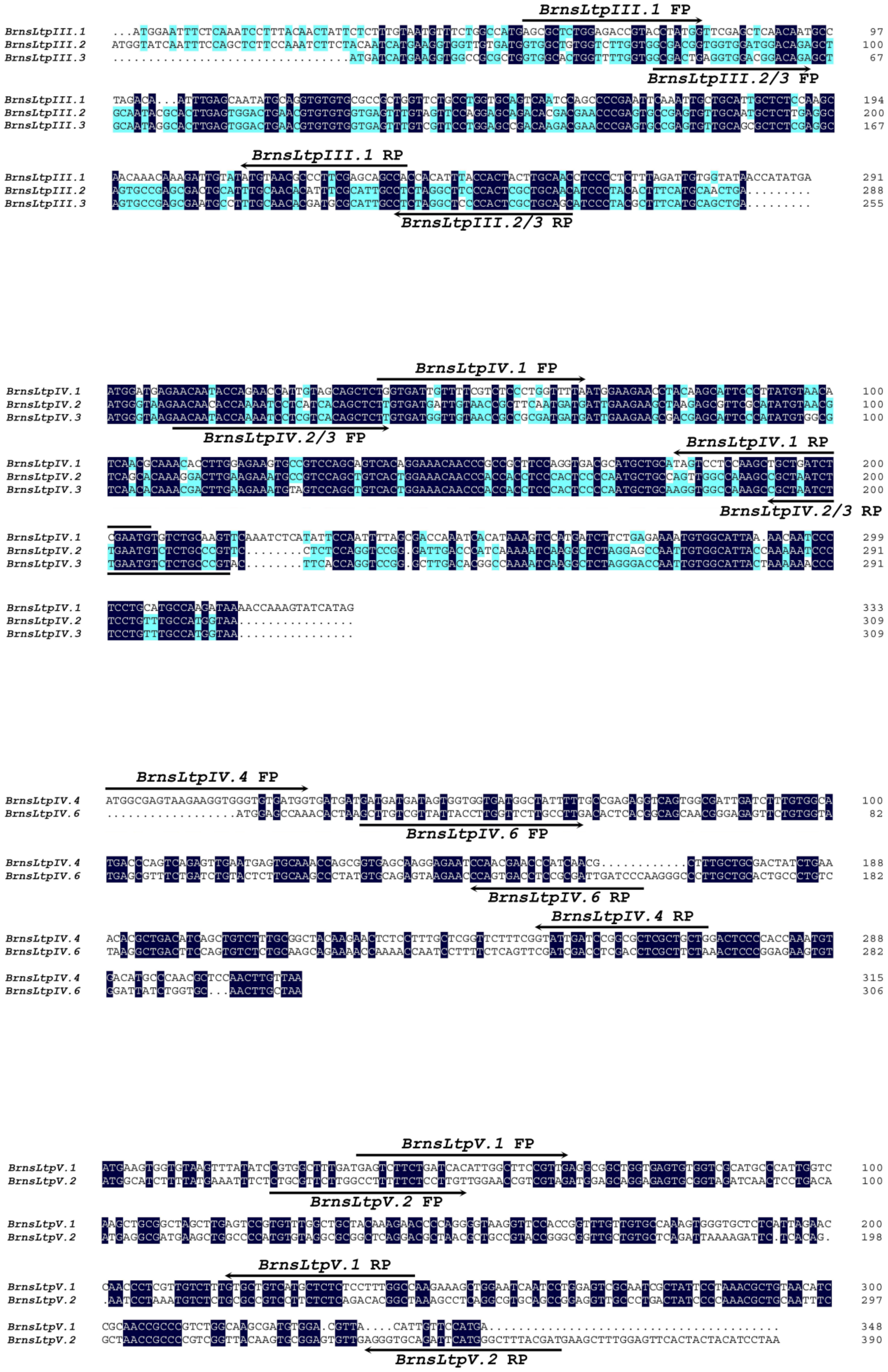
BrnsLtpIII.1 FP
BrnsLtpIII.2/3 FP
BrnsLtpIII.1 RP
BrnsLtpIII.2/3 RP
BrnsLtpIII.1
BrnsLtpIII.2
BrnsLtpIII.3
97 100 67
194 200 167
291 288 255
BrnsLtpIV.1 FP
BrnsLtpIV.2/3 FP
BrnsLtpIV.1 RP
BrnsLtpIV.2/3 RP
BrnsLtpIV.1
BrnsLtpIV.2
BrnsLtpIV.3
100 100 100
200 200 200
299 291 291
333 309 309
BrnsLtpIV.4 FP
BrnsLtpIV.6 FP
BrnsLtpIV.6 RP
BrnsLtpIV.4 RP
BrnsLtpIV.4
BrnsLtpIV.6
100 82
188 182
288 282
315 306
BrnsLtpV.1 FP
BrnsLtpV.2 FP
BrnsLtpV.1 RP
BrnsLtpV.2 RP
BrnsLtpV.1
BrnsLtpV.2
100 100
200 198
300 297
348 390

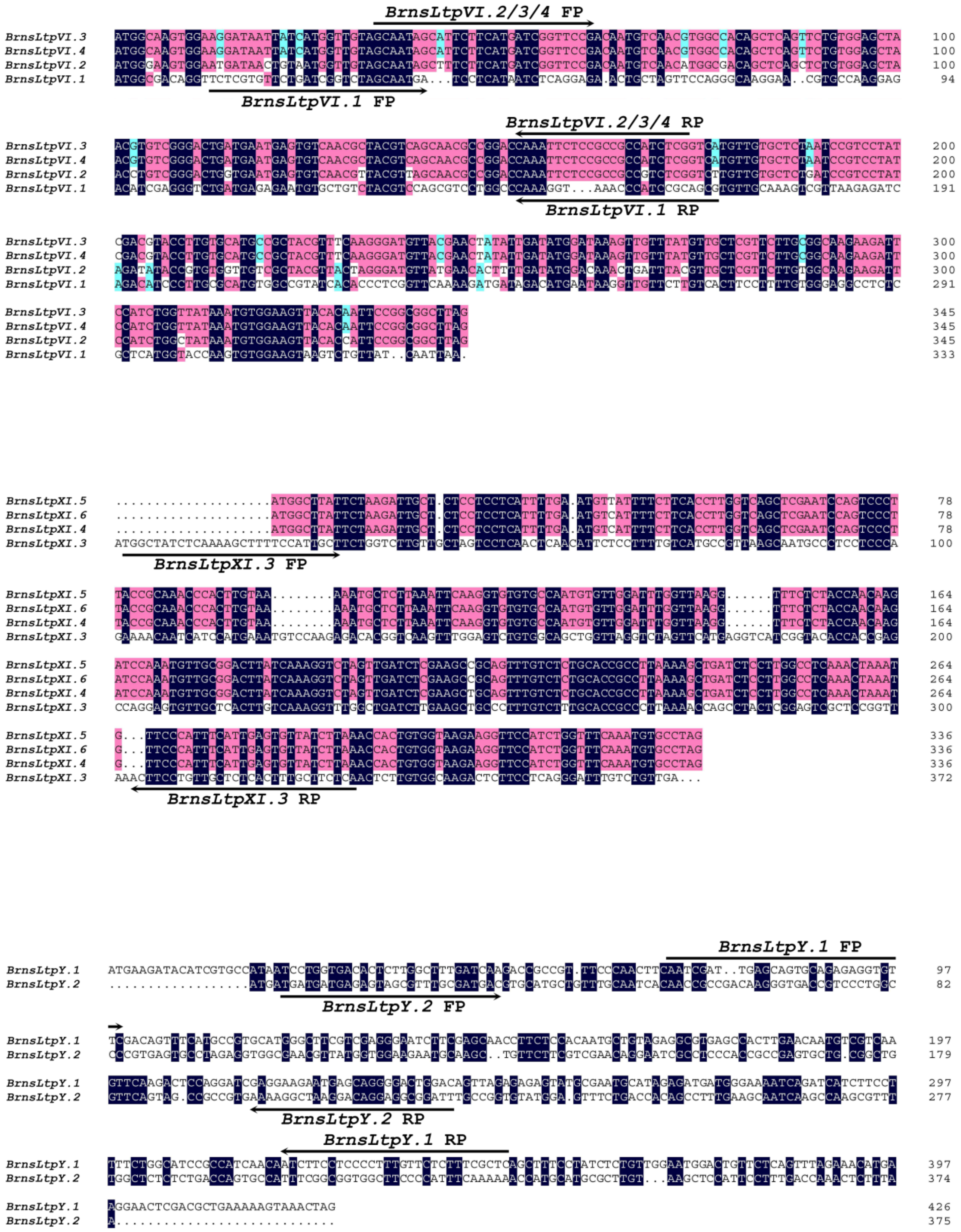
BrnsLtpVI.2/3/4 FP
BrnsLtpVI.1 FP
BrnsLtpVI.2/3/4 RP
BrnsLtpVI.1 RP
BrnsLtpVI.3
BrnsLtpVI.4
BrnsLtpVI.2
BrnsLtpVI.1
BrnsLtpXI.5
BrnsLtpXI.6
BrnsLtpXI.4
BrnsLtpXI.3
BrnsLtpXI.3 FP
BrnsLtpXI.3 RP
BrnsLtpY.1 FP
BrnsLtpY.2 FP
BrnsLtpY.2 RP
BrnsLtpY.1 RP
BrnsLtpY.1
BrnsLtpY.2
