# Supplementary figures and images for: Genome-Wide Survey and Expression Analysis of the Putative Non-Specific Lipid Transfer Proteins in Brassica rapa L
Source: PLoS One. 2014 Jan 31;9(1):e84556. doi: 10.1371/journal.pone.0084556 (PMC3908880; doi:10.1371/journal.pone.0084556)

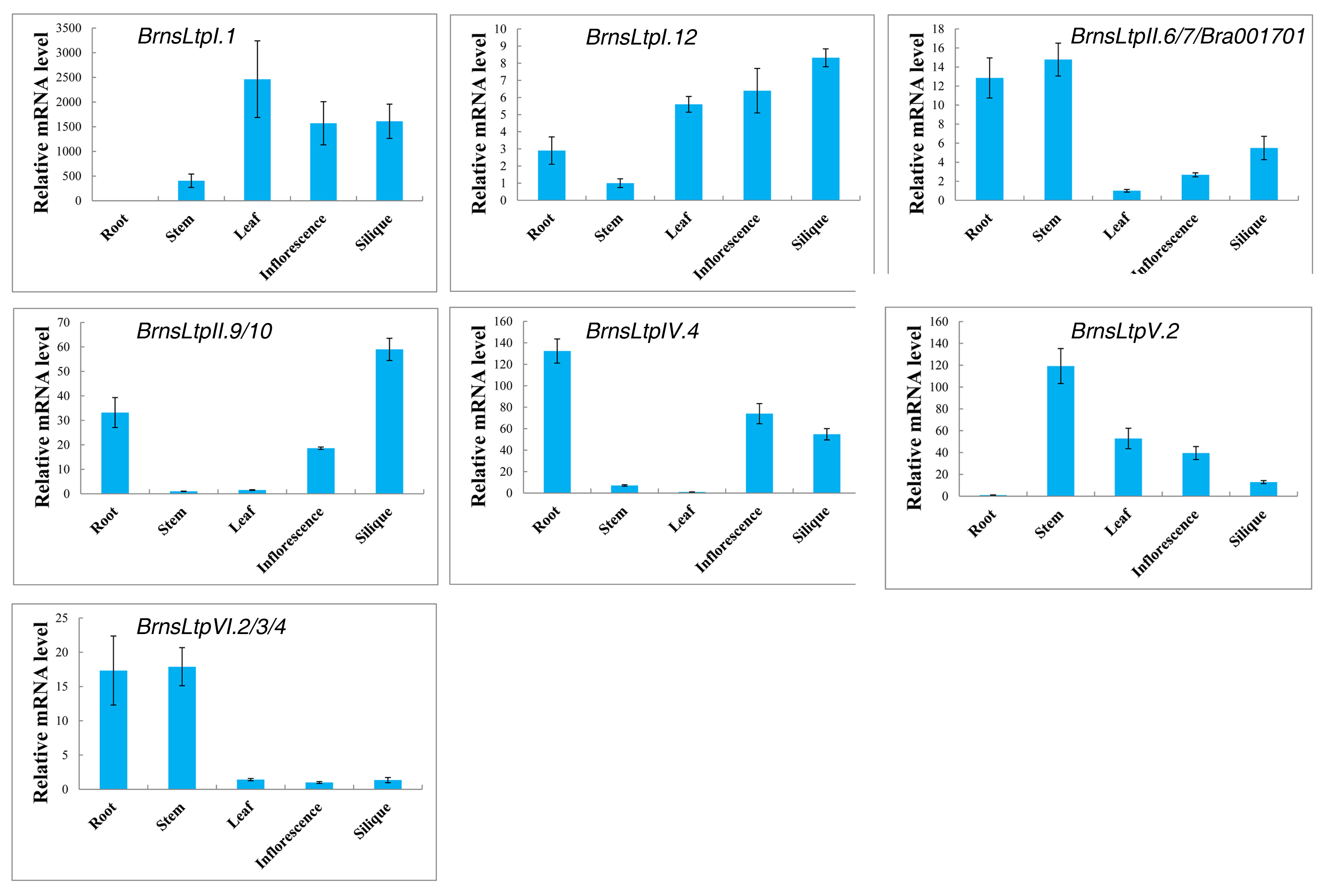

Supplement: Figure S4 — Quantitative RT-PCR analysis for selected BrnsLtp genes in tissues and organs of B. rapa with unspecific expression patterns. (TIF) [file pone.0084556.s004.tif]
