# Supplementary material for: Genome-Wide Survey and Expression Analysis of the Putative Non-Specific Lipid Transfer Proteins in Brassica rapa L
Source: PLoS One. 2014 Jan 31;9(1):e84556. doi: 10.1371/journal.pone.0084556 (PMC3908880; doi:10.1371/journal.pone.0084556)
Supplement: Table S1 — The coding sequences of BrnsLtp genes in B. rapa . (DOCX) [file pone.0084556.s005.docx]

**Table S1.** The coding sequences of *BrnsLtp* genes in *B. rapa*

| **Name** | **Coding sequence** |
| --- | --- |
| *BrnsLtpI.1* | ATGGCTGGTCTGATGAAGTTGGCATGCTTCCTCGTAGCATGCATGATTGTGGCGGGTCCAATCACAGCGAACGCGGCTCTGACCTGTGCCAGCGTGGTCAGCAACATGGCACGGTGCATAAGCTACTTGGGAGGAAGTGAGACCATAAGCGGTGCGTGCTGCAGCGGCATTAGGAGTATAAACGGTCTGTCCCGTACACCCTCGGACCGTCAGATAGCTTGCGGTTGCCTTAAAAGAGTCGCTACCCTCCCTAATATAAACGCTGACCGTGCGGCTGGATTACCCAATGCATGTGGAGTCAGTCTTCCTTACAATATCAGCAAAAGCGCCAACTGTACCCTGTATGTCGACTTTAACCTCTCTCGTTATCTCTCTTTATCTCTCTGA |
| *BrnsLtpI.2* | ATGAGATCTCTCTTCTTACTAGCCTTGTTCCTAGTTCTTGCTTTTCACCATGGTGAAGCAGCCGTGACTTGCAACAACGTGGTTGGTGATCTTTACCCTTGCCTCTCCTACGTGATGCAAGGCGGAAACTCCCCATCAACTAACTGCTGCAGCGGTGTCAGAACGCTCAACAGTCAGGCTCAAACCACTGCGGATCGTCAGAGCGTCTGCCGTTGCATCAAAAATGCTATTGGAGGAGCCTCTTACTCTTCAAGCAACCTTAAAAATGCTCTGTCTTTGCCTGCTAAGTGTGGTGTTAATCTCCCTTTCAGTATCAGCCCTTCCACCAACTGCAACAGGTAA |
| *BrnsLtpI.3* | ATGGCCGGTCTAGTGAAATTGGCATGCTTGGTCTTGGCCTGCATGATTGTGGCAGGTCCCATCACATCAAAGGCGGCTTTGAGCTGTGGCACCGTTAACACCAACGTGGCGGCATGCATTGGCTACTTGACCCAAGGTGGACCCCTTCCCAGAGCGTGCTGCACCGGTGTTAGTAAGCTTAACAGTATTGCCCGTACAACTCCCGACCGTAAGCAAGCTTGTCGTTGCCTTAAAACTGCTGCGAGCGCCTTAGGCTCTGGTCTCAACGCTGGCCGGGCAGCTGGACTTCCTAAGGCATGTGGAGTCAATGTTCCTTTCCCCATTAGCACTAGCATCAACTGCAACGGGCACCGTGAAATAAGCAGCGTTCGGATGAAGCTTGAGTGGATAATTCCCATGCTATGA |
| *BrnsLtpI.4* | ATGGCTGGTCTAATGAAGTTGGCATGCTTGGTCTTGGCCTGCATGATCGTGGCCGGTCCAATCACATCGAACGCGGCTCTGAGCTGTGGCACCGTTAGCGGCTACGTGGCACCGTGCATTGGCTACCTGGCCCAGGGTGCGCCGGCCCTTCCCAGAGCGTGCTGCAGCGGCGTTACTAGTCTAAACAACCTGGCCCGTACAACCCCAGACCGTCAGCAAGCTTGCCGTTGCCTTGTAGGAGCCGCTAACGCCTTCCCTACTCTCAACGCTGCCCGTGCAGCTGGACTTCCTAAGGCATGTGGAGTCAACATTCCTTACAAGATCAGCAAAACCACCAACTGCAACAGTGTGAAATGA |
| *BrnsLtpI.5* | ATGGCTTTGGCCCTGAGGTTCTTCACATGCCTTGTATTGACGGTGTGCATAGTTGCATCAGTAGATGCAGCAGTCTCATGTGGCACAGTGACAAGTAGCTTGGCTCCATGTGCCAACTACCTATCGAAAGGCGGGGATGTTCCTCCTCCGTGTTGTGCTGGAGTCACAAAGTTGAACGGTATGGCTCAAACCACACCAGACCGTCAACAAGCATGCAAATGCCTACAGTCCGCTGCAAAGACCGTTTCTGGTCTCGACCCAAGTCTAGCCGCTGGTCTTCCTGGAAAGTGCGGTGTTAGCATTCCCTATCCCATCTCCATGAGCACGAACTGCGACAACGTCAAGTGA |
| *BrnsLtpI.6* | ATGGCTTCGACACTGAGGTTCTTAACATGCCTTGTGACGGTGTGCATAGTTGCATCAGTAGGTGCACCAATCTCATGTGGCACAGTGGTACAAAGCATGAGTCCTTGTATCACATACCTATCGGGACGTATGGATTTGACGGCTGCATGTTGTGGGGGAGTCAGAGATTTGAATGCTATTGCTCAAACCACACCGGACCGTCAACAAACATGCAAATGCCTACAGGCCGTTGCTAAGAAGATTCCTGGTTTCAACCAAACTAGAGCCTCTGACCTTCCTGGAAAGTGTCGTGTTAGCTTTCCCTTTCCCATCTCCATAAGTACTAACTGCGACAACGTCCATCATGAGGATGAAGAGTATATGGTCTACGTACAATAA |
| *BrnsLtpI.7* | ATGATGTTCCCTTCAAAGATCACCACATGCCTCCTCGTCCTAGCCGTCTACATGGCATCCCCATCAGAGTCAACCATAACGTGTGGGACAGTGACAAGCACACTGGCACGGTGTATAGGCTACTTGACCAACAGTGGTTCATTGCCATCAGACTGCTGCGTTGGAGTCAAATCATTGAACCAAATGGCTCAGACCACACCGGACCGCCGACAAGTCTGTGAGTGCCTAAAATCTGCGGCTAAGGATATCACTGGCCTCAACACCGACCTTGTGGCCACACTCCCTACAACTTGTGGTGTTTCAGTTCCCTACCCCATTCGTTTTAGCACCAATTGTGACACTATATCGACTGCCGTGTGA |
| *BrnsLtpI.8* | ATGGCCGGTGTAATGAAGTTGGTATGCTTGGTCTTGGCCTGCATGATTGTGGCAGGTCCGATCACAGCGAACGCAGCTCTGACCTGTGGCACCGTTAACAGCAACGTGGCACCGTGCATTGGCTACATAACCCAAGGTGGACCCCTTCCCGGAGCGTGCTGCACCGGTGTTAGTAAACTTAACAGCATGGCCCGTACAACCCCGGACCGTCAGCAAGCTTGTCGTTGCCTTAAAACTGCTGCTAGCGCCTTAGGCCCTAGTCTCAACGCTGGCCGTGCAGCTGGAATTCCCAAGGCATGTGGAGTCAGTGTTCCTTTCCCAATCAGCACCAACACCAACTGCAACAGCGTGAAATGA |
| *BrnsLtpI.9* | ATGGCTGGTCTAATGAAATTGGCATGCTTGGTCTTGGCCTGCATGATTGTGGCCGGTCCAATCACAACGAACGCGGCTCTAAGCTGTGGAACCGTTAGCGGTAACTTGGCAGCATGCATTGGCTACTTAACCCAAAATGGGCCCCTTCCCAGAGGGTGCTGCACCGGCGTTACTAATCTAAACAACATGGCCCGTACAACCCCGGACCGTCAGCAAGCTTGCCGTTGTCTTGTAGGAGCCGCTAACTCCTTCCCTACTCTCAACGCTGCCCGAGCTGCTGGACTTCCTAAGGCATGTGGAGTCAATATTCCTTACAAAATCAGCAAAAGCACCAACTGCAACAGCGTTAGATGA |
| *BrnsLtpI.10* | ATGGCTATTGCTCTTAGGTTCTTTACATGCCTTGTTTTGACGGTGTGCATTGTCGCATCAGTAGATGCAGCAATCACATGTGGCACAGTGACAAGTAGCTTGGCTCCATGTGCCACCTACCTATCGAGTGGCGGGGAGGTGCCACCTCCATGCTGTGCGGGAGTCAAAAAATTGAATGGTATGGCTCAAACCACAGCGGACCGCCAACAAGCATGCAAGTGTCTAAAGGCCGCTGCACAGGGCATCAACCCAAGTCTAGCCTCTAGCCTTCCTGGAAAATGCAGTGTTAGCATTCCCTATCCCATCTCCATGAGTACCAACTGCGACAACGTCAAGTGA |
| *BrnsLtpI.11* | ATGACTATGGGTCTGAAGTTCTTTACATGCCTTGTTTTGACGGTCTGCATTGCCGCATCAGTAGATGCAGCACTCACATGTGGTACAGTGACAAGTAGCTTGGCTCCATGTGCCACCTACCTATCGAAAGGTGGGGCGGTGGTGCCGGGTCCATGTTGTGCGGGAGTAAAAAAATTGAATGATATGGCTCAAACCACACCGGACCGCCAACAAGCATGCAAATGCCTAAAGGCCGCTGCAAAAAGCATCAATCCAAGTCTAGCCTCCGGCCTTCCTGGAAAGTGCAGTGTTAGCATTCCCTATCCTATCTCCATGAGCACCAACTGCGACAAGTAA |
| *BrnsLtpI.12* | ATGGCTTATTCTAAGATTGCTCTCCTCCTCATTTTGAATGTTATTTTCTTCACCTTGGTCAGCTCGAATCCAGTCCCTTACCGCAAACCCACTTGTAAAAATGCTCTTAAATTCAAGGTGTGTGCCAATGTGTTGGATTTGGTTAAGGTTTCTCTACCAACAAGATCCAAATGTTGCGGACTTATCAAAGGTCTAGTTGATCTCGAAGCCGCAGTTTGTCTTGAAGCCGCAGTTTGTCTCTGCACTGCCTTAAAAGCTGATCTCCTTGGCCTCAAACTAAATGTTCCCATTTCATTGAGTGTTATCTTAAACCACTGTGGTAAGAAGGTTCCATCTGGTTTCAAATGTGCCTAG |
| *BrnsLtpI.13* | ATGGCTTCGGCTCTGAGTTTTTTCACATGCCTTGTTTTGACTGTGTGCATAGTTGCATCAGTAGATGCAGCAATCTCATGTGGCACAGTGACAAGTAACTTGGTTCCATGTGCCGGCTATCTAATGAAAGGCGGGCCGGTGCCAGCTTCATGCTGCGCCGGAGTTTCAAAATTGAACAGTATGGCTAAAACCACACCGGACCGCCAACAAGCATGTAAATGCCTAAAGACCGCTGCAAAGAGCGTCAATCCAAGTCTAGCCTCTAGCCTTCCTGGAAAGTGCGGTGTTAGCATTCCCTATCCTATCTCCATGAGCACTAACTGCAACACGTAA |
| *BrnsLtpI.14* | ATGAGATCTCTCTTACTAGCCTTGTTCCTAGTTCTTGCTTTCCACCGTGGTGAAGCAGCCGTGTCTTGCAACGCAGTGGTTGGAGATCTTTACCCTTGTCTCTCCTACGTGGTTCAAGGCGGTAACGTCCCAGCGAACTGCTGCAACGGCATCAGAACGCTCAACAGTCAGGCCCAAACCCCTGTGGACCGTCAGGGCGTTTGCCGTTGCATCAAAAATGCTATCGGAGGAGTCTCTTTCTCTTCTAACAACGTCAACAATGCTCAGTCTCTGCCTGCTAAGTGTGGTGTGAATCTCCCTTACAGTATCAGCCCTTCCACCAACTGCGACAGGTAA |
| *BrnsLtpI.15* | ATGGAGGGGCTCTTAAAGTTGTCGACGTTGGTGATTGTGTGCATGTTAGTGTCCGCTCCAATGGCGTCCGAGGCAGCAATCTCTTGCGGCGCAGTCGCCAGCAACTTAGGCCAATGCATTAACTACCTGACCCGAGGTGGTTTCGTTCCTCGAGGGTGTTGTTCTGGCGTTAGGAGGCTCAACAGCATGGCTCGTACCACCCGTGACCGCCAACAAGCTTGTCGCTGCATCCAAGGAGCAGCTAGAGCCTTGGGTTCCAGACTTAACCCAGGCCGTGCTGCACGTCTTCCTGGTGCTTGTCGTGTTAGGATCGCTTACCCCATCAGCGCAAGAACCAACTGTAACAAGTAA |
| *BrnsLtpI.16* | ATGGCGTTTGCTTCAAAGATCATCACATGCCTCCTCGTCCTTAAGGTCTACATGGCAGCTCCAGCAGAGTCACACATAACGTGTGGGATAGTGACATCCACATTGGCTCAATGCATGGGCTACTTGACCAACTTTTTTCCAGTGCCATCAGACTACTGCTGCGCGGAAGTCAAAGGACTGAATCAAATGGCTCAGACCACTCCGGACCGCCGACAAGTATGTAAGTGCCTTAAAGCAGTGGCTAAGGAAAACAAAGGATTCATTAGCATCGAGCTTGTGGGCACACTCCCTACTATTTGTGGTGTTTCAGTTCCCTACCCATTTAATTTCAGCACCAATTGTGACACTATTTCGACTGCCGTGTGA |
| *BrnsLtpI.17* | ATGAGCATATTGAAAAGTTTAGTGACCATCTTCGTTCTCGGAATATTTTTAACACCTCGTTATTCCGAATCTGCGATATCTTGCAGTGTTGTTCTAAGCGATTTGCAGCCATGTGTTAGCTACTTGACTAGCGGAAGTGGACAACCTCCGGAGACTTGCTGCGACGGAGTTAGGAGTTTAGATGCAGCGACCACTACATCCGCTGATAAAAAGGCGGCTTGTCAATGCATCAAGTCAGTGGCCAACAGTGTCACCGTGAAGCCTGAATTGGCTAAAGCCCTCGCTAGCAATTGTAACGCGAGCTTGCCTGTTGATGCATCTTCTACGGTCGACTGCAATACGGTTGGTTGA |
| *BrnsLtpI.18* | ATGACGTTTGCTTCAAAGATCATCACATGCCTCCTCGTCCTTACGATCTACATTGCAGCTCCAACAGAGTCACACATAACGTGTGGGACAGTGACAAGCACAATGACACAATGCATCAGCTACTTGACCAACGGTGGTCCATTGCCGTCAAGCTGCTGTGTGGCAGTCAAATCATTGAACCAAATGGCTCAGACCACACCAGATCGCCGACAAGTATGTGAGTGCCTTAAATCAGCCGGTAAGGAAATTAAAGGCCTCAATATCGACCTTGTGGCCGCACTCCCTACCACTTGTGGTGTTTCACTTTCCTACCCCATTGGTTTCAACACCAATTGTGACAGTATATCGATTGCCGTGTGA |
| *BrnsLtpI.19* | ATGGAGGGCTTCATAAAGCTGTCAACATTGTTGATTGTGTGCATGTTAGTGTCAGCTCCAATGGCAGAGGCAGCAATCTCGTGTGGCGCCGTCGCCAGCAACTTGGGGCAATGCATTAACTACCTGACCAGGGGCGGATTCGTTCCTCGAGGATGTTGTTCGGGTGTTCAGAGGCTCCACAGCATGGCTCGGACCACACGTGATCGGCAACAAGCTTGTCGCTGCATTCAAGGAGCAGCTAGAGCCTTGGGTTCCAGACTTAACCCAGGCCGTGCTGCTCGTCTTCCTGGTGCTTGCCGTGTTAGGATCTCTTACCCCATCAGCGCAAGAACCAACTGTAACAAGTAA |
| *BrnsLtpII.1* | ATGAAGTTTTCTTGCTCAAAACCAGTGTTGTTCACTTGTGCAATCCTTCTCTTGCTCATTGTTGCTCAAGAAAACAGAGTTGTTGCAGGACAGTCATGTGACCCTATGCAGCTTATCCCATGCGAGGAGGCCATACTGAAAGGCTCTAAGCCGTCGGATACATGTTGCACCAGGCTGAACCAGCAACAACACTGTGTCTGCCAGTACATGAAGAACCCCAATTTCAAATCTTTCCTCGACTCACCAAATGCTAAAAAAATCGCCACTGATTGTCATTGTCCTAAACCCAAGTGCTAG |
| *BrnsLtpII.2* | ATGAAGTTCACAGGAGTAGTATGCATCGCGTTTGTGATAGTCCTTGTATCGGCTTCAGCTCCGACCAAAGAAGTCTTGGAAGAGAAAGTGGCATGCAACTCGACAGAACATATAACATGCATACCAGCATTACAATCCGGAAGTCAACCGTCCGCTGAATGCTGCGGAAAACTGAAAGAAGAAGAGTCGTGTTTGTGTGGTTACATACAAAATCCATTGTTTTCTCAGTATGTTACATCTGCAAATGTTCACATGGTTTTAGTAACTTGTGGTATCCCTTATCCTTCTTGCTAG |
| *BrnsLtpII.3* | ATGGTAAAGGTGATGTGGGGTTCCAGTTTAGCTCTCGCCGCAGCGCTTCTCCTGGTGACGGTGGCAAACATTCCGGTGGCGGAGGGAGTGACGTGCTCGCCGACGGAACTGACTTCATGTTCGTCGGCGTTTATGTCAGCTTCGCCGCCGTCAGCAACGTGCTGCGCCAAGCTGAGAGAGCAGAAGCCATGCCTTTGTGGATATCTGAGGAACCCTGCCCTCAGCCAGTACGTTAACTCTCCAAACGCCAAGAAAGTTGCCAGCAGTTGCAATGTTGCAACCCCGAAATGTTAA |
| *BrnsLtpII.4* | ATGAAGGCCTCTTGCACAAAACCAGTCTTGATCACTTGTACAATCCTTCTCTTGCTCATTGTTGCTCAAGAAAACAGAGTCGCTGCAGCAGAGCAATGTAATCCCATGCAGCTCATGCCATGCGAGGACGCCATAATGAAAGGATCTACGCCCTCCAATGAGTGTTGCACCAGGTTGAAGCAGCAACAACACTGTATCTGCCAGTACATGAAGAACCCAAACTTCAAATCTTTCCTTAACTCACCAAATGCGAAAATGGTCGCCAGTCATTGTCAGTGTAAACCCAAGTGCTAG |
| *BrnsLtpII.5* | ATGAACTTCACAGGAGCAATATGCATCGCATTTGTGATAGTCCTTGTTTCGTCTTTGGCTCTCACCAATGCAGCCGTGGAAGATGAGAAAGTACTGGCATGCAATCCGAAAGAACTTAACCCATGCAGCCCAGCTGTAAAAACCGGAAGTAAGCCGTCAACAGAGTGTTGCGCAATGCTGAAAAAAGAAGAGCCGTGTCTATGTGGTTACATAAATGATCCAGTTTATGGTCAGTATATTAAATCCAAAAATGCTCATAAAGCCTTCTCGTCTTGTGGTATACCTCCTCTTTCTTGTTGA |
| *BrnsLtpII.6* | ATGGAAATGATCAAGGCCAAATGGGTTTCCATTGTCGCCCTCGCGGCTATTTTCCTAGTAGTGATCCTAGTACCGGCGGCAGAAGCGGTGACGTGCTCGCCAATGCAGCTAAGCCCATGTGCATCGGCAATAACGTCGTCTTCTCAAACTTCAGCGTTGTGCTGCGCGAAGCTGAAGGAGCAGAAGCCATGCCTCTGTGGGTACATGAGAAACCGTAGCCTCCGTCGCTTTGTCAGCTCTCCCAACGCTCGTAAAGTCTCTAACCGCTGCAAGCTTCCCATCCCAAGGTGTTGA |
| *BrnsLtpII.7* | ATGGTGATGATCAAGGCTACATGGGTTTCCATCTTCGCCATCGCGGCGGTTCTCCTAGTGATTCTAGCCCCGGCGGCAGAAGCAGTGACGTGCTCGCCAATGCAGCTAAGCCCATGTGCGCAGGCGATAACGTCGTCTTCTCCACCGTCAGCGTTGTGCTGCGCGAAGCTGAAGGAACAGAAGCCATGCCTATGTGGGTACATGAGAAACCCTAGCCTCCGTCGCTTCGTCAGCTCTCCCAACGCTCGTAAAGTCTCTAACCGCTGCAAGCTCCCCATCCCAAGGTGTTGA |
| *BrnsLtpII.8* | ATGAAGTTAACAGCAATAGGACTCGTTGCGATGGTGACAATAGTGGTGCAATTGTCACCGACAATGGCGTGCGATGTAAAAGATTTGTCCCCGTGCTTGCTACCGATAGCCGTTTTTCCAGAGTCTCCGACTGCAGCGTGTTGCCAAACATTGAGAGATCAGGGGCCTTGTCTGTGTGTTTTCATCAATAATTCATGGATTTGGATTGGGCCTACTCTGACATCTCCAAATGGTCACAAACTTTTCGCAGCTTGTCAAGTTCCCTTTCCTAGTTGTGGAAACTGA |
| *BrnsLtpII.9* | ATGAAGTTCACAGGAGCAATATGCATCGCACTTGTGATAGTCCTAGTTTCGTCTTTGGATCTGACCAGTGCAGCCGTGGAAGAAGAGATAAAAGTGGCATGTGTCGTAACAGAACTTATCCCATGTCTCGAATCATCAATAATCGGAGTTCACCCTTATCCAGAATGTTGTGTAACACTGAAAGCACAACAGTCGTGTCTATGTGGTTACATACAAAATCCAGTATATGGTGGGTTTTTTAAGAATGCTCACAGTGTCTTCACGGGTTGTGGTGTACCTTACCCTACTTGTTGA |
| *BrnsLtpII.10* | ATGAAGTTCACAGGAGCAATATGCATCGCACTTGTGATAGTCCTGGTGTCATCTTTGGATCTGACCAGTGCAGCCGTGGAAGAGGAGATAAAAGTGGCGTGTGTCCAAACAGAACTTATCCCATGTTTCGTAGCAGCATTTATCGGAAGTCAGCCGTCAGCAGAATGTTGCGAAAAACTGAAAGAACAACAGTCGTGTCTATGTGGTTACATCTCAAATCCAGTATTTGGTCAGTTTTATAAAAATGCTCAAAATGTCTTCAAGGCTTGTGGTGTACCTTACCCTACTTGTTAA |
| *BrnsLtpII.11* | ATGAAGTTCACTACTCTAATGGTCATCACATTGGTGATAATCGCCATGTCGTCTCCTGTTCCAATTAGAGCAACCTCGGTTGAAAGTTTCGGAGAAGTGGCACAATCGTGTGTTGTGACAGAACTCGCCCCATGCTTACCAGCAATGACCACGGCAGGAGACCCGACTACAGAATGCTGCGACAAACTGGTAGAGCAGAAACCATGTCTTTGTGGTTATATTCGAAACCCAGCCTATAGTATGTATGTTACTTCTCCAAACGGTCGCAAAGTCTTAGATTTTTGTAAGGTTCCTTTTCCTAGTTGTTAA |
| *BrnsLtpII.12* | ATGAGGTTCACAGGAGTAGTATGCATCGCGTTTGTGATAGTCCTTGTGTCGGCTTTAGCTCCGACCAAAGCAGACTTGGAAGAGAAAGTGGCATGCATCCCGACAGAACTTATGACATGCATACCAGCATTACAAACCGGAAGTCAACCGTCTGCTGAATGCTGCGGAAAACTGAAAGAACAAGAGTCGTGTTTGTGTGGTTACATACAAAATCCATTGTTTTCTCAGTATGTTACATCTGAAAATGCTCACAAAATTTTAGCGACTTGTGGCATCCCTTATCCTACTTGTTAA |
| *BrnsLtpII.13* | ATGGTGAAGGTGATGTGGGTTTTCGTTTTAGCTCTCGTGGCAGCGCTTCTCCTGGTGACGGTGGAAAAAATTCCGGTGGCCGAAGGAGTGACGTGCTCGGTGACGGAATTGTCTCCATGTTTGGCGGCGTTTATGTCATCTTCGCAACCGTCGGCGTCGTGCTGTGCCAAGCTGAGAGAGCAGAAGCCATGCCTTTGTGGGTACATGAGGAACCCTGGCCTCCGCCAATATGTTACCTCCCCAAACGCTAAAAAAGTCTCCAACAGTTGCAAGGTTGCTTCCCCAAACTGTTAA |
| *BrnsLtpII.14* | ATGAAGTTTACAACACTAGCATCCATCGCGTTTGTTGTTGTTGTCCTCTTTTCATCAACGGCGGCTCCGATCAATTCCCAACTTATTCAATCTAATTCACCTTGCACCACTATTGATATCACAGGATGCGTTCCAGCCATACTCTATGGAGCACCACTATCGCCAGAATGTTGTCGGAATCTGAATGTACAACAACCATGTTATTGTGATTTCATAAAGAATGCAGGTTTAAAGCCATATATTACTTCCCCTCAAGGTCATGCGGCTCTAGCGTCTTGTGGTATTCCTTATCCTACTTGTTGA |
| *BrnsLtpII.15* | ATGGTCTTGACACTCATGGTTTTCGTCATACTTCTGACATTGTTTCCGGCTCCAAACGAAGCGGCAGACACAAATGTGGAAGCAGCATGTGACCCAAAGCAGCTTCAGCCTTGCCTGGCCGCGATTACTGGAGGAGGACAGCCCTCGGGTGACTGTTGTGCAAAGCTGAAGGAGCAACAACCATGCTTGTGTGGGTTTTCTAAGAACCCTGCTTTTGCTCAGTACATTAGCTCTCCCAACTCTCGCAAAGTCCTAACCGCTTGTGGTATTCCTTACCCAAGTTGTTAA |
| *BrnsLtpIII.1* | ATGGAATTTCTCAAATCCTTTACAACTATTCTCTTTGTAATGTTTCTGGCCATGAGCGCTCTGGAGACCGTACCTATGGTTCGAGCTCAACAATGCCTAGACAATTTGAGCAATATGCAGGTGTGTGCGCCGCTGGTTCTGCCTGGTGCAGTCAATCCAGCCCCGAATTCAAATTGCTGCATTGCTCTCCAAGCAACAAACAAAGATTGTATATGTAACGCCCTTCGAGCAGCCACCACATTTACCACTACTTGCAACCTCCCCTCTTTAGATTGTGGTATAACCATATGA |
| *BrnsLtpIII.2* | ATGGTATCAATTTCCAGCTCTTCCAAATCTTCTACAATCATGAAGGTGGTTGTGATGGTGGCTGTGGTCTTGGTGGCGACGGTGGTGGATGGACAGAGCTGCAATACGCACTTGAGTGGACTGAACGTGTGTGGTGAGTTTGTAGTTCCAGGAGCAGACACGACGAACCCGAGTGCCGAGTGTTGCAATGCTCTTGAGGCAGTGCCGAGCGACTGCATTTGCAACACATTTCGCATTGCCTCTAGGCTTCCCACTCGCTGCAACATCCCTACACTTTCATGCAACTGA |
| *BrnsLtpIII.3* | ATGATCATGAAGGTGGCCGCGCTGGTGGCACTGGTTTTGGTGGCGACTGAGGTGGACGGACAGAGCTGCAATAGGCACTTGAGTGGACTGAACGTGTGTGGTGAGTTTGTCGTTCCTGGAGCCGACAAGACGAACCCGAGTGCCGAGTGTTGCAGCGCTCTCGAGGCAGTGCCGAGCGAATGCCTTTGCAACACGATGCGCATTGCCTCTAGGCTCCCCACTCGCTGCAGCATCCCTACGCTTTCATGCAGCTGA |
| *BrnsLtpIV.1* | ATGGATGAGAACAATACCAGAACCATTGTAGCAGCTCTGGTGATTGTTTTCGTCTCCCTGGTTTTAATGGAAGAACCTACAAGCATTCCCTTATGTAACATCAACGCAAACACCTTGGAGAAGTGCCGTCCAGCAGTCACAGGAAACAACCCGCCGCTTCCAGGTGACGCATGCTGCATAGTCCTCCAAGCTGCTGATCTCGAATGTGTCTGCAAGTTCAAATCTCATATTCCAATTTTAGCGACCAAATCACATAAAGTCCATGATCTTCTGAGAAAATGTGGCATTAAAACAATCCCTCCTGCATGCCAAGATAAAACCAAAGTATCATAG |
| *BrnsLtpIV.2* | ATGGGTAAGAACAACACCAAAATCCTCATCACAGCTCTTGTGATGATTGTAACCGCTTCAATGATGATTGAAGAAGCTAAGAGCGTTCGCATATGTAACGTCAGCACAAAGGACTTGAAGAAATGCCGTCCAGCTGTCACTGGAAACAACCCACCACCTCCCACTCCCCAATGCTGCCAGTTGGCCAAAGCCGCTAATCTTGAATGTCTCTGCCCGTTCCTCTCCAGGTCCGGGATTGACCCATCAAAAATCAAGGCTCTAGGAGCCAATTGTGGCATTACCAAAAATCCCTCCTGTTTGCCATGGTAA |
| *BrnsLtpIV.3* | ATGGGTAAGAACAATACCAAAATCCTCGTCACAGCTCTTGTGATGGTTGTAACCGCCGCGATGATGATTGAAGAAGCGACGAGCATTCCCATATGTGGCGTCAACACAAACGACTTGAAGAAATGTAGTCCAGCTGTCACTGGAAACAACCCACCACCTCCCACTCCCCAATGCTGCAAGGTGGCCAAAGCCGCTAATCTTGAATGTCTCTGCCCGTACTTCACCAGGTCCGGGCTTGACACGGCCAAAATCAAGGCTCTAGGGACCAATTGTGGCATTACTAAAAAACCCTCCTGTTTGCCATGGTAA |
| *BrnsLtpIV.4* | ATGGCGAGTAAGAAGGTGGGTGTGATGGTGATGATGATGATGATAGTGGTGGTGATGGCTATTTTTGCCGAGAGGTCAGTGGCGATTGATCTTTGTGGCATGACCCAGTCAGAGTTGAATGAGTGCAAACCAGCGGTGAGCAAGGAGAATCCAACGAACCCATCAACGCTTTGCTGCGACTATCTGAAACACGCTGACATCAGCTGTCTTTGCGGCTACAAGAACTCTCCTTTGCTCGGTTCTTTCGGTATTGATCCGGCGCTCGCTGCTGGACTCCCCACCAAATGTGACATGCCCAACGCTCCAACTTGTTAA |
| *BrnsLtpIV.5* | ATGGCTTACGTTAACAAGGTTTCAGCGGTTGCAGCCATTTTATTTTTTGCGGTTGCGGTTGCTCCGCTGCTAGCTGAGCCACAGACACCAATGTTTCCTAAGATGGATCCTGTATGTGCCTCACTGATGCCTAACCTTCTCGAAAAGTGTTTCTCAACCGTTAGAGAAACGCCAACGGATGATTGCTGCAGCGATCTCAAATCCGCGACCACAACCCAAGTCACTTGCCTTTGTGATAATTACATTGCAAACCCGGCGGTTGTGAACTTCACGGGACCTTACTCCGCCGGAATCACAACCAAATGTGGCGTTTTTGACAAATACTCTTGCAACGGTAGTAGTAATGGTACGTAA |
| *BrnsLtpIV.6* | ATGGAGCCAAACACTAAGCTTGTCGTTATTACCTTGGTTCTTGCCTTGACACTCACGGCAGCAACGGGAGAGTTCTGTGGTATGAGCGTTTCTGATCTGTACTCTTGCAAGCCCTATGTGCAGAGTAAGAACCCAGTGACCTCCGCGATTGATCCCAAGGGCCCTTGCTGCACTGCCCTGTCTAAGGCTGACTTCCAGTGTCTCTGCAAGCAGAAAACCAAAACCAATCCTTTTCTCAGTTCGATCGACCTCGACCTCGCTTCTAAACTCCCGGAGAAGTGTGGATTATCTGGTGCAACTTGCTAA |
| *BrnsLtpIV.7* | ATGGGTAAGAACAATACCACAATCCTCATCATTGCGATGGTTTTAACCACGGCGATGATAATGGAAGAAGCTAAGAGTTATCCTATATGCAACACCGACACAAACGACTTGCAGAAATGTTCTCCAGCTGTAACTGGAAACAACCCGCCAGCTCCGGGACCCGACTGCTGCGCAGTGGCTAAGTCCGCTGATCTTGAATGTCTCTGCCCGTACCTCTCCCTGTCCGGGATTGATCCTTCAAAAATCAAGTCTGTTCTGGCCAGTTGTGGCGTGGGCAATCCCTCCTGTTTGTCATGGTAA |
| *BrnsLtpIV.8* | ATGGGTAAGAACAACACCAATATCCTTACGCAATCTACAGTTCTTGCAATGGTTTTAACCGCTGCAATAATGGTGAAAGAAGTTTCAAGTCTTACCATTTGCAAAATCGACATAAACGATATGCAGAAATGTCGGCCAGCCGTCATTGGAATCAACCCCCCGCCTCCGGTCAACGAGTGCTGCGTAGTGGTCCGATCCGCTAATCTGGAATGCTTCTGCGGATTCAAGTTTTATCTCCCTATTTTAGGAATTGATCCATCTAAAGTTGCGGCTCTTGTAGCCAAATGTGACGTTACAACAATCCCTCCTAGTTGCCAAGTGTCTAAAGTTTTGGCTTCGCGCAGTTTGAAGAATGGAGCTTGA |
| *BrnsLtpV.1* | ATGAAGTGGTGTAAGTTTATATCCGTGGCTTTGATGAGTCTTCTGATCACATTGGCTTCCGTTGAGGCGGCTGGTGAGTGTGGTCGCATGCCCATTGGTCAAGCTGCGGCTAGCTTGAGTCCGTGTTTGGCTGCTACAAAGAACCCCAGGGGTAAGGTTCCACCGGTTTGTTGTGCCAAAGTGGGTGCTCTCATTAGAACCAACCCTCGTTGTCTTTGTGCTGTCATGCTCTCTCCTTTGGCCAAGAAAGCTGGAATCAATCCTGGAGTCGCAATCGCTATTCCTAAACGCTGTAACATCCGCAACCGCCCGTCTGGCAAGCGATGTGGACGTTACATTGTTCCATGA |
| *BrnsLtpV.2* | ATGGCATCTTTTATGAAATTTCTCTGCGTTCTTGGCCTTTTTCTCCTTGTTGGAACCGTCGTAGATGGAGCAGGAGAGTGCGGTAGATCAACTCCTGACAATGAGGCGATGAAGCTGGCCCCATGTGTAGGCGCGGCTCAGGACGCTAACGCTGCCGTACCGGGCGGTTGCTGTGCTCAGATTAAAAGATTCTCACAGAATCCTAAATGTCTCTGCGCCGTCCTTCTCTCAGACACGGCTAAAGCCTCAGGCGTGCAGCCGGAGGTTGCCCTGACTATCCCCAAACGCTGCAATTTCGCTAACCGCCCCGTCGGTTACAAGTGCGGAGTGTTGAGGGTGCAGATTCATGGGCTTTACGATGAAGCTTTGGAGTTCACTACTACATCCTAA |
| *BrnsLtpVI.1* | ATGGCGACAGGTTCTCGTGTTCTGATCGGTCTAGCAATGATCCTCATAATCTCAGGAGAACTGCTAGTTCCAGGGCAAGGAACGTGCCAAGGAGACATCGAGGGTCTGATGAGAGAATGTGCTGTCTACGTCCAGCGTCCTGGCCCAAAGGTAAACCCATCCGCAGCGTGTTGCAAAGTCGTTAAGAGATCAGACATCCCTTGCGCATGTGGCCGTATCACACCCTCGGTTCAAAAGATGATAGACATGAATAAGGTTGTTCTTGTCACTTCCTTTTGTGGGAGGCCTCTCGCTCATGGTACCAAGTGTGGAAGTAAGTCTGTTATCAATTAA |
| *BrnsLtpVI.2* | ATGGGAAGTGGAATGATAACTGTAATGGTTGTAGCAATAGCTTTCTTCATGATCGGTTCCGACAATGTCAACATGGCGACAGCTCAGCTCTGTGGAGCTAACCTGTCGGGACTGGTGAATGAGTGTCAACGTTACGTTAGCAACGCCGGACCAAATTCTCCGCCGCCGTCTCGGTCTTGTTGTGCTCTGATCCGTCCTATAGATATACCGTGTGGTTGTCGCTACGTTACTAGGGATGTTATGAACACTTTTGATATGGACAAACTGATTTACGTTGCTCGTTCTTGTGGCAAGAAGATTCCATCTGGCTATAAATGTGGAAGTTACACCATTCCGGCGGCTTAG |
| *BrnsLtpVI.3* | ATGGCAAGTGGAAGGATAATTATCATGGTTGTAGCAATAGCATTCTTCATGATCGGTTCCGACAATGTCAACGTGGCCACAGCTCAGTTCTGTGGAGCTAACGTGTCGGGACTGATGAATGAGTGTCAACGCTACGTCAGCAACGCCGGACCAAATTCTCCGCCGCCATCTCGGTCATGTTGTGCTCTAATCCGTCCTATCGACGTACCTTGTGCATGCCGCTACGTTTCAAGGGATGTTACGAACTATATTGATATGGATAAAGTTGTTTATGTTGCTCGTTCTTGCGGCAAGAAGATTCCATCTGGTTATAAATGTGGAAGTTACACAATTCCGGCGGCTTAG |
| *BrnsLtpVI.4* | ATGGCAAGTGGAAGGATAATTATCATGGTTGTAGCAATAGCATTCTTCATGATCGGTTCCGACAATGTCAACGTGGCCACAGCTCAGTTCTGTGGAGCTAACGTGTCGGGACTGATGAATGAGTGTCAACGCTACGTCAGCAACGCCGGACCAAATTCTCCGCCGCCATCTCGGTCATGTTGTGCTCTAATCCGTCCTATCGACGTACCTTGTGCATGCCGCTACGTTTCAAGGGATGTTACGAACTATATTGATATGGATAAAGTTGTTTATGTTGCTCGTTCTTGCGGCAAGAAGATTCCATCTGGTTATAAATGTGGAAGTTACACAATTCCGGCGGCTTAG |
| *BrnsLtpVIII.1* | ATGGAGATTTGCAAATTTCTCACCGTGATATTCGTCGCCATTGTCGTCCTCTACTCCGTCCAGGCAGCAGAACAAGGAGGAGATCATCATTCGATGGCTTGCATGCAAAAACTTATGCCGTGTCAAAACTATATACACGCCGTGAATCCGGCGCCTCCAGCGTCATGTTGCGGACCAATGAAAGAGATCGTGGAGAAAGACTCTAAGTGTCTATGCACAGTTTTTAACAACCCGGAATTGCTCAAATCACTCAACCTCACCAAAGAAAATGCCCTTGATCTTCCCAAGGCCTGTGGAGTTAATCCTGACGTCTCAATCTGCACCAAAACCGCTTGTAAGTTTTTTATTATTTTTTCAACTAAAATCCCACATATATATTTTATTTAA |
| *BrnsLtpIX.1* | ATGATTCTTATGGTTTTGGTCGAGTCAGGCCTGCTCAAAGAGGCTACGGCACACCCATGTGGTCGAACATTTTTGTCCGCTTTGATTGAACTCGTGCCTTGTACACTATCAGTGGTGCCGTTTAGCACGTTGTCTCCGAACGAGCCATGTTGCACTGCCATCAAAACGCTTGGTCAGCCTTGTCTATGCGTTATTGCCAATGGACCATCTATTCCTGGCGTTGACCACACTCTCGCTCTCCAGTTACCTGGAAAATGTTCTGCCAATTTTCCTCCGTGTAACTAA |
| *BrnsLtpIX.2* | ATGAAAGCCATGAGAGTTGGGTTAGCTATGGCACTGCTCATGACCATAACGGTTCTTACCATAGTCACTGCACAGCTCGAGGACCAGCAACCACCTCCACCGATGTTACCCGAGGAAGAAGTGGGAGGATGCAGCAGAACATTCTTCTCAGCACTGGTTCAGCTGATACCGTGTAGAGCAGCAGTGGCTCCTTTCAGCCCAATCCCACCGACCCAGTCATGTTGCTCTGCAGTTGTCACACTTGGTCGCCCTTGCCTTTGCCTCCTCGCCAATGGACCTCCACTATCAGGCATTGACCGCTCCATGGCTCTTCAGCTTCCTCAGAGATGCTCTGCAAATTTCCCTCCTTGCGATATCATTAACTAG |
| *BrnsLtpIX.3* | ATGATGATGAGACCCATGAGAGTTGGGTTAGCAATGGCACTGCTCATGACTATAACCGTTCTTACCATAGTCATAGCACAGCAAGAGGACCAGCAACCACCTCCACCGATGTTGCCAGTGGAAGAAGTGGGAATGTGCAGCAGAACATTCTTCTCGGCTCTGGTTCAGCTGATACCGTGTAGAGCAGCAGTTGCTCCTTTCAGCCCAATCCCACCAACCGAGTCGTGTTGCTCGGCGGTTGTCACACTTGGTCGTCCTTGTCTTTGCCTCCTTGCCAATGGACCTCCACTCTCAGGCATTGACCGCTCCATGGCTCTCCAGCTTCCTCAGAGATGCTTTGCTAATTTCCCTCCATGTGATGTCATTAACTAG |
| *BrnsLtpXI.1* | ATGGCTGCAAAGACCTCAACCATACTTATTACTATATTCCTTGTAATCAATCTTCTCTTCCTCAACTTCATTCCCCTGGTTGTGGCAGAAAATACTTGTCCAAGAGACCAGCTCAAACTTTCAACTTGCGCAAATATTCTCAACCTCATCAACTTGAATCTTGGCGCACCAGCTATGAGACCTTGTTGCTCTGTTCTCTTAGGTCTAATCGATCTTGATATTGCGCTTTGCTTCTGTAGCGCGCTTAAGCTCAGCATTCTCGGCATCACCACCAACACTCCCATTCACCTTAACTTGGCCCTCAACGCCTGTGGAGGAACGCTTCCCGATGGATTTCGTTGCCCAACATAG |
| *BrnsLtpXI.2* | ATGGCTCCAAGAACCTCTCTTGCACTCTTCCTTTTCCTTAACCTCCTCTTCTTCACTTACACTACTGCTCAAGGCACTTGTCCTAGAAATGCCCTTCAGATCGGTGCTTGCACTAATGTGCTCAATGCAATTGACTTAACATTAGGAAACCCACCGCCACCAGTACCGCCGTGCTGCTCGCTCATTGCAGGCTTGGCTGACCTTGAGGCTGCAGTCTGTCTCTGTACCGCGCTGGACGTTAACGTTCTTGGCAACAACGTTCACCTTCCCATCGATATCAGCGTACTTTTAAATGCTTGTAGCAGATTTGCTCCACCAAGTTTCCAATGCCCGTAA |
| *BrnsLtpXI.3* | ATGGCTATCTCAAAAGCTTTTCCATTGCTTCTGGTCTTGTTGCTAGTCCTCAACTCAACATTCTCCTTTTGTCATGCCGTTAAGCAATGCCCTCCTCCCAGAAAACAATCATCCATGAAATGTCCAAGAGACACGGTCAAGTTTGGAGTCTGTGGCAGCTGGTTAGGTCTAGTTCATGAGGTCATCGGTACACCACCGAGCCAGGAGTGTTGCTCACTTGTCAAAGGTTTGGCTGATCTTGAAGCTGCCCTTTGTCTTTGCACCGCCCTTAAAACCAGCCTACTCGGAGTCGCTCCGGTTAAACTTCCTGTTGCTCTCACTTTGCTTCTCAACTCTTGTGGCAAGACTCTTCCTCAGGGATTTGTCTGTTGA |
| *BrnsLtpXI.4* | ATGGCTTATTCTAAGATTGCTCTCCTCCTCATTTTGAATGTCATTTTCTTCACCTTGGTCAGCTCGAATCCAGTCCCTTACCGCAAACCCACTTGTAAAAATGCTCTTAAATTCAAGGTGTGTGCCAATGTGTTGGATTTGGTTAAGGTTTCTCTACCAACAAGATCCAAATGTTGCGGACTTATCAAAGGTCTAGTTGATCTCGAAGCTGCAGTTTGTCTCTGCACCGCCTTAAAAGCTGATCTCCTTGGCCTCAAACTAAATGTTCCCATTTCATTGAGTGTTATCTTAAACCACTGTGGTAAGAAGGTTCCATCTGGTTTCAAATGTGCCTAG |
| *BrnsLtpXI.5* | ATGGCTTATTCTAAGATTGCTCTCCTCCTCATTTTGAATGTTATTTTCTTCACCTTGGTCAGCTCGAATCCAGTCCCTTACCGCAAACCCACTTGTAAAAATGCTCTTAAATTCAAGGTGTGTGCCAATGTGTTGGATTTGGTTAAGGTTTCTCTACCAACAAGATCCAAATGTTGCGGACTTATCAAAGGTCTAGTTGATCTCGAAGCCGCAGTTTGTCTCTGCACCGCCTTAAAAGCTGATCTCCTTGGCCTCAAACTAAATGTTCCCATTTCATTGAGTGTTATCTTAAACCACTGTGGTAAGAAGGTTCCATCTGGTTTCAAATGTGCCTAG |
| *BrnsLtpXI.6* | ATGGCTTATTCTAAGATTGCTCTCCTCCTCATTTTGAATGTCATTTTCTTCACCTTGGTCAGCTCGAATCCAGTCCCTTACCGCAAACCCACTTGTAAAAATGCTCTTAAATTCAAGGTGTGTGCCAATGTGTTGGATTTGGTTAAGGTTTCTCTACCAACAAGATCCAAATGTTGCGGACTTATCAAAGGTCTAGTTGATCTCGAAGCCGCAGTTTGTCTCTGCACCGCCTTAAAAGCTGATCTCCTTGGCCTCAAACTAAATGTTCCCATTTCATTGAGTGTTATCTTAAACCACTGTGGTAAGAAGGTTCCATCTGGTTTCAAATGTGCCTAG |
| *BrnsLtpY.1* | ATGAAGATACATCGTGCCATAATCCTGGTGACACTCTTGGCTTTGATCAAGACCGCCGTTTCCCAACTTCAATCGATTGAGCAGTGCAGAGAGGTGTTCGACAGTTTCATGCCGTGCATGGGCTTCGTCGAGGGAATCTTCGAGCAACCTTCTCCACAATGCTGTAGAGGCGTGAGCCACTTGAACAATGTCGTCAAGTTCAAGACTCCAGGATCGAGGAAGAATGAGCAGGGGACTGGACAGTTAGAGAGAGTATGCGAATGCATAGAGATGATGGGAAAATCAGATCATCTTCCTTTTCTGGCATCCGCCATCAACAATCTTCCTCCCCTTTGTTCTCTTTCGCTCAGCTTTCCTATCTCTGTTGGAATGGACTGTTCTCAGTTTAGAAACATGAAGGAACTCGACGCTGAAAAAGTAAACTAG |
| *BrnsLtpY.2* | ATGATGATGATGAGAGTAGCGTTTGCGATGACGTGCATGCTGTTTGCAATCACAACCGCCGACAAGGGTGACCGTCCCTGGCCCCGTGAGTGCCTAGAGGTGGCGAACGTTATGGTGGAAGAATGCAAGCTGTTCTTCGTCGAACAGGAATCGCCTCCCACCGCCGAGTGCTGCGGCTGGTTCAGTAGCCGCCGTGAAAAGGCTAAGGACAGGAGGCGGATTTGCCGGTGTATGGAGTTTCTGACCACAGCCTTTGAAGCAATCAAGCCAAGCGTTTTGGCTCTCTCTGACCAGTGCCATTTCGGCGGTGGCTTCCCCATTTCAAAAAACCATGCATGCGCTTGTAAGCTCCATTCCTTTGACCAAACTCTTTAA |
