# Supplementary material for: Genome-Wide Survey and Expression Analysis of the Putative Non-Specific Lipid Transfer Proteins in Brassica rapa L
Source: PLoS One. 2014 Jan 31;9(1):e84556. doi: 10.1371/journal.pone.0084556 (PMC3908880; doi:10.1371/journal.pone.0084556)
Supplement: Table S2 — The protein backbones of BrnsLtps. (DOCX) [file pone.0084556.s006.docx]

**Table S2.** The protein backbones of BrnsLtps

| **Name** | **Protein backbones** |
| --- | --- |
| **BrnsLtpI.1** | MAGLMKLACFLVACMIVAGPITANAALTCASVVSNMARCISYLGGSETISGACCSGIRSINGLSRTPSDRQIACGCLKRVATLPNINADRAAGLPNACGVSLPYNISKSANCTLYVDFNLSRYLSLSL |
|  | |
| **BrnsLtpI.2** | MRSLFLLALFLVLAFHHGEAAVTCNNVVGDLYPCLSYVMQGGNSPSTNCCSGVRTLNSQAQTTADRQSVCRCIKNAIGGASYSSSNLKNALSLPAKCGVNLPFSISPSTNCNR |
|  | |
| **BrnsLtpI.3** | MAGLVKLACLVLACMIVAGPITSKAALSCGTVNTNVAACIGYLTQGGPLPRACCTGVSKLNSIARTTPDRKQACRCLKTAASALGSGLNAGRAAGLPKACGVNVPFPISTSINCNGHREISSVRMKLEWIIPML |
|  | |
| **BrnsLtpI.4** | MAGLMKLACLVLACMIVAGPITSNAALSCGTVSGYVAPCIGYLAQGAPALPRACCSGVTSLNNLARTTPDRQQACRCLVGAANAFPTLNAARAAGLPKACGVNIPYKISKTTNCNSVK |
|  | |
| **BrnsLtpI.5** | MALALRFFTCLVLTVCIVASVDAAVSCGTVTSSLAPCANYLSKGGDVPPPCCAGVTKLNGMAQTTPDRQQACKCLQSAAKTVSGLDPSLAAGLPGKCGVSIPYPISMSTNCDNVK |
|  | |
| **BrnsLtpI.6** | MASTLRFLTCLVTVCIVASVGAPISCGTVVQSMSPCITYLSGRMDLTAACCGGVRDLNAIAQTTPDRQQTCKCLQAVAKKIPGFNQTRASDLPGKCRVSFPFPISISTNCDNVHHEDEEYMVYVQ |
|  | |
| **BrnsLtpI.7** | MMFPSKITTCLLVLAVYMASPSESTITCGTVTSTLARCIGYLTNSGSLPSDCCVGVKSLNQMAQTTPDRRQVCECLKSAAKDITGLNTDLVATLPTTCGVSVPYPIRFSTNCDTISTAV |
|  | |
| **BrnsLtpI.8** | MAGVMKLVCLVLACMIVAGPITANAALTCGTVNSNVAPCIGYITQGGPLPGACCTGVSKLNSMARTTPDRQQACRCLKTAASALGPSLNAGRAAGIPKACGVSVPFPISTNTNCNSVK |
|  | |
| **BrnsLtpI.9** | MAGLMKLACLVLACMIVAGPITTNAALSCGTVSGNLAACIGYLTQNGPLPRGCCTGVTNLNNMARTTPDRQQACRCLVGAANSFPTLNAARAAGLPKACGVNIPYKISKSTNCNSVR |
|  | |
| **BrnsLtpI.10** | MAIALRFFTCLVLTVCIVASVDAAITCGTVTSSLAPCATYLSSGGEVPPPCCAGVKKLNGMAQTTADRQQACKCLKAAAQGINPSLASSLPGKCSVSIPYPISMSTNCDNVK |
|  | |
| **BrnsLtpI.11** | MTMGLKFFTCLVLTVCIAASVDAALTCGTVTSSLAPCATYLSKGGAVVPGPCCAGVKKLNDMAQTTPDRQQACKCLKAAAKSINPSLASGLPGKCSVSIPYPISMSTNCDK |
|  | |
| **BrnsLtpI.12** | MAYSKIALLLILNVIFFTLVSSNPVPYRKPTCKNALKFKVCANVLDLVKVSLPTRSKCCGLIKGLVDLEAAVCLEAAVCLCTALKADLLGLKLNVPISLSVILNHCGKKVPSGFKCA |
| **BrnsLtpI.13** | MASALSFFTCLVLTVCIVASVDAAISCGTVTSNLVPCAGYLMKGGPVPASCCAGVSKLNSMAKTTPDRQQACKCLKTAAKSVNPSLASSLPGKCGVSIPYPISMSTNCNT |
|  | |
| **BrnsLtpI.14** | MRSLLLALFLVLAFHRGEAAVSCNAVVGDLYPCLSYVVQGGNVPANCCNGIRTLNSQAQTPVDRQGVCRCIKNAIGGVSFSSNNVNNAQSLPAKCGVNLPYSISPSTNCDR |
|  | |
| **BrnsLtpI.15** | MEGLLKLSTLVIVCMLVSAPMASEAAISCGAVASNLGQCINYLTRGGFVPRGCCSGVRRLNSMARTTRDRQQACRCIQGAARALGSRLNPGRAARLPGACRVRIAYPISARTNCNK |
|  | |
| **BrnsLtpI.16** | MAFASKIITCLLVLKVYMAAPAESHITCGIVTSTLAQCMGYLTNFFPVPSDYCCAEVKGLNQMAQTTPDRRQVCKCLKAVAKENKGFISIELVGTLPTICGVSVPYPFNFSTNCDTISTAV |
|  | |
| **BrnsLtpI.17** | MSILKSLVTIFVLGIFLTPRYSESAISCSVVLSDLQPCVSYLTSGSGQPPETCCDGVRSLDAATTTSADKKAACQCIKSVANSVTVKPELAKALASNCNASLPVDASSTVDCNTVG |
|  | |
| **BrnsLtpI.18** | MTFASKIITCLLVLTIYIAAPTESHITCGTVTSTMTQCISYLTNGGPLPSSCCVAVKSLNQMAQTTPDRRQVCECLKSAGKEIKGLNIDLVAALPTTCGVSLSYPIGFNTNCDSISIAV |
|  | |
| **BrnsLtpI.19** | MEGFIKLSTLLIVCMLVSAPMAEAAISCGAVASNLGQCINYLTRGGFVPRGCCSGVQRLHSMARTTRDRQQACRCIQGAARALGSRLNPGRAARLPGACRVRISYPISARTNCNK |
|  | |
| **BrnsLtpII.1** | MKFSCSKPVLFTCAILLLLIVAQENRVVAGQSCDPMQLIPCEEAILKGSKPSDTCCTRLNQQQHCVCQYMKNPNFKSFLDSPNAKKIATDCHCPKPKC |
|  | |
| **BrnsLtpII.2** | MKFTGVVCIAFVIVLVSASAPTKEVLEEKVACNSTEHITCIPALQSGSQPSAECCGKLKEEESCLCGYIQNPLFSQYVTSANVHMVLVTCGIPYPSC |
|  | |
| **BrnsLtpII.3** | MVKVMWGSSLALAAALLLVTVANIPVAEGVTCSPTELTSCSSAFMSASPPSATCCAKLREQKPCLCGYLRNPALSQYVNSPNAKKVASSCNVATPKC |
|  | |
| **BrnsLtpII.4** | MKASCTKPVLITCTILLLLIVAQENRVAAAEQCNPMQLMPCEDAIMKGSTPSNECCTRLKQQQHCICQYMKNPNFKSFLNSPNAKMVASHCQCKPKC |
|  | |
| **BrnsLtpII.5** | MNFTGAICIAFVIVLVSSLALTNAAVEDEKVLACNPKELNPCSPAVKTGSKPSTECCAMLKKEEPCLCGYINDPVYGQYIKSKNAHKAFSSCGIPPLSC |
|  | |
| **BrnsLtpII.6** | MEMIKAKWVSIVALAAIFLVVILVPAAEAVTCSPMQLSPCASAITSSSQTSALCCAKLKEQKPCLCGYMRNRSLRRFVSSPNARKVSNRCKLPIPRC |
|  | |
| **BrnsLtpII.7** | MVMIKATWVSIFAIAAVLLVILAPAAEAVTCSPMQLSPCAQAITSSSPPSALCCAKLKEQKPCLCGYMRNPSLRRFVSSPNARKVSNRCKLPIPRC |
|  | |
| **BrnsLtpII.8** | MKLTAIGLVAMVTIVVQLSPTMACDVKDLSPCLLPIAVFPESPTAACCQTLRDQGPCLCVFINNSWIWIGPTLTSPNGHKLFAACQVPFPSCGN |
|  | |
| **BrnsLtpII.9** | MKFTGAICIALVIVLVSSLDLTSAAVEEEIKVACVVTELIPCLESSIIGVHPYPECCVTLKAQQSCLCGYIQNPVYGGFFKNAHSVFTGCGVPYPTC |
|  | |
| **BrnsLtpII.10** | MKFTGAICIALVIVLVSSLDLTSAAVEEEIKVACVQTELIPCFVAAFIGSQPSAECCEKLKEQQSCLCGYISNPVFGQFYKNAQNVFKACGVPYPTC |
|  | |
| **BrnsLtpII.11** | MKFTTLMVITLVIIAMSSPVPIRATSVESFGEVAQSCVVTELAPCLPAMTTAGDPTTECCDKLVEQKPCLCGYIRNPAYSMYVTSPNGRKVLDFCKVPFPSC |
|  | |
| **BrnsLtpII.12** | MRFTGVVCIAFVIVLVSALAPTKADLEEKVACIPTELMTCIPALQTGSQPSAECCGKLKEQESCLCGYIQNPLFSQYVTSENAHKILATCGIPYPTC |
|  | |
| **BrnsLtpII.13** | MVKVMWVFVLALVAALLLVTVEKIPVAEGVTCSVTELSPCLAAFMSSSQPSASCCAKLREQKPCLCGYMRNPGLRQYVTSPNAKKVSNSCKVASPNC |
|  | |
| **BrnsLtpII.14** | MKFTTLASIAFVVVVLFSSTAAPINSQLIQSNSPCTTIDITGCVPAILYGAPLSPECCRNLNVQQPCYCDFIKNAGLKPYITSPQGHAALASCGIPYPTC |
|  | |
| **BrnsLtpII.15** | MVLTLMVFVILLTLFPAPNEAADTNVEAACDPKQLQPCLAAITGGGQPSGDCCAKLKEQQPCLCGFSKNPAFAQYISSPNSRKVLTACGIPYPSC |
|  | |
| **BrnsLtpIII.1** | MEFLKSFTTILFVMFLAMSALETVPMVRAQQCLDNLSNMQVCAPLVLPGAVNPAPNSNCCIALQATNKDCICNALRAATTFTTTCNLPSLDCGITI |
|  | |
| **BrnsLtpIII.2** | MVSISSSSKSSTIMKVVVMVAVVLVATVVDGQSCNTHLSGLNVCGEFVVPGADTTNPSAECCNALEAVPSDCICNTFRIASRLPTRCNIPTLSCN |
|  | |
| **BrnsLtpIII.3** | MIMKVAALVALVLVATEVDGQSCNRHLSGLNVCGEFVVPGADKTNPSAECCSALEAVPSECLCNTMRIASRLPTRCSIPTLSCS |
|  | |
| **BrnsLtpIV.1** | MDENNTRTIVAALVIVFVSLVLMEEPTSIPLCNINANTLEKCRPAVTGNNPPLPGDACCIVLQAADLECVCKFKSHIPILATKSHKVHDLLRKCGIKTIPPACQDKTKVS |
|  | |
| **BrnsLtpIV.2** | MGKNNTKILITALVMIVTASMMIEEAKSVRICNVSTKDLKKCRPAVTGNNPPPPTPQCCQLAKAANLECLCPFLSRSGIDPSKIKALGANCGITKNPSCLPW |
|  | |
| **BrnsLtpIV.3** | MGKNNTKILVTALVMVVTAAMMIEEATSIPICGVNTNDLKKCSPAVTGNNPPPPTPQCCKVAKAANLECLCPYFTRSGLDTAKIKALGTNCGITKKPSCLPW |
|  | |
| **BrnsLtpIV.4** | MASKKVGVMVMMMMIVVVMAIFAERSVAIDLCGMTQSELNECKPAVSKENPTNPSTLCCDYLKHADISCLCGYKNSPLLGSFGIDPALAAGLPTKCDMPNAPTC |
|  | |
| **BrnsLtpIV.5** | MAYVNKVSAVAAILFFAVAVAPLLAEPQTPMFPKMDPVCASLMPNLLEKCFSTVRETPTDDCCSDLKSATTTQVTCLCDNYIANPAVVNFTGPYSAGITTKCGVFDKYSCNGSSNGT |
|  | |
| **BrnsLtpIV.6** | MEPNTKLVVITLVLALTLTAATGEFCGMSVSDLYSCKPYVQSKNPVTSAIDPKGPCCTALSKADFQCLCKQKTKTNPFLSSIDLDLASKLPEKCGLSGATC |
|  | |
| **BrnsLtpIV.7** | MGKNNTTILIIAMVLTTAMIMEEAKSYPICNTDTNDLQKCSPAVTGNNPPAPGPDCCAVAKSADLECLCPYLSLSGIDPSKIKSVLASCGVGNPSCLSW |
|  | |
| **BrnsLtpIV.8** | MGKNNTNILTQSTVLAMVLTAAIMVKEVSSLTICKIDINDMQKCRPAVIGINPPPPVNECCVVVRSANLECFCGFKFYLPILGIDPSKVAALVAKCDVTTIPPSCQVSKVLASRSLKNGA |
|  | |
| **BrnsLtpV.1** | MKWCKFISVALMSLLITLASVEAAGECGRMPIGQAAASLSPCLAATKNPRGKVPPVCCAKVGALIRTNPRCLCAVMLSPLAKKAGINPGVAIAIPKRCNIRNRPSGKRCGRYIVP |
|  | |
| **BrnsLtpV.2** | MASFMKFLCVLGLFLLVGTVVDGAGECGRSTPDNEAMKLAPCVGAAQDANAAVPGGCCAQIKRFSQNPKCLCAVLLSDTAKASGVQPEVALTIPKRCNFANRPVGYKCGVLRVQIHGLYDEALEFTTTS |
|  | |
| **BrnsLtpVI.1** | MATGSRVLIGLAMILIISGELLVPGQGTCQGDIEGLMRECAVYVQRPGPKVNPSAACCKVVKRSDIPCACGRITPSVQKMIDMNKVVLVTSFCGRPLAHGTKCGSKSVIN |
|  | |
| **BrnsLtpVI.2** | MGSGMITVMVVAIAFFMIGSDNVNMATAQLCGANLSGLVNECQRYVSNAGPNSPPPSRSCCALIRPIDIPCGCRYVTRDVMNTFDMDKLIYVARSCGKKIPSGYKCGSYTIPAA |
|  | |
| **BrnsLtpVI.3** | MASGRIIIMVVAIAFFMIGSDNVNVATAQFCGANVSGLMNECQRYVSNAGPNSPPPSRSCCALIRPIDVPCACRYVSRDVTNYIDMDKVVYVARSCGKKIPSGYKCGSYTIPAA |
|  | |
| **BrnsLtpVI.4** | MASGRIIIMVVAIAFFMIGSDNVNVATAQFCGANVSGLMNECQRYVSNAGPNSPPPSRSCCALIRPIDVPCACRYVSRDVTNYIDMDKVVYVARSCGKKIPSGYKCGSYTIPAA |
|  | |
| **BrnsLtpVIII.1** | MEICKFLTVIFVAIVVLYSVQAAEQGGDHHSMACMQKLMPCQNYIHAVNPAPPASCCGPMKEIVEKDSKCLCTVFNNPELLKSLNLTKENALDLPKACGVNPDVSICTKTACKFFIIFSTKIPHIYFI |
|  | |
| **BrnsLtpIX.1** | MILMVLVESGLLKEATAHPCGRTFLSALIELVPCTLSVVPFSTLSPNEPCCTAIKTLGQPCLCVIANGPSIPGVDHTLALQLPGKCSANFPPCN |
|  | |
| **BrnsLtpIX.2** | MKAMRVGLAMALLMTITVLTIVTAQLEDQQPPPPMLPEEEVGGCSRTFFSALVQLIPCRAAVAPFSPIPPTQSCCSAVVTLGRPCLCLLANGPPLSGIDRSMALQLPQRCSANFPPCDIIN |
| **BrnsLtpIX.3** | MMMRPMRVGLAMALLMTITVLTIVIAQQEDQQPPPPMLPVEEVGMCSRTFFSALVQLIPCRAAVAPFSPIPPTESCCSAVVTLGRPCLCLLANGPPLSGIDRSMALQLPQRCFANFPPCDVIN |
|  | |
| **BrnsLtpXI.1** | MAAKTSTILITIFLVINLLFLNFIPLVVAENTCPRDQLKLSTCANILNLINLNLGAPAMRPCCSVLLGLIDLDIALCFCSALKLSILGITTNTPIHLNLALNACGGTLPDGFRCPT |
|  | |
| **BrnsLtpXI.2** | MAPRTSLALFLFLNLLFFTYTTAQGTCPRNALQIGACTNVLNAIDLTLGNPPPPVPPCCSLIAGLADLEAAVCLCTALDVNVLGNNVHLPIDISVLLNACSRFAPPSFQCP |
|  | |
| **BrnsLtpXI.3** | MAISKAFPLLLVLLLVLNSTFSFCHAVKQCPPPRKQSSMKCPRDTVKFGVCGSWLGLVHEVIGTPPSQECCSLVKGLADLEAALCLCTALKTSLLGVAPVKLPVALTLLLNSCGKTLPQGFVC |
|  | |
| **BrnsLtpXI.4** | MAYSKIALLLILNVIFFTLVSSNPVPYRKPTCKNALKFKVCANVLDLVKVSLPTRSKCCGLIKGLVDLEAAVCLCTALKADLLGLKLNVPISLSVILNHCGKKVPSGFKCA |
|  | |
| **BrnsLtpXI.5** | MAYSKIALLLILNVIFFTLVSSNPVPYRKPTCKNALKFKVCANVLDLVKVSLPTRSKCCGLIKGLVDLEAAVCLCTALKADLLGLKLNVPISLSVILNHCGKKVPSGFKCA |
|  | |
| **BrnsLtpXI.6** | MAYSKIALLLILNVIFFTLVSSNPVPYRKPTCKNALKFKVCANVLDLVKVSLPTRSKCCGLIKGLVDLEAAVCLCTALKADLLGLKLNVPISLSVILNHCGKKVPSGFKCA |
|  | |
| **BrnsLtpY.1** | MKIHRAIILVTLLALIKTAVSQLQSIEQCREVFDSFMPCMGFVEGIFEQPSPQCCRGVSHLNNVVKFKTPGSRKNEQGTGQLERVCECIEMMGKSDHLPFLASAINNLPPLCSLSLSFPISVGMDCSQFRNMKELDAEKVN |
|  | |
| **BrnsLtpY.2** | MMMMRVAFAMTCMLFAITTADKGDRPWPRECLEVANVMVEECKLFFVEQESPPTAECCGWFSSRREKAKDRRRICRCMEFLTTAFEAIKPSVLALSDQCHFGGGFPISKNHACACKLHSFDQTL |

“XXX”: N-secretion signal which is predicted by SignalP; “XXX”: Eight-cysteine motif (ECM); “X”: Cysteine residue in ECM; “XXX”: Consensus pentapeptides T/S-X_1_-X_2_-D-R/K and P-Y-X-I-S; “X”: Tryptophane residue.
